# High-density genetic map construction and mapping of the homologous transformation sterility gene (*hts*) in wheat using GBS markers

**DOI:** 10.1186/s12870-018-1532-x

**Published:** 2018-11-26

**Authors:** Qian Yang, Zaijun Yang, Haifeng Tang, Yan Yu, Zhenyong Chen, Shuhong Wei, Qinxu Sun, Zhengsong Peng

**Affiliations:** 1 Key Laboratory of Southwest China Wildlife Resources Conservation (ministry of education), College of Life Science, China West Normal University, Nanchong, 637009 Sichuan China; 2 School of Agricultural Science, Xichang University, Xichang, 615000 Sichuan China

**Keywords:** Wheat, Genotyping-by-sequencing, Pistillody, Genetic map, *Win* gene

## Abstract

**Background:**

Homologous transformation sterility-1 (HTS-1) is a novel wheat mutant that exhibits pistillody, the transformation of stamens into pistils or pistil-like structures. More extreme phenotypes of this mutation can have six pistils or pistil-like structures without any stamens in a floret. Thus, HTS-1 is highly valuable for studies of wheat hybrid breeding and flower development. Previous studies have shown that two major genes (*Pis1* and *hts*) control pistillody in HTS-1. The *Pis1* gene controls the three-pistil trait in the three-pistil wheat mutant and has been mapped on chromosome 2D, but the *hts* gene has not been mapped or identified. To do so, we crossed HTS-1 with CM28TP (three-pistil mutant) and constructed a high-density linkage map with the F_2_ population (200 individuals).

**Results:**

The map covered 2779.96 cM, and the genetic distance per chromosome ranged from 37.59 cM to 318.95 cM. The average distance between markers was 1.04 cM. We then mapped *hts* between GBS-SNP markers 4A_109 and 4A_119, separated by 2.0 cM and 5.2 Mb. To find the candidate genes, the *hts* region was enlarged to 7.2 Mb, encompassing 752 protein-coding genes. We identified *TaWin1* as a possible candidate gene after comparing the 752 genes with 206 common differentially expressed genes between pistillody stamens (PS) versus normal stamens (S) and pistils (P) versus S. Real-time PCR indicated that *TaWin1* was highly expressed in HTS-1 during the pistil-and-stamen-differentiating stage, at levels approximately 120 times greater than those in CM28TP. Further analysis indicated that *TaWin1* was mainly expressed in HTS-1 PS, supporting its status as a candidate gene of *hts*. Thus, *TaWin1* overexpression probably leads to the transformation of stamens into pistils in wheat.

**Conclusions:**

The results of this study provide a foundation for further research on stamen and pistil development, with implications for wheat-hybrid breeding programs.

**Electronic supplementary material:**

The online version of this article (10.1186/s12870-018-1532-x) contains supplementary material, which is available to authorized users.

## Background

Common wheat (*Triticum aestivum* L.) is a staple food crop worldwide, and approximately 11.02% of the global wheat cultivation area (22.16 million ha annually) is in China [1]. Current wheat yield in China averages approximately 7000 kg/ha and is far lower than that of rice or corn yield [1]. Crossbreeding to generate hybrids with superior traits (heterosis) is a promising method to improve wheat yield. Although progress has been made in developing hybrid wheat over the past few decades, the results have not been applied to production because a suitable sterile male line is currently lacking. Homologous transformation sterility-1 (HTS-1) is a novel wheat male-sterile mutant that was observed in the offspring of a cross of the three-pistil (TP) mutant with Chinese Spring (CS) [2]. HTS-1 and Chinese Spring three pistils (CSTP) are sib lines that show the three-pistil trait because they carry the *Pis1* gene. However, the HTS-1 phenotype is characterized by pistillody, the partial or complete transformation of stamens into pistils or pistil-like structures [3–5]. Some HTS-1 mutants with more extreme phenotypes even exhibit six pistils and lack stamens entirely, and their seed-setting rate is often very low, 15.3% on average, under the natural pollination condition [2]. HTS-1 differs from other pistillody mutants, such as alloplasmic line of wheat cultivar Norin 26 (N26) and (cr)-CSdt7BS, because the phenotype is caused by nuclear-cytoplasm interactions in the latter two lines [6, 7], with the MADS-box gene *TaAGL2* being a potential contributor to the trait [8]. In contrast, the pistillody trait in HTS-1 is determined by two recessive karyogenes [2]. We hypothesized that one of these genes is *Pis1* because the mutant exhibits TP traits. Unfortunately, the identification and fine mapping of functional genes in wheat has proven more difficult than that in diploid species, such as rice, because its genome is enormous (17 Gb) and very complex (allohexaploid: 2n = 6X = 42, with A, B, D genomes) [9].

The development of next-generation, high-throughput sequencing has allowed the sequencing of the entire wheat genome [9–11], accelerating the efficiency of wheat breeding using molecular marker-assisted selection (MAS) and providing valuable tools for gene mapping. Notably, genotyping-by-sequencing (GBS) is a highly accurate, cost-effective, reliable, and rapid method for assessing such large, complex genomes because genome complexity is reduced through multiple enzyme digestions [12]. Furthermore, GBS does not rely on prior genome information for genetic linkage map construction [13], and multiplexed GBS can reduce the cost per sample when used to generate high-density linkage maps for gene identification and fine mapping [14–18]. Indeed, we previously used GBS to map *Pis1* on chromosome 2D, between SNP markers M70 (3 cM away) and M71 (1.1 cM away) [19]. However, the second gene, *hts*, which controls pistillody in HTS-1, remains unidentified to date.

In the present study, we used GBS-SNP markers to construct a high-density linkage map of wheat on the basis of an F_2_ population derived from crossing HTS-1 with Chuanmai 28 three pistils (CM28TP). We used this linkage map to map another major gene (*hts*) controlling the pistillody trait. We expected our work to lay a foundation for the study of the development of stamen and pistil and hybrid breeding in wheat.

## Materials and methods

### Plant materials

**Fig. 1:**
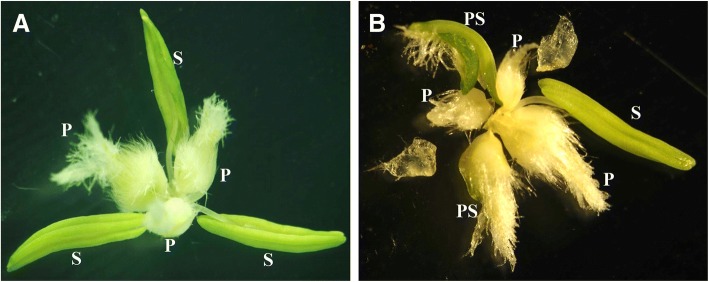
Morphology of the florets in CM28TP and HTS-1. A: Floral organs of CM28TP. CM28TP carrying *Pis1* gene, and showing three pistils and three stamens. B: Floral organs of HTS-1. HTS-1 carrying *Pis1* gene and *hts* gene, and exhibit that transforms all or parts of stamen into pistils or pistil-like structures. P: pistil; S: stamen; PS: pistillody stamen

HTS-1 is a novel wheat male-sterile mutant that was selected in the offspring of a cross of TP mutant with CS [2]. CM28TP was derived from (Chuanmai 28/TP mutant)//Chuanmai 28 by conventional breeding [20]. HTS-1 exhibits both three pistils (*Pis1*) and pistillody (*hts*), whereas CM28TP only exhibits three pistils (*Pis1*) (Fig. 1). HTS-1 and CM28TP were crossed to produce an F_2_ population of 200 individuals. To map *hts*, we first counted pistil-like stamens per floret for all 200 F_2_ individuals during the flowering period. Plants were categorized as pistillody mutant if any pistil-like stamens were present and plants were wild type otherwise. The following nulli-tetrasomic (NT) lines derived from Chinese Spring (CS) were used for verifying mapping results: N1AT1B, N1BT1A, N1DT1B, N2AT2D, N2BT2D, N2DT2A, N3AT3B, N3BT3D, N3DT3A, N4AT4B, N4BT4A, N4DT4B, N5AT5D, N5BT5A, N5DT5A, N6AT6B, N6BT6D, N6DT6B, N7AT7B, N7BT7A, and N7DT7A [21]. With the exception of NT lines from the United States National Plant Germplasm System (NPGS), all other experimental materials have been cultivated by our laboratory. All materials are stored at China West Normal University in Nanchong, China.

### DNA and RNA extraction

Total genomic DNA was extracted from the fresh leaves of F_2_, parental, and NT lines using a Plant Genomics DNA Kit (Tiangen Biotech, China) following the manufacturer’s protocol. Expression analysis of candidate genes was performed on young CM28TP and HTS-1 spikes at various developmental stages: double ridge to floret differentiation (spike length, 2–5 mm), pistil-and-stamen differentiation (spike length, 5–7 mm), as well as anther-lobe formation (spike length, 7–10 mm). Pistillody stamens (PS), pistils (P), and stamens (S) in HTS-1 at the heading stage were also used. Total RNA was isolated from young spikes, PS, P, and S using an EASYspin Plus Plant RNA Extraction Kit (Axygen, USA) following the manufacturer’s protocol. DNA and RNA quality were assessed via 1% agarose gel electrophoresis. Nucleic acid concentrations were determined in an ND-2000 spectrophotometer (Thermo Fisher Scientific, USA) by measuring the absorbance ratio of 260/280 nm.

### GBS library construction and SNP genotype calling

Genomic DNA (100–1000 ng) from the 200 F_2_ plants and two parental plants (HTS-1 and CM28TP) was subjected to GBS following published methods [19, 22]. Two restriction enzymes (*Mse*I, *Nla*III) were used. Digested fragments were ligated to barcoded forward adapters and common reverse adapters. For library construction, samples were PCR-amplified using Illumina primers with sequences complimentary to the adapters. Amplicons were sequenced on an Illumina Hi-seq 2000 platform at Beijing Novogene Bioinformatics Technology Co., Ltd. One library was double-loaded onto two lanes of the Illumina flow cell as technical replicates. All sequences obtained in this study were submitted to NCBI (accession number: SRP127844). The bioinformatics pipeline UNEAK was used to call SNPs from GBS results [23]. To ensure linkage map quality, GBS-SNPs were not used for final mapping if they exhibited significant distortion (chi-square 1:2:1 test, *P* < 0.001) and > 25% missing data.

### Construction of high-density genetic maps and mapping of *hts*

The genetic linkage map was constructed in JoinMap version 4.0 [24], excluding markers with obvious distortion from expected Mendelian segregation ratios of 1:2:1. Independent logarithm of the odds (LOD) thresholds of 3.0 were used to position markers on linkage groups. Linkage analysis and marker-order assignment were performed with regression mapping. Recombination fractions between markers were converted to map distances (cM) using the Kosambi mapping function [25]. Overly similar markers were considered degenerate bin markers [26]. Linkage maps were drawn in MapChart 2.2 [27]. All bin markers were BLASTn-searched against the bread-wheat genome, IWGSC1 + popseq.31.pep (ftp://ftp.ensemblgenomes.org/pub/plants/release-31/fasta/triticum_aestivum/pep/). A co-linear analysis was performed on the genetic maps. The phenotypic data of the F_2_ individuals and the SNP markers on chromosome 4A were combined to map the *hts* gene using JoinMap version 4.0 [24].

### Verification of *hts* mapping results and possible candidate gene identification

Mapping results were verified by hybridizing HTS-1 with 21 NT lines to check for pseudodominance in F_1_ and to determine the chromosomal location of *hts*. Sequences of markers flanking *hts* were aligned to IWGSC1 + popseq.31.pep, and all genes found in between were considered candidates. Candidate genes were compared with 206 common differentially expressed genes (DEGs) across PS versus S and P versus S. These DEGs had been previously identified in an RNA-seq study of HTS-1 [28].

### Cloning of the possible candidate gene from CM28TP and HTS-1

Specific PCR primers were designed using Primer Premier 5.0 on the basis of the possible candidate gene sequence. The primer sequences were as follows: TaWin1–1F: 5′-CTGGCTAACCATCAGCAGTCC-3′ and TaWin1–1R: 5′-GGTCAAATCAAATCAAGAGGGAGT-3′. PCR amplification was done in a T-100 Thermal Cycler (Bio-Rad, USA) and reactions consisting of 100 ng template DNA, 25 μL 2 × PCR Mix (Tiangen Biotech, China), and 0.5 μM of each primer in a final reaction volume of 50 μL.

The PCR cycling conditions were as follows: initial denaturation at 94 °C for 3 min, followed by 40 cycles at 94 °C for 45 s, 58 °C for 45 s, 72 °C for 60 s, and a final extension at 72 °C for 10 min. The resulting amplified products were visualized by gel electrophoresis in 1.2% agarose gels. The specific DNA band was recovered using AxyPrep DNA Gel extraction kit (Axygen, USA). Purified PCR products were cloned into the pMD-20 T vector (TaKaRa, China), according to the manufacturer’s instructions. Transformants were plated on Luria-Bertani agar containing ampicillin. Fifteen positive clones for each material were screened and sequenced by Sangon BiotechCo., Ltd. (Shanghai, China).

### Expression analysis of the possible candidate gene by real-time PCR

The real-time PCR Primers were designed using Beacon Designer version 6.0 to amplify the 82 bp *TaWin1* (wound-induced protein 1) fragment (TaWin1-2F: 5′-TGGCTAACCATCAGCAGTCCC-3′ and TaWin1-2R: 5′-AGGCGCAGCACGAGGAACT-3′). Real-time assays were performed with SsoFast EvaGreen (BIO-RAD, USA) in the Bio-Rad CFX96 real-time PCR platform. All samples were analyzed in three biological replicates and the fold change in RNA transcripts was calculated using the 2^ΔΔct^ method [29]. Wheat ubiquitin (DQ086482) and actin (AB181911) were selected as reference genes [20].

## Results

### Sequencing of the parental lines and the F_2_ population

Using GBS, CM28TP and HTS-1 were sequenced at effective sequencing depths of 44.7-fold and 54.96-fold, resulting in 74,503,950 and 49,266,501 clean reads mapped to the bread wheat genome, respectively (mapping rates: 99.08 and 98.51%). The average *Mse*I enzyme capture rate was high (98.2%) across GBS data of the F_2_ population, validating digestion quality. We obtained 939,256,850 clean reads from F_2_, averaging 4,696,284 reads per individual. The average GC content was 42.5%, with a Q20 score of 96.8%. We screened 1,457,623 SNPs (homozygous: 543,979; hybrid: 913,644) from CM28TP and HTS-1. As the parental lines are homozygous, only the *aa × bb* genotype (53,352 SNPs) was used for further analysis. After filtering the low-coverage (< 75%) sequences from F_2_, 3316 candidate SNPs were obtained. Significantly distorted SNPs were filtered out (χ^2^ test, *P* < 0.001) to yield 3108 SNPs for determining bin markers.

### Genetic linkage map with GBS-SNP markers

We mapped 2684 GBS-SNP bin markers to 21 linkage maps (Additional file 1: Fig. S1). The genetic map was 2779.96 cM long, and the average distance between two markers was 1.04 cM. Of all mapped chromosomes, 2A contained 368 markers, the highest percentage (13.7%), while 4D had the lowest with 11 markers (Table 1). Chromosomal genetic distances ranged from 37.59 cM (5B) to 318.95 cM (2A), and their maximum gaps ranged from 10.08 cM (1A) to 18.21 cM (6B) (Table 1). Among the 2684 markers, 1158 covered 1163.49 cM in wheat genome A, whereas 1119 covered 1043.35 cM in genome B. Only 407 were located in genome D, covering 573.12 cM (Table 1). We observed 137 gaps of > 5 cM among all 21 chromosomes: 55 were in genome A, 46 in B, and 36 in D.

**Table 1:** Marker information for the high-density genetic map

Chr.	No. Bin marker	Genetic distance (cM)	Average distance (cM)	Max. gap (cM)	< 5 cM gap
1A	218	218.57	1.00	10.08	210
2A	368	318.95	0.87	17.24	357
3A	94	124.20	1.32	14.35	83
4A	81	105.70	1.30	12.74	73
5A	164	148.36	0.90	13.22	157
6A	104	63.85	0.61	14.76	99
7A	129	183.86	1.43	15.10	114
1B	79	70.99	0.90	13.58	73
2B	271	235.91	0.87	17.81	259
3B	215	204.61	0.95	12.75	209
4B	101	110.44	1.09	15.20	94
5B	32	37.59	1.17	11.71	29
6B	287	248.65	0.87	18.21	277
7B	134	135.16	1.01	15.84	125
1D	36	60.79	1.69	13.24	31
2D	97	71.08	0.73	16.25	93
3D	27	40.78	1.51	13.01	24
4D	11	66.19	6.02	16.52	3
5D	102	136.24	1.34	11.82	91
6D	96	117.49	1.22	16.58	90
7D	38	80.55	2.12	13.45	32
total	2684	2779.96	1.04	18.21	2523
A	1158	1163.49	1.00	17.24	1093
B	1119	1043.35	0.93	18.21	1066
D	407	573.12	1.41	16.58	364

A BLASTn search against the wheat genome verified all 2684 GBS-SNP markers in the linkage map (Additional file 1: Table S1). Additionally, a co-linearity analysis showed that all markers were consistent with the reference wheat genome, indicating highly accurate inference of the genetic recombination rate (Additional file 1: Figure S2). Overall, the genetic map constructed with GBS-SNP markers had sufficient coverage over the wheat genome, and most SNPs occurred in the same order as their corresponding chromosomes on the physical wheat-genome map.

### Mapping of *hts*

**Fig. 2:**
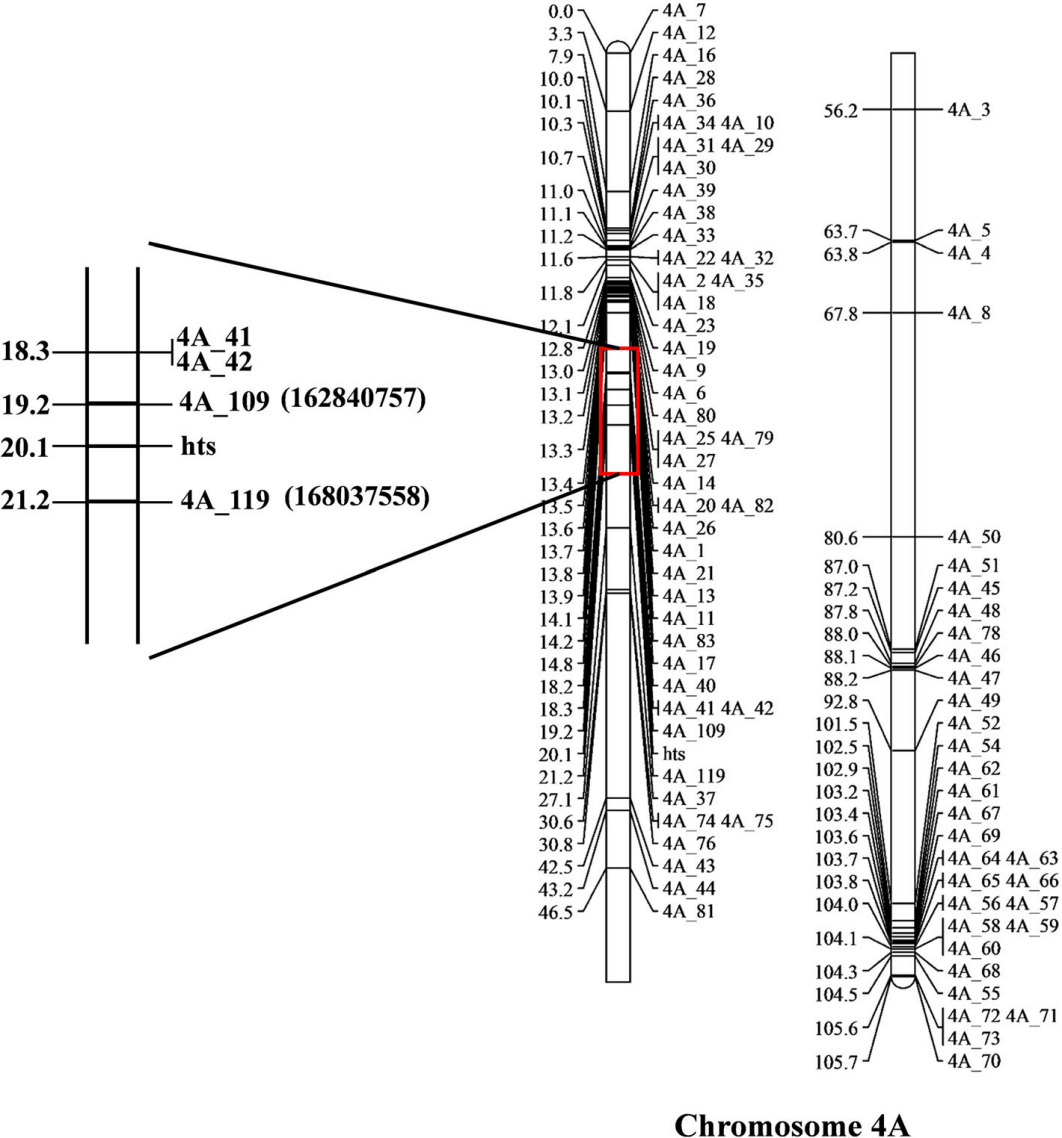
The genetic linkage map of the *hts* gene. Genetic distances are indicated on the left side of linkage group in centiMorgans (cM), and the marker names are shown on the right side. The physical distances are shown in the brackets

Among the F_2_ population, 45 individuals showed the pistillody trait and 155 individuals showed normal stamens, the pistillody-to-normal fit to Mendelian segregation ratio 1:3 (χ^2^ = 0.66, *P* > 0.05), indicating that a recessive effect gene controls the pistillody trait in HTS-1 × CM28TP plants (Additional file 1: Table S2). The genetic linkage map of chromosome 4A was constructed using stamen phenotypic (normal or pistillody) and SNP data. Two SNP markers were found to be tightly linked to the *hts* gene, namely, 4A_109 and 4A_119, with genetic distances of 0.9 cM and 1.1 cM from *hts*, respectively. These two markers are separated by a genetic distance of 2.0 cM and a physical distance of 5.2 Mb (Fig. 2). The chromosomal location of *hts* was further confirmed when all 11 F_1_ individuals of the HTS-1 × N4AT4B cross exhibited pistillody.

### Candidate gene prediction

Candidate genes were identified from an enlarged 7.2 Mb region (5.2-Mb interval and outer regions extending 1 Mb from GBS-SNP markers 4A_109 and 4A_119) encompassing 752 protein-coding genes (Additional file 1: Table S3). Comparison of the candidate genes with the DEGs showed that a possible candidate gene, Traes_4AS_F516F49FA.4 (physical distance: 162551918–162,552,664), shared 100% sequence identity with DEG comp83842. The latter was upregulated in PS and P, with log2 fold-change values of 5.75 and 5.46 for PS versus S and P versus S, respectively. A search against the NCBI nucleotide database (http://www.ncbi.nlm.nih.gov) revealed that Traes_4AS_F516F49FA.4 shared 96% sequence identity with *Win1* of *Aegilops tauschii* (GenBank accession number: XM 020340959.1), leading us to tentatively designate the candidate possible gene as *TaWin1*.

**Fig. 3:**
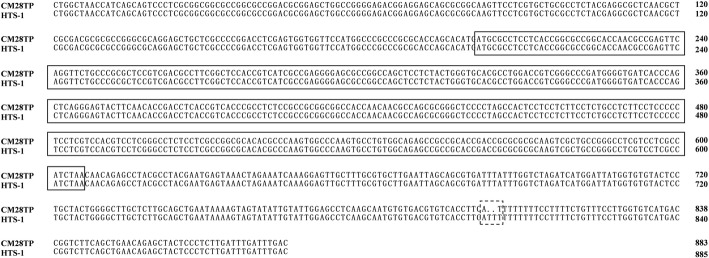
Alignment of the TaWin1 sequence in CM28TP and HTS-1. The ORF region and variation site are indicated by solid line frame and dotted line frame

PCR using DNA from fresh leaves of CM28TP and HTS-1 and the TaWin1–1 primer pair yielded a fragment of approximately 900 bp in length. The sequencing result indicated that the length of the *TaWin1* gene in CM28TP and HTS-1 was 883 bp and 885 bp, respectively. The open reading frame (ORF) was 408 bp and had no intron. The sequence similarity of *TaWin1* in CSTP and HTS-1 was 99.77%, with the only differences being two thymine (T) nucleotides inserted downstream of the ORF in HTS-1 (Fig. 3).

**Fig. 4:**
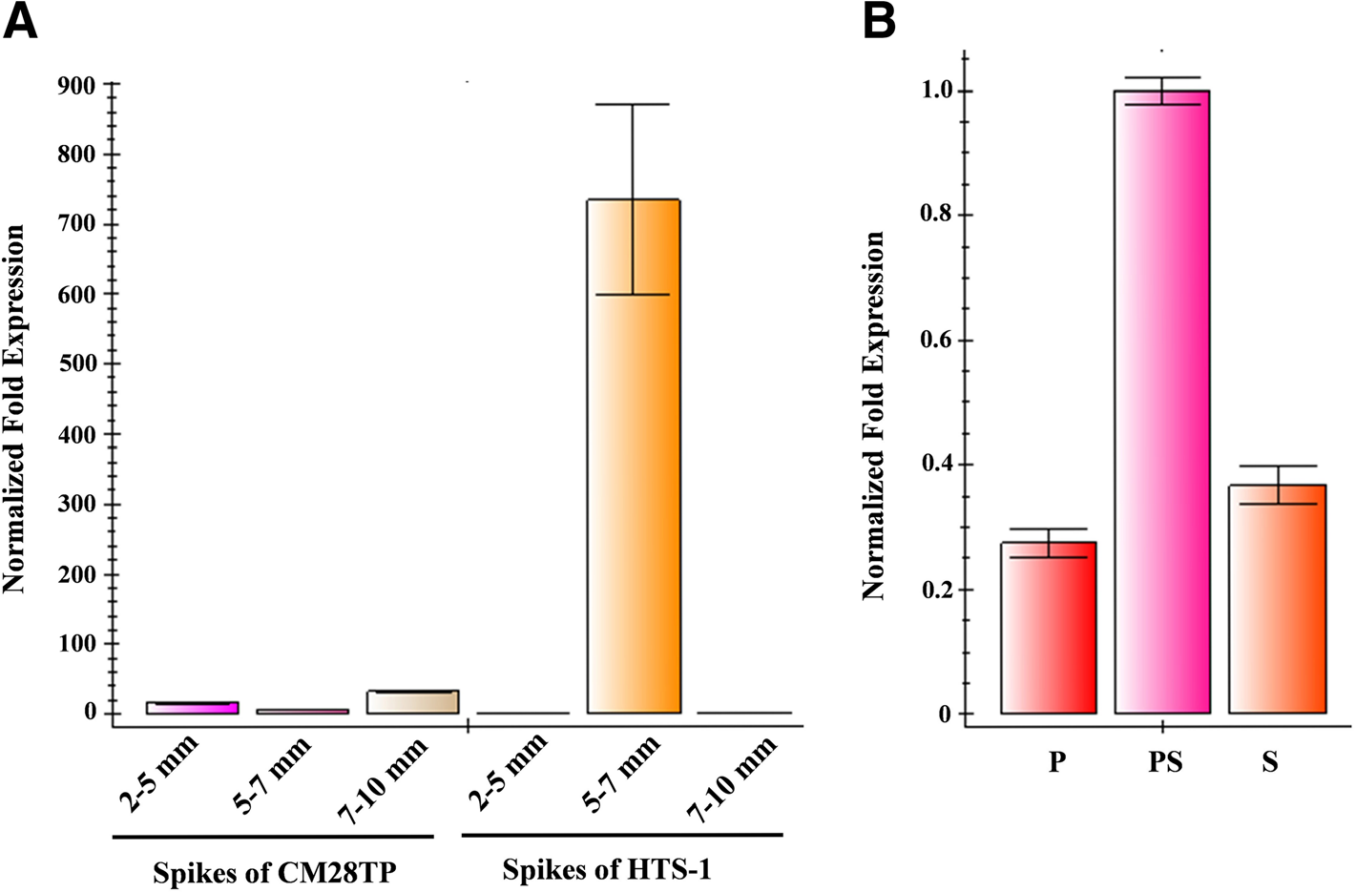
*TaWin1* gene expression assessed by real-time PCR. A: Relative levels of *TaWin1* gene in young spikes of CM28TP and HTS-1 at various development stages. B: Relative levels of *TaWin1* gene in different tissues of HTS-1. The transcript levels are shown as relative values and columns represent the means ± SEM of three replicates. P: pistil, S: stamen; PS: pistillody stamens

Real-time PCR demonstrated that during the pistil-and-stamen-differentiating stages, *TaWin1* was expressed in HTS-1 at 120 times the level in CM28TP (Fig. 4a). *TaWin1* expression was much lower in other stages of HTS-1 and CM28TP development. Additionally, *TaWin1* expression in HTS-1 PS was about 3.6-fold and 2.7-fold higher than in P and S, respectively (Fig. 4b).

## Discussion

The main objective of plant breeding is to develop high-yielding varieties to increase crop productivity to feed a growing human population. In rice and maize, the shift to hybrid breeding has facilitated large increases in yield [30]. In recent years, the potential of hybrid breeding in wheat has received renewed interest and is considered as a potential strategy to increase yield and to enhance yield stability [31]. Despite the progress made in hybrid wheat breeding, it is still in its infancy and a number of issues remain to be solved. Among them, the most important issue is the lack of a suitable, maintainable male-sterile line in wheat. HTS-1 is a novel pistillody mutant in wheat, and its stamens partially or completely transform into pistils or pistil-like structures [2]. Although the seed-setting rate of HTS-1 is 15.3% on average under natural pollination condition [2], our recent studies have shown that pistillody in HTS-1 is controlled not only genetically, but also by the environment. HTS-1 can be made completely male sterile by appropriately elevating the temperature at the booting stage (data not shown). Therefore, we speculate that HTS-1 is a male-sterile line that is controlled genetically as well as by temperature, and this can be applied in wheat-hybrid breeding. Using HTS-1 plants as female parents to cross with normal wheat material Chinese Spring, the seed-setting rate was reached about 30% by hand pollination [2].

Previous studies indicated that the *Pis1* and *hts* genes contribute to the pistillody trait in HTS-1 [2], and *Pis1* has been fine mapped on chromosome 2D of wheat [19]. In this study, we used GBS to map the *hts* gene, one of the two major genes controlling pistillody in HTS-1. This technique addresses issues with previous attempts using simple sequence repeats (SSR), which did not uncover markers linked to *hts* (data not shown). This failure was likely because too few SSR markers are present on the wheat chromosomes; for example, only 53 SSR markers (covering 88 cM) are located on chromosome 4A [32]. GBS is a highly accurate, cost-effective, reliable, and rapid method for assessing large, complex genomes, such as those of wheat and barley, for SNP discovery, and for genotyping [12]. The greatest advantage of GBS is that it does not rely on prior genome information for genetic linkage map construction [13], although imputation of SNPs can become more accurate in bi-parental mapping populations when a reference genome is available for the tested plants [33]. Thus, we took advantage of the availability of a wheat genome sequence in this study using CM28TP and HTS-1 as the parental lines. The effective sequencing depths of the two parents were 44.7-fold and 54.96-fold, respectively, with a sequencing error rate of only 0.03%, suggesting high-quality genome sequences. Furthermore, the relatively high error rates of low-coverage sequence data did not noticeably affect genotype-calling accuracy [33].

The two parental mutant lines were cultivated in our laboratory to create an appropriate mapping population for constructing a high-density linkage map [2, 34]. The originating line of HTS-1, CSTP, belongs to a population subgroup distinct from CM28TP, based on an analysis with sequence-related amplified polymorphism (SRAP) markers [34]. Thus, the founder parents were expected to yield F_1_ hybrids sufficiently heterozygous for generating an informative F_2_ mapping population. Additionally, *Pis1* interference can be excluded when mapping *hts* because both parents possess *Pis1*. We screened 1,457,623 SNPs from CM28TP and HTS-1; from these, 2684 polymorphic bin markers were identified for high-density linkage map construction. Bin-marker distribution across 21 linkage groups corresponded to the base chromosome number (*n* = 21) of common wheat. The total map length (2779.96 cM) was longer than a previous CM28 × CM28TP linkage map (2371.4 cM) constructed on the basis of 200 F_2_ plants and using 1987 bin markers [19]. The 2684 SNPs markers were identified across the three wheat genomes A, B, and D. The highest number of markers was found in the A genome, while the lowest was found in the D genome. In particular, only 11 markers were found for 4D. This poor representation of the D genome indicates a lower genetic diversity due to the low frequency of recombination and polymorphism [35, 36].

Using the constructed linkage map, we were able to identify and map *hts*, locating it on chromosome 4A between GBS-SNP markers 4_109 and 4A_119 (genetic distance: 2.0 cM, physical distance: 5.2 Mb). We then identified a gene, *TaWin1*, upregulated in HTS-1 PS and P. The sequence of *TaWin1* showed high similarity in CM28TP and HTS-1, in particular, their ORF sequences were identical, with only two T nucleotides inserted downstream of the ORF in HTS-1. However, this gene had unusually high expression during the pistil and stamen-differentiating stages of HTS-1 and was primarily expressed in PS. Therefore, we speculate that the pistillody trait is not caused by a change in the amino acid sequence of TaWIN1 but may be related to the overexpression of the *TaWin1* gene in wheat. It remains to be studied whether the insertion of the two T nucleotides downstream of the ORF in HTS-1 is the cause of *TaWin1* overexpression.

The *Win* gene was first isolated from potato and *Win* is mainly involved in wound-healing mechanisms. Ethylene upregulates *Win*-encoded mRNA [37, 38]. Numerous studies have shown that exogenous ethylene induces male sterility (cytoplasmic, nuclear, or thermosensitive) in many crops, including wheat, specifically through altering endogenous ethylene content [39–41]. However, we have no direct data on the role of *Win* in flower development. Therefore, future studies should examine the relationship between ethylene, pistillody, and *Win* genes in detail.

## Conclusion

In the present study, we constructed a high-density linkage map using the F_2_ population of a cross between HTS-1 and CM28TP. Using this linkage map, we mapped *hts* between GBS-SNP markers 4A_109 and 4A_119, separated by a genetic distance of 2.0 cM and a physical distance of 5.2 Mb. Of the 752 protein-coding genes around the two markers, we identified *TaWin1* as the possible candidate gene for *hts*. Real-time PCR supported this conclusion through demonstrating extremely high expression of *TaWin1* gene during the pistil-and-stamen-differentiating stage of HTS-1, as well as preferential expression in PS.Therefore, overexpression of *TaWin1* in the wheat mutant likely results in the transformation of stamens into pistils.

## Additional file

Additional file 1: **Figure S1.** Distribution map of linkage groups. x-axis, chromosome number; y-axis, genetic distance (in cM); and blue, bin marker. **Figure S2.** The genetic linkage map and physical map. The genetic map is shown in red, while the physical map is shown in blue, and the green line indicated the position of each marker on the genetic map and the physical map. **Table S1.** GBS-SNP markers and their BLAST hit information. **Table S2.** The phenotypic of the F2 individuals in HTS-1 × CM28TP. **Table S3.** Genes located in the intervals of hts. (DOCX 623 kb)

## Abbreviations

CM28TP: Chuanmai28 three pistils
CS: Chinese Spring
CSTP: Chinese Spring three pistils
DEGs: Differentially expressed genes (DEGs)
GBS: Genotyping-by-sequencing
HTS-1: Homologous transformation sterility-1
MAS: Marker-assisted selection
NT: Nulli-tetrasomic
P: Pistils
PS: Pistillody stamens
S: Stamens
TP: Three-pistil
