# Supplementary material for: High-density genetic map construction and mapping of the homologous transformation sterility gene (hts) in wheat using GBS markers
Source: BMC Plant Biol. 2018 Nov 26;18:301. doi: 10.1186/s12870-018-1532-x (PMC6258151; doi:10.1186/s12870-018-1532-x)
Supplement: Supplementary file 1 — Figure S1. Distribution map of linkage groups. x-axis, chromosome number; y-axis, genetic distance (in cM); and blue, bin marker. Figure S2. The genetic linkage map and physical map. The genetic map is shown in red, while the physical map is shown in blue, and the green line indicated the position of each marker on the genetic map and the physical map. Table S1. GBS-SNP markers and their BLAST hit information. Table S2. The phenotypic of the F2 individuals in HTS-1 × CM28TP. Table S3. Genes located in the intervals of hts. (DOCX 623 kb) [file 12870_2018_1532_MOESM1_ESM.docx]

Additional file

**High-density Genetic Map Construction and Mapping of the Pistillody Gene (hts) in Wheat Using GBS Markers**

**Qian Yang^1^, Zaijun Yang^1*^, Haifeng Tang^1^, Yan Yu^1^, Zhenyong Chen^1^, Shuhong Wei^1^, Qinxu Sun^1^ and Zhengsong Peng^2*^**

*****Corresponding Author: [yangzaijun1@126.com](mailto:yangzaijun1@126.com); [pzs8833@163.com](mailto:pzs8833@163.com)


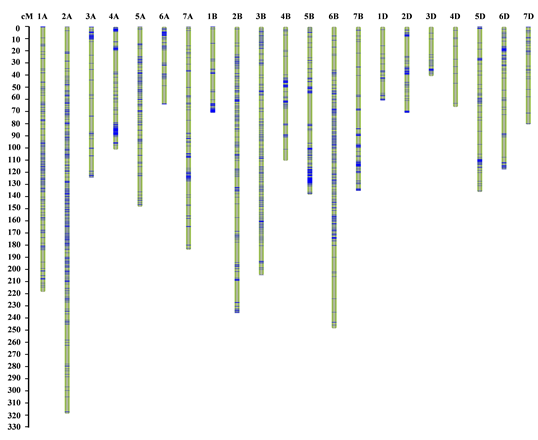


**Figure S1.** Distribution map of linkage groups. x-axis, chromosome number; y-axis, genetic distance (in cM); and blue, bin marker.

**
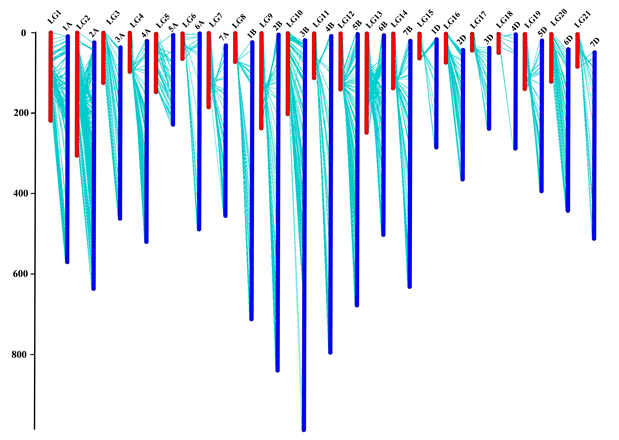
**

**Figure S2.** The genetic linkage map and physical map. The genetic map is shown in red, while the physical map is shown in blue, and the green line indicated the position of each marker on the genetic map and the physical map.

**Table S1.** GBS-SNP markers and their BLAST hit information

| Linkage | Marker | Genetic Distance (cM) | Chromosome | Position |
| --- | --- | --- | --- | --- |
| lg01 | 1A_14 | 0 | 1A | 3617335 |
| lg01 | 1A_19 | 0 | 1A | 3617404 |
| lg01 | 1A_16 | 0 | 1A | 3617391 |
| lg01 | 1A_20 | 0.065 | 1A | 3617412 |
| lg01 | 1A_21 | 0.155 | 1A | 3617418 |
| lg01 | 1A_15 | 0.25 | 1A | 3617380 |
| lg01 | 1A_18 | 0.25 | 1A | 3617399 |
| lg01 | 1A_12 | 0.25 | 1A | 3617311 |
| lg01 | 1A_13 | 0.25 | 1A | 3617317 |
| lg01 | 1A_17 | 0.25 | 1A | 3617397 |
| lg01 | 1A_22 | 0.255 | 1A | 3617428 |
| lg01 | 1A_222 | 9.197 | IWGSC_CSS_1AS_scaff_3257185 | 1530 |
| lg01 | 1A_10 | 14.257 | IWGSC_CSS_1AS_scaff_3288498 | 148 |
| lg01 | 1A_23 | 14.979 | 1A | 3756780 |
| lg01 | 1A_9 | 15.816 | IWGSC_CSS_1AS_scaff_3288498 | 122 |
| lg01 | 1A_11 | 15.816 | IWGSC_CSS_1AS_scaff_3288498 | 159 |
| lg01 | 1A_223 | 20.466 | IWGSC_CSS_1AS_scaff_3257185 | 1546 |
| lg01 | 1A_6 | 22.813 | IWGSC_CSS_1AS_scaff_3295715 | 1821 |
| lg01 | 1A_28 | 26.157 | 1A | 5430026 |
| lg01 | 1A_26 | 26.157 | 1A | 5429901 |
| lg01 | 1A_25 | 26.157 | 1A | 5429881 |
| lg01 | 1A_24 | 26.157 | 1A | 5429837 |
| lg01 | 1A_27 | 26.157 | 1A | 5429910 |
| lg01 | 1A_30 | 34.569 | 1A | 8733379 |
| lg01 | 1A_29 | 35.715 | 1A | 8079800 |
| lg01 | 1A_31 | 37.975 | 1A | 9317199 |
| lg01 | 1A_32 | 37.975 | 1A | 9317201 |
| lg01 | 1A_42 | 45.513 | 1A | 16383499 |
| lg01 | 1A_44 | 45.536 | 1A | 16383639 |
| lg01 | 1A_43 | 45.95 | 1A | 16383598 |
| lg01 | 1A_35 | 49.541 | 1A | 12347237 |
| lg01 | 1A_39 | 50.689 | 1A | 13982810 |
| lg01 | 1A_36 | 50.697 | 1A | 12758487 |
| lg01 | 1A_34 | 51.452 | 1A | 11801452 |
| lg01 | 1A_33 | 51.452 | 1A | 11801403 |
| lg01 | 1A_40 | 52.775 | 1A | 15039360 |
| lg01 | 1A_37 | 54.46 | 1A | 13419762 |
| lg01 | 1A_38 | 56.553 | 1A | 13419776 |
| lg01 | 1A_55 | 62.131 | 1A | 18932397 |
| lg01 | 1A_52 | 63.097 | 1A | 18408633 |
| lg01 | 1A_53 | 63.1 | 1A | 18408649 |
| lg01 | 1A_50 | 63.858 | 1A | 17989552 |
| lg01 | 1A_46 | 63.878 | 1A | 17989365 |
| lg01 | 1A_51 | 63.893 | 1A | 17989594 |
| lg01 | 1A_45 | 63.901 | 1A | 17989364 |
| lg01 | 1A_61 | 64.068 | 1A | 20425933 |
| lg01 | 1A_62 | 64.101 | 1A | 20425959 |
| lg01 | 1A_41 | 65.002 | 1A | 15942302 |
| lg01 | 1A_58 | 66.527 | 1A | 20135004 |
| lg01 | 1A_59 | 66.532 | 1A | 20135039 |
| lg01 | 1A_60 | 66.532 | 1A | 20135040 |
| lg01 | 1A_54 | 68.22 | 1A | 18715936 |
| lg01 | 1A_63 | 69.476 | 1A | 20425992 |
| lg01 | 1A_48 | 69.91 | 1A | 17989448 |
| lg01 | 1A_49 | 69.933 | 1A | 17989464 |
| lg01 | 1A_47 | 69.94 | 1A | 17989423 |
| lg01 | 1A_56 | 71.25 | 1A | 19088890 |
| lg01 | 1A_158 | 73.646 | 1A | 128218456 |
| lg01 | 1A_149 | 76.499 | 1A | 96925417 |
| lg01 | 1A_111 | 76.714 | 1A | 69860652 |
| lg01 | 1A_88 | 77.201 | 1A | 50924430 |
| lg01 | 1A_139 | 77.279 | 1A | 85412012 |
| lg01 | 1A_153 | 77.703 | 1A | 100805473 |
| lg01 | 1A_106 | 80.229 | 1A | 63879612 |
| lg01 | 1A_102 | 84.173 | 1A | 57390992 |
| lg01 | 1A_103 | 84.223 | 1A | 57391081 |
| lg01 | 1A_224 | 89.302 | IWGSC_CSS_1AS_scaff_3280502 | 2441 |
| lg01 | 1A_145 | 93.7 | 1A | 90537835 |
| lg01 | 1A_94 | 94.912 | 1A | 52132183 |
| lg01 | 1A_95 | 94.947 | 1A | 52132318 |
| lg01 | 1A_70 | 95.7 | 1A | 25585783 |
| lg01 | 1A_107 | 96.009 | 1A | 64445101 |
| lg01 | 1A_69 | 96.274 | 1A | 25585769 |
| lg01 | 1A_66 | 96.274 | 1A | 24599352 |
| lg01 | 1A_72 | 96.274 | 1A | 25585862 |
| lg01 | 1A_71 | 96.274 | 1A | 25585842 |
| lg01 | 1A_68 | 96.274 | 1A | 25585739 |
| lg01 | 1A_65 | 96.412 | 1A | 24599344 |
| lg01 | 1A_135 | 97.287 | 1A | 83485949 |
| lg01 | 1A_134 | 97.287 | 1A | 83485933 |
| lg01 | 1A_75 | 98.137 | 1A | 33811623 |
| lg01 | 1A_74 | 98.335 | 1A | 33811575 |
| lg01 | 1A_119 | 99.131 | 1A | 75569149 |
| lg01 | 1A_120 | 99.143 | 1A | 75569245 |
| lg01 | 1A_137 | 100.315 | 1A | 84348175 |
| lg01 | 1A_136 | 100.315 | 1A | 84348170 |
| lg01 | 1A_83 | 101.506 | 1A | 46225905 |
| lg01 | 1A_1 | 103.045 | IWGSC_CSS_1AL_scaff_3903790 | 5295 |
| lg01 | 1A_140 | 103.605 | 1A | 85818010 |
| lg01 | 1A_143 | 104.547 | 1A | 86278149 |
| lg01 | 1A_122 | 104.582 | 1A | 76724921 |
| lg01 | 1A_114 | 104.837 | 1A | 72623430 |
| lg01 | 1A_115 | 104.88 | 1A | 72623471 |
| lg01 | 1A_129 | 104.89 | 1A | 81634817 |
| lg01 | 1A_177 | 105.491 | 1A | 207787840 |
| lg01 | 1A_147 | 106.333 | 1A | 95497183 |
| lg01 | 1A_133 | 107.455 | 1A | 83260356 |
| lg01 | 1A_87 | 108.041 | 1A | 50924407 |
| lg01 | 1A_125 | 108.562 | 1A | 78416643 |
| lg01 | 1A_93 | 109.008 | 1A | 51879867 |
| lg01 | 1A_82 | 109.749 | 1A | 45088941 |
| lg01 | 1A_108 | 110.861 | 1A | 65930526 |
| lg01 | 1A_97 | 111.429 | 1A | 55126943 |
| lg01 | 1A_90 | 111.715 | 1A | 50924528 |
| lg01 | 1A_80 | 112.845 | 1A | 41212010 |
| lg01 | 1A_155 | 113.227 | 1A | 113146363 |
| lg01 | 1A_154 | 113.227 | 1A | 113146290 |
| lg01 | 1A_81 | 113.772 | 1A | 41212182 |
| lg01 | 1A_121 | 114.637 | 1A | 76162282 |
| lg01 | 1A_73 | 115.029 | 1A | 33811516 |
| lg01 | 1A_130 | 115.428 | 1A | 83260211 |
| lg01 | 1A_151 | 115.5 | 1A | 97487383 |
| lg01 | 1A_132 | 115.668 | 1A | 83260302 |
| lg01 | 1A_152 | 116.592 | 1A | 97775005 |
| lg01 | 1A_126 | 117.019 | 1A | 78947918 |
| lg01 | 1A_8 | 117.617 | IWGSC_CSS_1AL_scaff_3960250 | 531 |
| lg01 | 1A_7 | 117.637 | IWGSC_CSS_1AL_scaff_3960250 | 491 |
| lg01 | 1A_101 | 118.157 | 1A | 57167111 |
| lg01 | 1A_112 | 118.343 | 1A | 72095689 |
| lg01 | 1A_100 | 118.853 | 1A | 56812415 |
| lg01 | 1A_161 | 119.718 | 1A | 161094017 |
| lg01 | 1A_162 | 120.344 | 1A | 165079772 |
| lg01 | 1A_84 | 121.199 | 1A | 48926528 |
| lg01 | 1A_78 | 122.745 | 1A | 40269987 |
| lg01 | 1A_79 | 122.75 | 1A | 40270155 |
| lg01 | 1A_156 | 124.229 | 1A | 120085984 |
| lg01 | 1A_144 | 124.802 | 1A | 89845316 |
| lg01 | 1A_148 | 125.368 | 1A | 96925353 |
| lg01 | 1A_110 | 125.483 | 1A | 69400129 |
| lg01 | 1A_150 | 125.623 | 1A | 96925432 |
| lg01 | 1A_113 | 126.134 | 1A | 72095716 |
| lg01 | 1A_92 | 126.55 | 1A | 51745190 |
| lg01 | 1A_77 | 127.665 | 1A | 40260582 |
| lg01 | 1A_138 | 128.029 | 1A | 85363043 |
| lg01 | 1A_131 | 128.074 | 1A | 83260216 |
| lg01 | 1A_67 | 128.954 | 1A | 24599527 |
| lg01 | 1A_98 | 129.543 | 1A | 55991935 |
| lg01 | 1A_64 | 130.015 | 1A | 24599334 |
| lg01 | 1A_96 | 130.725 | 1A | 54615364 |
| lg01 | 1A_146 | 132.183 | 1A | 94058381 |
| lg01 | 1A_4 | 133.087 | IWGSC_CSS_1AL_scaff_3870826 | 7573 |
| lg01 | 1A_3 | 133.087 | IWGSC_CSS_1AL_scaff_3870826 | 7549 |
| lg01 | 1A_5 | 133.087 | IWGSC_CSS_1AL_scaff_3870826 | 7590 |
| lg01 | 1A_105 | 133.463 | 1A | 62293505 |
| lg01 | 1A_109 | 133.839 | 1A | 67418852 |
| lg01 | 1A_116 | 133.902 | 1A | 74985388 |
| lg01 | 1A_85 | 135.199 | 1A | 49658235 |
| lg01 | 1A_117 | 135.369 | 1A | 75143134 |
| lg01 | 1A_118 | 135.369 | 1A | 75143140 |
| lg01 | 1A_91 | 136.117 | 1A | 50924592 |
| lg01 | 1A_89 | 136.117 | 1A | 50924452 |
| lg01 | 1A_86 | 136.117 | 1A | 50924385 |
| lg01 | 1A_99 | 136.144 | 1A | 56311718 |
| lg01 | 1A_165 | 140.436 | 1A | 185641825 |
| lg01 | 1A_142 | 142.228 | 1A | 86065513 |
| lg01 | 1A_141 | 142.513 | 1A | 86065448 |
| lg01 | 1A_164 | 143.534 | 1A | 185147828 |
| lg01 | 1A_163 | 143.556 | 1A | 185147770 |
| lg01 | 1A_169 | 145.488 | 1A | 199483359 |
| lg01 | 1A_168 | 147.898 | 1A | 193291680 |
| lg01 | 1A_123 | 149.861 | 1A | 78125530 |
| lg01 | 1A_124 | 150.442 | 1A | 78125543 |
| lg01 | 1A_76 | 153.204 | 1A | 38082083 |
| lg01 | 1A_167 | 155.253 | 1A | 188850190 |
| lg01 | 1A_172 | 157.182 | 1A | 201597378 |
| lg01 | 1A_171 | 157.486 | 1A | 201597363 |
| lg01 | 1A_173 | 157.508 | 1A | 201597399 |
| lg01 | 1A_170 | 157.546 | 1A | 201597317 |
| lg01 | 1A_178 | 158.051 | 1A | 207894397 |
| lg01 | 1A_127 | 159.148 | 1A | 80413950 |
| lg01 | 1A_128 | 159.15 | 1A | 80413951 |
| lg01 | 1A_166 | 163.371 | 1A | 187995016 |
| lg01 | 1A_189 | 167.445 | 1A | 214380916 |
| lg01 | 1A_176 | 167.993 | 1A | 206816300 |
| lg01 | 1A_174 | 168.073 | 1A | 204715509 |
| lg01 | 1A_175 | 168.908 | 1A | 206216219 |
| lg01 | 1A_182 | 169.585 | 1A | 208391411 |
| lg01 | 1A_181 | 169.888 | 1A | 208391399 |
| lg01 | 1A_179 | 173.783 | 1A | 207924105 |
| lg01 | 1A_180 | 173.796 | 1A | 207924107 |
| lg01 | 1A_190 | 178.404 | 1A | 214952027 |
| lg01 | 1A_193 | 180.331 | 1A | 217556778 |
| lg01 | 1A_194 | 180.431 | 1A | 217556842 |
| lg01 | 1A_186 | 181.299 | 1A | 210943173 |
| lg01 | 1A_183 | 181.444 | 1A | 210943060 |
| lg01 | 1A_184 | 181.561 | 1A | 210943093 |
| lg01 | 1A_187 | 181.657 | 1A | 210943214 |
| lg01 | 1A_188 | 181.659 | 1A | 210943255 |
| lg01 | 1A_185 | 181.667 | 1A | 210943116 |
| lg01 | 1A_192 | 182.689 | 1A | 217556655 |
| lg01 | 1A_195 | 183.25 | 1A | 217556883 |
| lg01 | 1A_191 | 183.932 | 1A | 215773457 |
| lg01 | 1A_196 | 194.01 | 1A | 218992777 |
| lg01 | 1A_217 | 199.267 | 1A | 223558414 |
| lg01 | 1A_215 | 201.108 | 1A | 223425947 |
| lg01 | 1A_216 | 201.389 | 1A | 223425948 |
| lg01 | 1A_214 | 201.392 | 1A | 223425825 |
| lg01 | 1A_197 | 205.122 | 1A | 221761034 |
| lg01 | 1A_198 | 205.122 | 1A | 221761040 |
| lg01 | 1A_200 | 205.122 | 1A | 221761188 |
| lg01 | 1A_201 | 205.122 | 1A | 221761231 |
| lg01 | 1A_199 | 205.122 | 1A | 221761086 |
| lg01 | 1A_204 | 207.325 | 1A | 222633618 |
| lg01 | 1A_211 | 207.656 | 1A | 222633828 |
| lg01 | 1A_207 | 207.671 | 1A | 222633666 |
| lg01 | 1A_208 | 207.671 | 1A | 222633667 |
| lg01 | 1A_205 | 207.671 | 1A | 222633646 |
| lg01 | 1A_210 | 207.671 | 1A | 222633775 |
| lg01 | 1A_212 | 207.671 | 1A | 222633829 |
| lg01 | 1A_206 | 207.671 | 1A | 222633661 |
| lg01 | 1A_209 | 207.671 | 1A | 222633745 |
| lg01 | 1A_213 | 207.699 | 1A | 222633845 |
| lg01 | 1A_203 | 208.179 | 1A | 221778898 |
| lg01 | 1A_202 | 208.201 | 1A | 221778792 |
| lg01 | 1A_2 | 211.343 | IWGSC_CSS_1AL_scaff_3905824 | 1795 |
| lg01 | 1A_218 | 213.091 | 1A | 225294077 |
| lg01 | 1A_220 | 214.567 | 1A | 227928180 |
| lg01 | 1A_219 | 218.573 | 1A | 227729095 |
| lg08 | 1B_80 | 0 | 1B | 283298807 |
| lg08 | 1B_79 | 0.016 | 1B | 283298806 |
| lg08 | 1B_81 | 0.028 | 1B | 283298818 |
| lg08 | 1B_78 | 0.042 | 1B | 283298793 |
| lg08 | 1B_82 | 1.817 | 1B | 284257341 |
| lg08 | 1B_75 | 13.634 | 1B | 276684588 |
| lg08 | 1B_76 | 13.72 | 1B | 278672819 |
| lg08 | 1B_77 | 13.739 | 1B | 278672872 |
| lg08 | 1B_72 | 26.476 | 1B | 272098100 |
| lg08 | 1B_73 | 27.303 | 1B | 272098119 |
| lg08 | 1B_74 | 28.349 | 1B | 274547007 |
| lg08 | 1B_69 | 34.572 | 1B | 265009784 |
| lg08 | 1B_70 | 35.256 | 1B | 265009840 |
| lg08 | 1B_71 | 35.528 | 1B | 265009861 |
| lg08 | 1B_65 | 37.256 | 1B | 261923853 |
| lg08 | 1B_61 | 37.898 | 1B | 259387214 |
| lg08 | 1B_68 | 38.18 | 1B | 262004972 |
| lg08 | 1B_66 | 38.2 | 1B | 262004765 |
| lg08 | 1B_67 | 38.2 | 1B | 262004964 |
| lg08 | 1B_62 | 38.614 | 1B | 261694066 |
| lg08 | 1B_63 | 38.636 | 1B | 261694126 |
| lg08 | 1B_64 | 38.643 | 1B | 261694289 |
| lg08 | 1B_1 | 52.227 | IWGSC_CSS_1BL_scaff_3753739 | 899 |
| lg08 | 1B_57 | 53.456 | 1B | 248360066 |
| lg08 | 1B_2 | 53.695 | IWGSC_CSS_1BL_scaff_3920789 | 1522 |
| lg08 | 1B_51 | 60.141 | 1B | 227600156 |
| lg08 | 1B_49 | 63.18 | 1B | 222213069 |
| lg08 | 1B_53 | 63.41 | 1B | 229418811 |
| lg08 | 1B_52 | 63.41 | 1B | 229418765 |
| lg08 | 1B_55 | 63.669 | 1B | 229418868 |
| lg08 | 1B_54 | 63.679 | 1B | 229418849 |
| lg08 | 1B_50 | 63.905 | 1B | 222434659 |
| lg08 | 1B_48 | 64.318 | 1B | 218258735 |
| lg08 | 1B_36 | 64.462 | 1B | 181522555 |
| lg08 | 1B_47 | 64.734 | 1B | 207689305 |
| lg08 | 1B_45 | 64.937 | 1B | 207522430 |
| lg08 | 1B_46 | 65.182 | 1B | 207522437 |
| lg08 | 1B_43 | 65.562 | 1B | 198203089 |
| lg08 | 1B_35 | 66.621 | 1B | 171048269 |
| lg08 | 1B_33 | 66.774 | 1B | 170788668 |
| lg08 | 1B_4 | 67.339 | 1B | 8992129 |
| lg08 | 1B_5 | 67.339 | 1B | 8992133 |
| lg08 | 1B_6 | 67.469 | 1B | 8992136 |
| lg08 | 1B_22 | 67.781 | 1B | 46817588 |
| lg08 | 1B_23 | 68.053 | 1B | 47964167 |
| lg08 | 1B_19 | 68.29 | 1B | 43745605 |
| lg08 | 1B_25 | 68.401 | 1B | 49673851 |
| lg08 | 1B_39 | 68.409 | 1B | 196716106 |
| lg08 | 1B_30 | 68.532 | 1B | 144112617 |
| lg08 | 1B_37 | 68.565 | 1B | 183706491 |
| lg08 | 1B_18 | 68.596 | 1B | 43655028 |
| lg08 | 1B_17 | 68.596 | 1B | 43655021 |
| lg08 | 1B_24 | 68.676 | 1B | 48276229 |
| lg08 | 1B_16 | 68.676 | 1B | 39424967 |
| lg08 | 1B_11 | 68.74 | 1B | 32515322 |
| lg08 | 1B_9 | 68.776 | 1B | 17583243 |
| lg08 | 1B_7 | 68.789 | 1B | 17583234 |
| lg08 | 1B_3 | 68.807 | IWGSC_CSS_1BL_scaff_3916932 | 2949 |
| lg08 | 1B_14 | 68.844 | 1B | 38537313 |
| lg08 | 1B_8 | 68.881 | 1B | 17583236 |
| lg08 | 1B_13 | 68.965 | 1B | 37361861 |
| lg08 | 1B_29 | 69.01 | 1B | 137666771 |
| lg08 | 1B_10 | 69.115 | 1B | 17583299 |
| lg08 | 1B_15 | 69.197 | 1B | 38676203 |
| lg08 | 1B_38 | 69.271 | 1B | 193447088 |
| lg08 | 1B_21 | 69.499 | 1B | 46205104 |
| lg08 | 1B_20 | 69.557 | 1B | 46204997 |
| lg08 | 1B_32 | 69.639 | 1B | 162347890 |
| lg08 | 1B_26 | 69.73 | 1B | 125227530 |
| lg08 | 1B_41 | 69.916 | 1B | 197912094 |
| lg08 | 1B_40 | 69.929 | 1B | 197912062 |
| lg08 | 1B_31 | 70.12 | 1B | 160539748 |
| lg08 | 1B_12 | 70.159 | 1B | 33335143 |
| lg08 | 1B_56 | 70.386 | 1B | 229418922 |
| lg08 | 1B_28 | 70.537 | 1B | 137122348 |
| lg08 | 1B_44 | 70.63 | 1B | 198203134 |
| lg08 | 1B_27 | 70.635 | 1B | 133109336 |
| lg08 | 1B_42 | 70.648 | 1B | 198203069 |
| lg08 | 1B_34 | 70.986 | 1B | 170960985 |
| lg15 | 1D_29 | 0 | 1D | 112784384 |
| lg15 | 1D_28 | 2.45 | 1D | 107245971 |
| lg15 | 1D_35 | 15.692 | IWGSC_CSS_1DL_scaff_2250131 | 1832 |
| lg15 | 1D_27 | 22.478 | 1D | 82861754 |
| lg15 | 1D_25 | 25.474 | 1D | 69412359 |
| lg15 | 1D_24 | 26.331 | 1D | 69283553 |
| lg15 | 1D_22 | 30.659 | 1D | 45567759 |
| lg15 | 1D_21 | 36.123 | 1D | 36905811 |
| lg15 | 1D_23 | 36.606 | 1D | 48284175 |
| lg15 | 1D_41 | 36.954 | IWGSC_CSS_1DL_scaff_2285479 | 5224 |
| lg15 | 1D_40 | 37.483 | IWGSC_CSS_1DS_scaff_1878023 | 1776 |
| lg15 | 1D_34 | 39.939 | IWGSC_CSS_1DS_scaff_1915722 | 2812 |
| lg15 | 1D_33 | 42.038 | IWGSC_CSS_1DS_scaff_1886262 | 1707 |
| lg15 | 1D_32 | 42.115 | IWGSC_CSS_1DS_scaff_1886262 | 1534 |
| lg15 | 1D_20 | 42.347 | 1D | 36758281 |
| lg15 | 1D_19 | 42.347 | 1D | 36758272 |
| lg15 | 1D_18 | 42.357 | 1D | 36758127 |
| lg15 | 1D_37 | 42.637 | IWGSC_CSS_1DS_scaff_1882748 | 2113 |
| lg15 | 1D_39 | 43.36 | IWGSC_CSS_1DS_scaff_1886402 | 282 |
| lg15 | 1D_42 | 44.731 | IWGSC_CSS_1DS_scaff_1883381 | 164 |
| lg15 | 1D_7 | 54.865 | 1D | 6868515 |
| lg15 | 1D_16 | 55.943 | 1D | 9012478 |
| lg15 | 1D_13 | 56.639 | 1D | 8214657 |
| lg15 | 1D_10 | 56.893 | 1D | 8214539 |
| lg15 | 1D_15 | 56.893 | 1D | 8214729 |
| lg15 | 1D_14 | 56.913 | 1D | 8214727 |
| lg15 | 1D_11 | 56.917 | 1D | 8214543 |
| lg15 | 1D_12 | 57.013 | 1D | 8214545 |
| lg15 | 1D_17 | 59.247 | 1D | 9548704 |
| lg15 | 1D_2 | 59.74 | 1D | 5473567 |
| lg15 | 1D_8 | 60.109 | 1D | 6913967 |
| lg15 | 1D_6 | 60.333 | 1D | 5603680 |
| lg15 | 1D_4 | 60.366 | 1D | 5603655 |
| lg15 | 1D_5 | 60.367 | 1D | 5603665 |
| lg15 | 1D_9 | 60.542 | 1D | 7228073 |
| lg15 | 1D_3 | 60.788 | 1D | 5480261 |
| lg02 | 2A_37 | 0 | 2A | 9612319 |
| lg02 | 2A_38 | 0.001 | 2A | 9612518 |
| lg02 | 2A_18 | 3.301 | IWGSC_CSS_2AS_scaff_5307584 | 4311 |
| lg02 | 2A_46 | 20.539 | 2A | 16189106 |
| lg02 | 2A_45 | 20.579 | 2A | 16189082 |
| lg02 | 2A_50 | 21.492 | 2A | 17249884 |
| lg02 | 2A_48 | 21.492 | 2A | 17249737 |
| lg02 | 2A_47 | 21.743 | 2A | 16522782 |
| lg02 | 2A_49 | 22.006 | 2A | 17249790 |
| lg02 | 2A_40 | 28.341 | 2A | 14999882 |
| lg02 | 2A_44 | 28.343 | 2A | 14999984 |
| lg02 | 2A_42 | 28.348 | 2A | 14999915 |
| lg02 | 2A_41 | 28.35 | 2A | 14999902 |
| lg02 | 2A_39 | 28.35 | 2A | 14999865 |
| lg02 | 2A_43 | 28.35 | 2A | 14999963 |
| lg02 | 2A_402 | 34.468 | IWGSC_CSS_2AS_scaff_5303955 | 4725 |
| lg02 | 2A_403 | 34.49 | IWGSC_CSS_2AS_scaff_5303955 | 4778 |
| lg02 | 2A_363 | 36.603 | 2A | 208531930 |
| lg02 | 2A_187 | 40.767 | 2A | 114894003 |
| lg02 | 2A_247 | 41.795 | 2A | 144522206 |
| lg02 | 2A_246 | 43.202 | 2A | 143717399 |
| lg02 | 2A_123 | 44.622 | 2A | 69346365 |
| lg02 | 2A_347 | 44.629 | 2A | 197323825 |
| lg02 | 2A_236 | 45.909 | 2A | 137478510 |
| lg02 | 2A_235 | 45.909 | 2A | 137478475 |
| lg02 | 2A_344 | 47.271 | 2A | 197321338 |
| lg02 | 2A_345 | 47.274 | 2A | 197321353 |
| lg02 | 2A_122 | 47.732 | 2A | 69346304 |
| lg02 | 2A_185 | 47.83 | 2A | 112071208 |
| lg02 | 2A_186 | 47.835 | 2A | 112071209 |
| lg02 | 2A_130 | 48.663 | 2A | 71690101 |
| lg02 | 2A_299 | 52.572 | 2A | 169826549 |
| lg02 | 2A_300 | 52.577 | 2A | 169826554 |
| lg02 | 2A_264 | 53.24 | 2A | 153099563 |
| lg02 | 2A_166 | 53.72 | 2A | 95552742 |
| lg02 | 2A_289 | 54.865 | 2A | 163979817 |
| lg02 | 2A_325 | 56.129 | 2A | 188157534 |
| lg02 | 2A_326 | 56.129 | 2A | 188157675 |
| lg02 | 2A_307 | 56.301 | 2A | 174208177 |
| lg02 | 2A_270 | 56.387 | 2A | 154053491 |
| lg02 | 2A_305 | 56.387 | 2A | 174208015 |
| lg02 | 2A_220 | 56.395 | 2A | 130816034 |
| lg02 | 2A_194 | 56.924 | 2A | 118790331 |
| lg02 | 2A_195 | 56.924 | 2A | 118790332 |
| lg02 | 2A_269 | 57.524 | 2A | 154053459 |
| lg02 | 2A_240 | 59.444 | 2A | 142470631 |
| lg02 | 2A_338 | 60.206 | 2A | 190203744 |
| lg02 | 2A_131 | 61.065 | 2A | 72950970 |
| lg02 | 2A_126 | 61.814 | 2A | 70154627 |
| lg02 | 2A_322 | 62.588 | 2A | 187175789 |
| lg02 | 2A_321 | 62.65 | 2A | 187175654 |
| lg02 | 2A_243 | 62.892 | 2A | 143242610 |
| lg02 | 2A_265 | 62.97 | 2A | 153099693 |
| lg02 | 2A_175 | 62.97 | 2A | 106106161 |
| lg02 | 2A_245 | 62.97 | 2A | 143242650 |
| lg02 | 2A_290 | 63.369 | 2A | 164612514 |
| lg02 | 2A_341 | 64.745 | 2A | 194064085 |
| lg02 | 2A_342 | 64.745 | 2A | 194064097 |
| lg02 | 2A_337 | 66.429 | 2A | 189073105 |
| lg02 | 2A_278 | 67.888 | 2A | 159868002 |
| lg02 | 2A_241 | 68.301 | 2A | 143174189 |
| lg02 | 2A_318 | 68.866 | 2A | 186807388 |
| lg02 | 2A_343 | 69.266 | 2A | 195553611 |
| lg02 | 2A_319 | 69.793 | 2A | 186807470 |
| lg02 | 2A_171 | 69.813 | 2A | 101326315 |
| lg02 | 2A_320 | 69.813 | 2A | 186807471 |
| lg02 | 2A_288 | 70.128 | 2A | 163853432 |
| lg02 | 2A_204 | 71.081 | 2A | 121824464 |
| lg02 | 2A_205 | 71.15 | 2A | 121824581 |
| lg02 | 2A_193 | 71.798 | 2A | 117358376 |
| lg02 | 2A_192 | 72.087 | 2A | 117358371 |
| lg02 | 2A_223 | 72.591 | 2A | 131486290 |
| lg02 | 2A_222 | 72.591 | 2A | 131486261 |
| lg02 | 2A_279 | 73.356 | 2A | 159989119 |
| lg02 | 2A_208 | 74.404 | 2A | 126498515 |
| lg02 | 2A_280 | 76.316 | 2A | 160212047 |
| lg02 | 2A_296 | 78.55 | 2A | 168739027 |
| lg02 | 2A_202 | 79.254 | 2A | 121387177 |
| lg02 | 2A_340 | 79.263 | 2A | 193526459 |
| lg02 | 2A_316 | 79.515 | 2A | 185131196 |
| lg02 | 2A_285 | 79.914 | 2A | 162551749 |
| lg02 | 2A_177 | 80.221 | 2A | 107029341 |
| lg02 | 2A_232 | 80.269 | 2A | 137087663 |
| lg02 | 2A_233 | 80.269 | 2A | 137087674 |
| lg02 | 2A_231 | 80.269 | 2A | 137087662 |
| lg02 | 2A_183 | 81.355 | 2A | 111649299 |
| lg02 | 2A_297 | 83.903 | 2A | 169521429 |
| lg02 | 2A_311 | 86.558 | 2A | 180195493 |
| lg02 | 2A_198 | 87.076 | 2A | 119638972 |
| lg02 | 2A_291 | 87.142 | 2A | 166069795 |
| lg02 | 2A_191 | 88.173 | 2A | 117179111 |
| lg02 | 2A_397 | 88.491 | IWGSC_CSS_2AS_scaff_5309099 | 1442 |
| lg02 | 2A_190 | 88.819 | 2A | 117179090 |
| lg02 | 2A_234 | 89.986 | 2A | 137342284 |
| lg02 | 2A_323 | 90.861 | 2A | 187188610 |
| lg02 | 2A_267 | 91.222 | 2A | 153891956 |
| lg02 | 2A_227 | 91.259 | 2A | 134726881 |
| lg02 | 2A_308 | 91.576 | 2A | 174613400 |
| lg02 | 2A_412 | 92.294 | IWGSC_CSS_2AS_scaff_5286208 | 3062 |
| lg02 | 2A_309 | 93.049 | 2A | 174613417 |
| lg02 | 2A_263 | 93.051 | 2A | 152585390 |
| lg02 | 2A_274 | 93.725 | 2A | 158649478 |
| lg02 | 2A_339 | 94.477 | 2A | 191394406 |
| lg02 | 2A_317 | 95.816 | 2A | 185327453 |
| lg02 | 2A_128 | 97.412 | 2A | 71662197 |
| lg02 | 2A_129 | 97.412 | 2A | 71662228 |
| lg02 | 2A_282 | 99.295 | 2A | 160590263 |
| lg02 | 2A_199 | 100.653 | 2A | 119639019 |
| lg02 | 2A_197 | 100.926 | 2A | 119638959 |
| lg02 | 2A_196 | 101.182 | 2A | 119638915 |
| lg02 | 2A_24 | 101.664 | IWGSC_CSS_2AS_scaff_5184277 | 5108 |
| lg02 | 2A_292 | 102.138 | 2A | 166069859 |
| lg02 | 2A_182 | 102.235 | 2A | 110418800 |
| lg02 | 2A_188 | 103.492 | 2A | 115745379 |
| lg02 | 2A_117 | 104.586 | 2A | 66490660 |
| lg02 | 2A_219 | 104.962 | 2A | 129930713 |
| lg02 | 2A_416 | 105.235 | IWGSC_CSS_2AS_scaff_5223347 | 8288 |
| lg02 | 2A_310 | 105.488 | 2A | 180121747 |
| lg02 | 2A_189 | 105.617 | 2A | 116843683 |
| lg02 | 2A_405 | 106.16 | IWGSC_CSS_2AS_scaff_5215770 | 6367 |
| lg02 | 2A_293 | 106.412 | 2A | 166234502 |
| lg02 | 2A_213 | 106.974 | 2A | 128975548 |
| lg02 | 2A_283 | 107.329 | 2A | 162099781 |
| lg02 | 2A_276 | 108.146 | 2A | 159010432 |
| lg02 | 2A_221 | 108.164 | 2A | 131255099 |
| lg02 | 2A_275 | 108.204 | 2A | 159010420 |
| lg02 | 2A_4 | 111.543 | IWGSC_CSS_2AL_scaff_6401185 | 3995 |
| lg02 | 2A_89 | 113.745 | 2A | 40767110 |
| lg02 | 2A_68 | 114.258 | 2A | 22187515 |
| lg02 | 2A_116 | 114.448 | 2A | 65147397 |
| lg02 | 2A_110 | 116.264 | 2A | 63421222 |
| lg02 | 2A_111 | 116.636 | 2A | 63421285 |
| lg02 | 2A_225 | 118.113 | 2A | 133723604 |
| lg02 | 2A_118 | 118.832 | 2A | 66490745 |
| lg02 | 2A_304 | 119.037 | 2A | 171836095 |
| lg02 | 2A_74 | 119.143 | 2A | 28819359 |
| lg02 | 2A_209 | 119.341 | 2A | 128017936 |
| lg02 | 2A_273 | 120.899 | 2A | 158455533 |
| lg02 | 2A_212 | 123.942 | 2A | 128042790 |
| lg02 | 2A_94 | 127.06 | 2A | 44542469 |
| lg02 | 2A_294 | 127.446 | 2A | 166488236 |
| lg02 | 2A_27 | 127.76 | IWGSC_CSS_2AS_scaff_5236771 | 1656 |
| lg02 | 2A_314 | 127.83 | 2A | 183051651 |
| lg02 | 2A_77 | 128.026 | 2A | 30371563 |
| lg02 | 2A_104 | 128.331 | 2A | 59112837 |
| lg02 | 2A_105 | 128.331 | 2A | 59112906 |
| lg02 | 2A_409 | 128.549 | IWGSC_CSS_2AS_scaff_5267135 | 2167 |
| lg02 | 2A_249 | 129.682 | 2A | 145667817 |
| lg02 | 2A_13 | 130.807 | IWGSC_CSS_2AS_scaff_5290009 | 2850 |
| lg02 | 2A_400 | 131.969 | IWGSC_CSS_2AS_scaff_5258248 | 1807 |
| lg02 | 2A_312 | 133.421 | 2A | 180681248 |
| lg02 | 2A_254 | 133.524 | 2A | 149067576 |
| lg02 | 2A_211 | 133.604 | 2A | 128042712 |
| lg02 | 2A_399 | 134.333 | IWGSC_CSS_2AS_scaff_5227787 | 626 |
| lg02 | 2A_210 | 134.718 | 2A | 128042661 |
| lg02 | 2A_420 | 135.28 | IWGSC_CSS_2AL_scaff_6385384 | 1021 |
| lg02 | 2A_172 | 135.448 | 2A | 102281772 |
| lg02 | 2A_228 | 136.108 | 2A | 135383602 |
| lg02 | 2A_207 | 136.311 | 2A | 124567477 |
| lg02 | 2A_60 | 137.112 | 2A | 21518123 |
| lg02 | 2A_332 | 137.201 | 2A | 188209979 |
| lg02 | 2A_59 | 137.449 | 2A | 21517928 |
| lg02 | 2A_134 | 137.704 | 2A | 74523113 |
| lg02 | 2A_107 | 137.833 | 2A | 61970243 |
| lg02 | 2A_26 | 137.962 | IWGSC_CSS_2AS_scaff_5236771 | 1640 |
| lg02 | 2A_255 | 137.989 | 2A | 149205246 |
| lg02 | 2A_401 | 138.509 | IWGSC_CSS_2AS_scaff_5276589 | 12331 |
| lg02 | 2A_252 | 139.503 | 2A | 148474042 |
| lg02 | 2A_253 | 139.533 | 2A | 148474071 |
| lg02 | 2A_86 | 139.962 | 2A | 38883923 |
| lg02 | 2A_417 | 140.222 | IWGSC_CSS_2AS_scaff_5256373 | 2488 |
| lg02 | 2A_206 | 140.317 | 2A | 122398033 |
| lg02 | 2A_176 | 141.456 | 2A | 106731006 |
| lg02 | 2A_127 | 141.621 | 2A | 71371834 |
| lg02 | 2A_260 | 142.397 | 2A | 151397850 |
| lg02 | 2A_216 | 142.498 | 2A | 129511165 |
| lg02 | 2A_214 | 142.498 | 2A | 129511114 |
| lg02 | 2A_259 | 142.617 | 2A | 151397813 |
| lg02 | 2A_218 | 143.09 | 2A | 129741386 |
| lg02 | 2A_272 | 143.503 | 2A | 155859517 |
| lg02 | 2A_271 | 143.579 | 2A | 155859461 |
| lg02 | 2A_284 | 144.39 | 2A | 162478715 |
| lg02 | 2A_226 | 145.138 | 2A | 133927323 |
| lg02 | 2A_334 | 145.769 | 2A | 188210037 |
| lg02 | 2A_298 | 145.906 | 2A | 169658314 |
| lg02 | 2A_237 | 146.019 | 2A | 137738496 |
| lg02 | 2A_242 | 146.263 | 2A | 143242090 |
| lg02 | 2A_313 | 147.285 | 2A | 182644009 |
| lg02 | 2A_335 | 148.461 | 2A | 188210114 |
| lg02 | 2A_238 | 148.843 | 2A | 140268232 |
| lg02 | 2A_67 | 149.554 | 2A | 22187473 |
| lg02 | 2A_70 | 150.442 | 2A | 25341395 |
| lg02 | 2A_71 | 150.656 | 2A | 25341404 |
| lg02 | 2A_159 | 150.824 | 2A | 90409148 |
| lg02 | 2A_29 | 151.133 | IWGSC_CSS_2AL_scaff_6381116 | 2927 |
| lg02 | 2A_141 | 151.931 | 2A | 75366948 |
| lg02 | 2A_324 | 152.432 | 2A | 187744677 |
| lg02 | 2A_138 | 152.437 | 2A | 75350489 |
| lg02 | 2A_137 | 152.442 | 2A | 75350461 |
| lg02 | 2A_51 | 152.621 | 2A | 18568122 |
| lg02 | 2A_154 | 153.611 | 2A | 84385267 |
| lg02 | 2A_12 | 154.698 | IWGSC_CSS_2AL_scaff_6365612 | 213 |
| lg02 | 2A_148 | 155.113 | 2A | 79189934 |
| lg02 | 2A_108 | 155.221 | 2A | 62798570 |
| lg02 | 2A_136 | 155.774 | 2A | 75324986 |
| lg02 | 2A_114 | 157.078 | 2A | 64270792 |
| lg02 | 2A_81 | 157.924 | 2A | 33867276 |
| lg02 | 2A_398 | 158.192 | IWGSC_CSS_2AL_scaff_6322657 | 646 |
| lg02 | 2A_121 | 159.045 | 2A | 67723875 |
| lg02 | 2A_115 | 160.039 | 2A | 64420476 |
| lg02 | 2A_152 | 160.457 | 2A | 80981970 |
| lg02 | 2A_103 | 161.164 | 2A | 58810507 |
| lg02 | 2A_174 | 161.268 | 2A | 104642117 |
| lg02 | 2A_63 | 161.991 | 2A | 21929005 |
| lg02 | 2A_64 | 162.004 | 2A | 21929077 |
| lg02 | 2A_66 | 162.014 | 2A | 21929165 |
| lg02 | 2A_170 | 162.624 | 2A | 100158499 |
| lg02 | 2A_90 | 163.262 | 2A | 41253943 |
| lg02 | 2A_101 | 163.863 | 2A | 56085533 |
| lg02 | 2A_158 | 164.124 | 2A | 90051054 |
| lg02 | 2A_98 | 164.518 | 2A | 44951440 |
| lg02 | 2A_407 | 164.635 | IWGSC_CSS_2AL_scaff_6335142 | 2669 |
| lg02 | 2A_150 | 164.768 | 2A | 79935023 |
| lg02 | 2A_97 | 164.768 | 2A | 44951435 |
| lg02 | 2A_132 | 164.86 | 2A | 73485658 |
| lg02 | 2A_418 | 165.565 | IWGSC_CSS_2AL_scaff_6367328 | 2221 |
| lg02 | 2A_155 | 166.43 | 2A | 85813606 |
| lg02 | 2A_85 | 167.437 | 2A | 37693565 |
| lg02 | 2A_145 | 168.397 | 2A | 77957927 |
| lg02 | 2A_53 | 169.247 | 2A | 19191270 |
| lg02 | 2A_149 | 169.935 | 2A | 79915885 |
| lg02 | 2A_421 | 170.498 | IWGSC_CSS_2AL_scaff_6415087 | 4030 |
| lg02 | 2A_106 | 170.787 | 2A | 60737467 |
| lg02 | 2A_120 | 171.25 | 2A | 66817344 |
| lg02 | 2A_268 | 171.985 | 2A | 153913249 |
| lg02 | 2A_203 | 172.427 | 2A | 121618182 |
| lg02 | 2A_230 | 172.594 | 2A | 136512842 |
| lg02 | 2A_250 | 173.167 | 2A | 145892651 |
| lg02 | 2A_17 | 173.334 | IWGSC_CSS_2AS_scaff_5242490 | 1452 |
| lg02 | 2A_201 | 173.783 | 2A | 120806078 |
| lg02 | 2A_229 | 174.109 | 2A | 136512816 |
| lg02 | 2A_19 | 174.721 | IWGSC_CSS_2AL_scaff_6385048 | 1826 |
| lg02 | 2A_28 | 175.01 | IWGSC_CSS_2AS_scaff_5236771 | 1663 |
| lg02 | 2A_25 | 175.297 | IWGSC_CSS_2AS_scaff_5204218 | 5607 |
| lg02 | 2A_303 | 175.625 | 2A | 170694548 |
| lg02 | 2A_256 | 175.995 | 2A | 149479440 |
| lg02 | 2A_224 | 176.483 | 2A | 132730887 |
| lg02 | 2A_277 | 177.126 | 2A | 159678848 |
| lg02 | 2A_257 | 177.995 | 2A | 150134271 |
| lg02 | 2A_295 | 178.22 | 2A | 168163882 |
| lg02 | 2A_258 | 178.55 | 2A | 150135526 |
| lg02 | 2A_336 | 178.836 | 2A | 188873031 |
| lg02 | 2A_301 | 179.142 | 2A | 169920093 |
| lg02 | 2A_248 | 179.427 | 2A | 145165491 |
| lg02 | 2A_153 | 179.918 | 2A | 82278308 |
| lg02 | 2A_239 | 180.591 | 2A | 140440343 |
| lg02 | 2A_14 | 180.654 | IWGSC_CSS_2AL_scaff_6377534 | 3597 |
| lg02 | 2A_302 | 180.7 | 2A | 169920115 |
| lg02 | 2A_410 | 180.811 | IWGSC_CSS_2AS_scaff_5267135 | 2219 |
| lg02 | 2A_146 | 182.202 | 2A | 78879865 |
| lg02 | 2A_262 | 183.147 | 2A | 151397959 |
| lg02 | 2A_287 | 183.669 | 2A | 163147870 |
| lg02 | 2A_164 | 183.773 | 2A | 92918784 |
| lg02 | 2A_78 | 186.322 | 2A | 31207242 |
| lg02 | 2A_112 | 189.453 | 2A | 63547161 |
| lg02 | 2A_151 | 190.439 | 2A | 80981959 |
| lg02 | 2A_113 | 190.447 | 2A | 64152378 |
| lg02 | 2A_415 | 190.45 | IWGSC_CSS_2AL_scaff_6438778 | 5096 |
| lg02 | 2A_100 | 190.539 | 2A | 50674862 |
| lg02 | 2A_88 | 190.789 | 2A | 40372593 |
| lg02 | 2A_54 | 190.875 | 2A | 19308599 |
| lg02 | 2A_133 | 191.562 | 2A | 74365995 |
| lg02 | 2A_33 | 192.388 | IWGSC_CSS_2AL_scaff_6416528 | 517 |
| lg02 | 2A_167 | 193.172 | 2A | 96972714 |
| lg02 | 2A_80 | 193.422 | 2A | 33689181 |
| lg02 | 2A_55 | 193.481 | 2A | 19432045 |
| lg02 | 2A_102 | 193.484 | 2A | 57338942 |
| lg02 | 2A_119 | 193.986 | 2A | 66527924 |
| lg02 | 2A_173 | 194.515 | 2A | 102736051 |
| lg02 | 2A_162 | 194.957 | 2A | 91069951 |
| lg02 | 2A_79 | 194.994 | 2A | 31583593 |
| lg02 | 2A_161 | 194.994 | 2A | 91069930 |
| lg02 | 2A_163 | 195.035 | 2A | 91070060 |
| lg02 | 2A_91 | 196.173 | 2A | 43523644 |
| lg02 | 2A_52 | 196.817 | 2A | 19191161 |
| lg02 | 2A_414 | 197.336 | IWGSC_CSS_2AL_scaff_6367822 | 5503 |
| lg02 | 2A_84 | 197.672 | 2A | 36489764 |
| lg02 | 2A_83 | 198.14 | 2A | 36489698 |
| lg02 | 2A_82 | 198.592 | 2A | 36489675 |
| lg02 | 2A_87 | 200.599 | 2A | 40015917 |
| lg02 | 2A_61 | 201.248 | 2A | 21842132 |
| lg02 | 2A_165 | 201.539 | 2A | 94656651 |
| lg02 | 2A_72 | 201.584 | 2A | 25623355 |
| lg02 | 2A_169 | 201.918 | 2A | 99585531 |
| lg02 | 2A_62 | 202.019 | 2A | 21900202 |
| lg02 | 2A_69 | 202.701 | 2A | 24174366 |
| lg02 | 2A_147 | 203.277 | 2A | 78889234 |
| lg02 | 2A_168 | 203.831 | 2A | 99149517 |
| lg02 | 2A_15 | 204.54 | IWGSC_CSS_2AL_scaff_6352655 | 1956 |
| lg02 | 2A_362 | 206.054 | 2A | 207709544 |
| lg02 | 2A_93 | 207.208 | 2A | 44050744 |
| lg02 | 2A_92 | 207.341 | 2A | 44050722 |
| lg02 | 2A_76 | 208.67 | 2A | 29025240 |
| lg02 | 2A_348 | 208.679 | 2A | 198285411 |
| lg02 | 2A_96 | 208.69 | 2A | 44781674 |
| lg02 | 2A_95 | 208.695 | 2A | 44781568 |
| lg02 | 2A_361 | 209.35 | 2A | 204824675 |
| lg02 | 2A_351 | 209.688 | 2A | 201298550 |
| lg02 | 2A_143 | 209.688 | 2A | 77878062 |
| lg02 | 2A_144 | 209.688 | 2A | 77878112 |
| lg02 | 2A_157 | 209.882 | 2A | 88938764 |
| lg02 | 2A_75 | 210.047 | 2A | 29025117 |
| lg02 | 2A_142 | 210.552 | 2A | 76905328 |
| lg02 | 2A_352 | 212.047 | 2A | 202001302 |
| lg02 | 2A_349 | 215.43 | 2A | 198329976 |
| lg02 | 2A_156 | 215.869 | 2A | 87668041 |
| lg02 | 2A_353 | 217.126 | 2A | 202177286 |
| lg02 | 2A_413 | 217.829 | IWGSC_CSS_2AL_scaff_6421524 | 9123 |
| lg02 | 2A_16 | 218.977 | IWGSC_CSS_2AL_scaff_6438878 | 5840 |
| lg02 | 2A_5 | 220.07 | IWGSC_CSS_2AL_scaff_6376894 | 3337 |
| lg02 | 2A_73 | 222.188 | 2A | 28805753 |
| lg02 | 2A_371 | 223.212 | 2A | 218284534 |
| lg02 | 2A_355 | 224.006 | 2A | 203870398 |
| lg02 | 2A_356 | 224.006 | 2A | 203870406 |
| lg02 | 2A_357 | 224.006 | 2A | 203870425 |
| lg02 | 2A_358 | 224.006 | 2A | 203870492 |
| lg02 | 2A_354 | 224.006 | 2A | 203870378 |
| lg02 | 2A_365 | 224.1 | 2A | 209527207 |
| lg02 | 2A_368 | 224.76 | 2A | 213938103 |
| lg02 | 2A_369 | 224.811 | 2A | 213938181 |
| lg02 | 2A_367 | 224.854 | 2A | 213938052 |
| lg02 | 2A_364 | 225.673 | 2A | 209527121 |
| lg02 | 2A_135 | 226.205 | 2A | 75264266 |
| lg02 | 2A_34 | 227.26 | IWGSC_CSS_2AL_scaff_4294931 | 779 |
| lg02 | 2A_372 | 232.703 | 2A | 223201604 |
| lg02 | 2A_373 | 234.196 | 2A | 225399066 |
| lg02 | 2A_20 | 234.656 | IWGSC_CSS_2AL_scaff_6356128 | 1131 |
| lg02 | 2A_21 | 234.656 | IWGSC_CSS_2AL_scaff_6356128 | 1154 |
| lg02 | 2A_22 | 234.656 | IWGSC_CSS_2AL_scaff_6356128 | 1162 |
| lg02 | 2A_381 | 236.703 | 2A | 231974307 |
| lg02 | 2A_375 | 241.8 | 2A | 230139150 |
| lg02 | 2A_379 | 242.815 | 2A | 230987458 |
| lg02 | 2A_378 | 242.817 | 2A | 230987446 |
| lg02 | 2A_376 | 243.418 | 2A | 230523539 |
| lg02 | 2A_377 | 243.726 | 2A | 230523591 |
| lg02 | 2A_408 | 244.839 | IWGSC_CSS_2AL_scaff_6385015 | 7631 |
| lg02 | 2A_374 | 245.338 | 2A | 228871089 |
| lg02 | 2A_382 | 258.276 | 2A | 235360115 |
| lg02 | 2A_383 | 258.278 | 2A | 235360133 |
| lg02 | 2A_380 | 260.204 | 2A | 231777048 |
| lg02 | 2A_1 | 262.613 | IWGSC_CSS_2AL_scaff_6316287 | 4482 |
| lg02 | 2A_2 | 262.613 | IWGSC_CSS_2AL_scaff_6316287 | 4510 |
| lg02 | 2A_389 | 278.048 | 2A | 243617131 |
| lg02 | 2A_388 | 279.596 | 2A | 243478065 |
| lg02 | 2A_387 | 279.598 | 2A | 243477996 |
| lg02 | 2A_386 | 279.862 | 2A | 243477995 |
| lg02 | 2A_390 | 282.001 | 2A | 244065336 |
| lg02 | 2A_384 | 283.597 | 2A | 242584995 |
| lg02 | 2A_394 | 286.475 | 2A | 254131565 |
| lg02 | 2A_391 | 288.469 | 2A | 245662416 |
| lg02 | 2A_395 | 296.803 | 2A | 254439334 |
| lg02 | 2A_392 | 299.83 | 2A | 252904758 |
| lg02 | 2A_393 | 305.062 | 2A | 253862752 |
| lg02 | 2A_7 | 317.378 | IWGSC_CSS_2AL_scaff_6404346 | 3060 |
| lg02 | 2A_8 | 317.38 | IWGSC_CSS_2AL_scaff_6404346 | 3189 |
| lg02 | 2A_9 | 317.387 | IWGSC_CSS_2AL_scaff_6404346 | 3228 |
| lg02 | 2A_6 | 317.389 | IWGSC_CSS_2AL_scaff_6404346 | 3057 |
| lg02 | 2A_11 | 318.951 | IWGSC_CSS_2AL_scaff_6366021 | 2680 |
| lg09 | 2B_258 | 0 | 2B | 335122001 |
| lg09 | 2B_256 | 1.909 | 2B | 334231155 |
| lg09 | 2B_257 | 1.911 | 2B | 334231240 |
| lg09 | 2B_255 | 13.479 | 2B | 331121178 |
| lg09 | 2B_250 | 15.905 | 2B | 330967418 |
| lg09 | 2B_249 | 15.905 | 2B | 330967380 |
| lg09 | 2B_245 | 15.905 | 2B | 330967327 |
| lg09 | 2B_247 | 15.905 | 2B | 330967332 |
| lg09 | 2B_252 | 15.905 | 2B | 330967478 |
| lg09 | 2B_251 | 15.905 | 2B | 330967435 |
| lg09 | 2B_254 | 15.905 | 2B | 330967535 |
| lg09 | 2B_246 | 15.905 | 2B | 330967331 |
| lg09 | 2B_248 | 15.905 | 2B | 330967373 |
| lg09 | 2B_253 | 15.905 | 2B | 330967495 |
| lg09 | 2B_40 | 15.905 | IWGSC_CSS_2BL_scaff_8041823 | 2830 |
| lg09 | 2B_244 | 15.905 | 2B | 330967317 |
| lg09 | 2B_217 | 22.868 | 2B | 307449782 |
| lg09 | 2B_216 | 22.868 | 2B | 307449763 |
| lg09 | 2B_218 | 24.036 | 2B | 308133974 |
| lg09 | 2B_240 | 24.889 | 2B | 321791746 |
| lg09 | 2B_238 | 24.889 | 2B | 321791739 |
| lg09 | 2B_239 | 25.139 | 2B | 321791740 |
| lg09 | 2B_237 | 25.39 | 2B | 321791730 |
| lg09 | 2B_215 | 25.993 | 2B | 306634232 |
| lg09 | 2B_242 | 27.236 | 2B | 325863546 |
| lg09 | 2B_241 | 27.241 | 2B | 325863537 |
| lg09 | 2B_243 | 27.241 | 2B | 325863559 |
| lg09 | 2B_2 | 28.086 | IWGSC_CSS_2BL_scaff_8046835 | 3359 |
| lg09 | 2B_212 | 28.566 | 2B | 306162455 |
| lg09 | 2B_236 | 28.929 | 2B | 321791722 |
| lg09 | 2B_232 | 28.947 | 2B | 316893245 |
| lg09 | 2B_235 | 28.954 | 2B | 316893366 |
| lg09 | 2B_234 | 28.962 | 2B | 316893350 |
| lg09 | 2B_233 | 28.972 | 2B | 316893347 |
| lg09 | 2B_230 | 29.852 | 2B | 314372085 |
| lg09 | 2B_228 | 29.867 | 2B | 314371949 |
| lg09 | 2B_229 | 29.885 | 2B | 314372017 |
| lg09 | 2B_209 | 30.44 | 2B | 305737796 |
| lg09 | 2B_211 | 30.44 | 2B | 305737827 |
| lg09 | 2B_210 | 30.44 | 2B | 305737800 |
| lg09 | 2B_220 | 31.45 | 2B | 310046851 |
| lg09 | 2B_219 | 31.45 | 2B | 310046837 |
| lg09 | 2B_204 | 32.31 | 2B | 305293835 |
| lg09 | 2B_205 | 32.315 | 2B | 305293919 |
| lg09 | 2B_203 | 32.4 | 2B | 305293824 |
| lg09 | 2B_208 | 33.63 | 2B | 305470009 |
| lg09 | 2B_207 | 33.668 | 2B | 305469950 |
| lg09 | 2B_206 | 34.5 | 2B | 305293927 |
| lg09 | 2B_231 | 34.515 | 2B | 314979299 |
| lg09 | 2B_213 | 34.517 | 2B | 306609976 |
| lg09 | 2B_214 | 34.517 | 2B | 306609993 |
| lg09 | 2B_227 | 38.651 | 2B | 311697372 |
| lg09 | 2B_226 | 38.651 | 2B | 311697343 |
| lg09 | 2B_224 | 38.653 | 2B | 311697317 |
| lg09 | 2B_223 | 38.656 | 2B | 311697316 |
| lg09 | 2B_222 | 38.661 | 2B | 311697310 |
| lg09 | 2B_225 | 38.666 | 2B | 311697325 |
| lg09 | 2B_221 | 38.668 | 2B | 311697298 |
| lg09 | 2B_192 | 40.37 | 2B | 285793624 |
| lg09 | 2B_200 | 41.118 | 2B | 298665769 |
| lg09 | 2B_260 | 41.492 | IWGSC_CSS_2BL_scaff_8092794 | 1009 |
| lg09 | 2B_272 | 42.706 | IWGSC_CSS_2BL_scaff_8073617 | 1353 |
| lg09 | 2B_271 | 42.706 | IWGSC_CSS_2BL_scaff_8073617 | 1331 |
| lg09 | 2B_202 | 44.599 | 2B | 300517325 |
| lg09 | 2B_188 | 46.807 | 2B | 282746742 |
| lg09 | 2B_197 | 48.081 | 2B | 297937102 |
| lg09 | 2B_15 | 49.096 | IWGSC_CSS_2BL_scaff_7999930 | 10578 |
| lg09 | 2B_20 | 49.096 | IWGSC_CSS_2BL_scaff_7999930 | 10674 |
| lg09 | 2B_14 | 49.096 | IWGSC_CSS_2BL_scaff_7999930 | 10563 |
| lg09 | 2B_18 | 49.096 | IWGSC_CSS_2BL_scaff_7999930 | 10627 |
| lg09 | 2B_12 | 49.096 | IWGSC_CSS_2BL_scaff_7999930 | 10554 |
| lg09 | 2B_19 | 49.096 | IWGSC_CSS_2BL_scaff_7999930 | 10644 |
| lg09 | 2B_11 | 49.096 | IWGSC_CSS_2BL_scaff_7999930 | 10533 |
| lg09 | 2B_10 | 49.096 | IWGSC_CSS_2BL_scaff_7999930 | 10503 |
| lg09 | 2B_13 | 49.096 | IWGSC_CSS_2BL_scaff_7999930 | 10557 |
| lg09 | 2B_9 | 49.096 | IWGSC_CSS_2BL_scaff_7999930 | 10500 |
| lg09 | 2B_17 | 49.096 | IWGSC_CSS_2BL_scaff_7999930 | 10592 |
| lg09 | 2B_7 | 49.163 | IWGSC_CSS_2BL_scaff_7999930 | 10484 |
| lg09 | 2B_8 | 49.346 | IWGSC_CSS_2BL_scaff_7999930 | 10495 |
| lg09 | 2B_16 | 49.346 | IWGSC_CSS_2BL_scaff_7999930 | 10589 |
| lg09 | 2B_194 | 50.612 | 2B | 288406412 |
| lg09 | 2B_195 | 50.612 | 2B | 288406423 |
| lg09 | 2B_196 | 50.612 | 2B | 288406430 |
| lg09 | 2B_277 | 52.56 | IWGSC_CSS_2BL_scaff_8028022 | 178 |
| lg09 | 2B_278 | 52.56 | IWGSC_CSS_2BL_scaff_8028022 | 179 |
| lg09 | 2B_36 | 52.896 | IWGSC_CSS_2BL_scaff_8037985 | 3052 |
| lg09 | 2B_191 | 53.751 | 2B | 285793605 |
| lg09 | 2B_193 | 53.766 | 2B | 285793670 |
| lg09 | 2B_190 | 53.791 | 2B | 285793546 |
| lg09 | 2B_189 | 53.803 | 2B | 285793517 |
| lg09 | 2B_198 | 56.743 | 2B | 298468951 |
| lg09 | 2B_199 | 56.743 | 2B | 298468965 |
| lg09 | 2B_201 | 57.914 | 2B | 298879038 |
| lg09 | 2B_168 | 58.006 | 2B | 270156386 |
| lg09 | 2B_179 | 58.009 | 2B | 274612082 |
| lg09 | 2B_187 | 58.823 | 2B | 278268192 |
| lg09 | 2B_185 | 59.741 | 2B | 274612192 |
| lg09 | 2B_174 | 59.947 | 2B | 270610797 |
| lg09 | 2B_177 | 60.167 | 2B | 272861442 |
| lg09 | 2B_170 | 60.431 | 2B | 270610628 |
| lg09 | 2B_173 | 60.591 | 2B | 270610714 |
| lg09 | 2B_183 | 60.844 | 2B | 274612138 |
| lg09 | 2B_184 | 61.017 | 2B | 274612184 |
| lg09 | 2B_186 | 61.18 | 2B | 274612328 |
| lg09 | 2B_182 | 61.312 | 2B | 274612110 |
| lg09 | 2B_172 | 61.445 | 2B | 270610699 |
| lg09 | 2B_171 | 61.447 | 2B | 270610689 |
| lg09 | 2B_180 | 61.492 | 2B | 274612091 |
| lg09 | 2B_169 | 61.567 | 2B | 270156412 |
| lg09 | 2B_181 | 61.567 | 2B | 274612099 |
| lg09 | 2B_175 | 61.6 | 2B | 272090435 |
| lg09 | 2B_34 | 63.27 | IWGSC_CSS_2BL_scaff_8089235 | 2802 |
| lg09 | 2B_35 | 63.325 | IWGSC_CSS_2BL_scaff_8089235 | 2906 |
| lg09 | 2B_160 | 65.125 | 2B | 260382717 |
| lg09 | 2B_22 | 66.124 | IWGSC_CSS_2BL_scaff_8002816 | 2499 |
| lg09 | 2B_21 | 66.127 | IWGSC_CSS_2BL_scaff_8002816 | 2492 |
| lg09 | 2B_154 | 67.221 | 2B | 254822225 |
| lg09 | 2B_146 | 68.982 | 2B | 231156694 |
| lg09 | 2B_152 | 69.631 | 2B | 246994998 |
| lg09 | 2B_5 | 70.664 | IWGSC_CSS_2BL_scaff_8051324 | 3168 |
| lg09 | 2B_178 | 72.327 | 2B | 274425125 |
| lg09 | 2B_273 | 73.383 | IWGSC_CSS_2BL_scaff_8030282 | 5060 |
| lg09 | 2B_263 | 73.797 | IWGSC_CSS_2BL_scaff_8008218 | 8496 |
| lg09 | 2B_264 | 73.797 | IWGSC_CSS_2BL_scaff_8008218 | 8588 |
| lg09 | 2B_262 | 73.797 | IWGSC_CSS_2BL_scaff_8008218 | 8453 |
| lg09 | 2B_162 | 74.815 | 2B | 260862102 |
| lg09 | 2B_261 | 75.591 | IWGSC_CSS_2BL_scaff_7980300 | 256 |
| lg09 | 2B_155 | 76.524 | 2B | 256740136 |
| lg09 | 2B_157 | 76.582 | 2B | 256740239 |
| lg09 | 2B_158 | 76.582 | 2B | 256740252 |
| lg09 | 2B_156 | 76.582 | 2B | 256740237 |
| lg09 | 2B_159 | 76.594 | 2B | 256740306 |
| lg09 | 2B_163 | 77.597 | 2B | 261314832 |
| lg09 | 2B_6 | 77.677 | IWGSC_CSS_2BL_scaff_7981268 | 1563 |
| lg09 | 2B_269 | 80.696 | IWGSC_CSS_2BL_scaff_8020438 | 223 |
| lg09 | 2B_144 | 81.358 | 2B | 219444226 |
| lg09 | 2B_161 | 82.16 | 2B | 260577357 |
| lg09 | 2B_164 | 84.115 | 2B | 262886377 |
| lg09 | 2B_165 | 84.135 | 2B | 262886428 |
| lg09 | 2B_166 | 84.245 | 2B | 262886501 |
| lg09 | 2B_167 | 84.46 | 2B | 266248373 |
| lg09 | 2B_176 | 85.958 | 2B | 272621106 |
| lg09 | 2B_150 | 90.96 | 2B | 246287158 |
| lg09 | 2B_127 | 95.107 | 2B | 141322725 |
| lg09 | 2B_126 | 95.135 | 2B | 141322698 |
| lg09 | 2B_130 | 96.183 | 2B | 165342295 |
| lg09 | 2B_149 | 97.119 | 2B | 244448878 |
| lg09 | 2B_148 | 97.137 | 2B | 244448835 |
| lg09 | 2B_147 | 97.142 | 2B | 244448781 |
| lg09 | 2B_129 | 98.085 | 2B | 165342283 |
| lg09 | 2B_1 | 98.293 | IWGSC_CSS_2BL_scaff_8051383 | 2903 |
| lg09 | 2B_145 | 99.349 | 2B | 223747940 |
| lg09 | 2B_143 | 102.646 | 2B | 216375279 |
| lg09 | 2B_151 | 105.008 | 2B | 246628939 |
| lg09 | 2B_138 | 105.39 | 2B | 190500577 |
| lg09 | 2B_139 | 105.653 | 2B | 190500638 |
| lg09 | 2B_137 | 105.921 | 2B | 190500554 |
| lg09 | 2B_140 | 105.921 | 2B | 190500652 |
| lg09 | 2B_136 | 105.921 | 2B | 190500528 |
| lg09 | 2B_141 | 105.921 | 2B | 190500673 |
| lg09 | 2B_118 | 105.924 | 2B | 96082574 |
| lg09 | 2B_276 | 106.623 | IWGSC_CSS_2BL_scaff_8019189 | 1170 |
| lg09 | 2B_134 | 107.687 | 2B | 180626789 |
| lg09 | 2B_132 | 107.687 | 2B | 180626664 |
| lg09 | 2B_135 | 107.687 | 2B | 180626813 |
| lg09 | 2B_131 | 107.687 | 2B | 180626662 |
| lg09 | 2B_133 | 107.687 | 2B | 180626711 |
| lg09 | 2B_142 | 108.679 | 2B | 190502343 |
| lg09 | 2B_121 | 110.329 | 2B | 116703033 |
| lg09 | 2B_124 | 117.126 | 2B | 136712644 |
| lg09 | 2B_128 | 117.535 | 2B | 152090959 |
| lg09 | 2B_125 | 118.042 | 2B | 136712675 |
| lg09 | 2B_113 | 118.722 | 2B | 63721235 |
| lg09 | 2B_114 | 118.729 | 2B | 63721271 |
| lg09 | 2B_122 | 119.684 | 2B | 127317459 |
| lg09 | 2B_123 | 120.625 | 2B | 128329627 |
| lg09 | 2B_116 | 121.732 | 2B | 78441514 |
| lg09 | 2B_117 | 121.732 | 2B | 78441540 |
| lg09 | 2B_3 | 122.793 | IWGSC_CSS_2BS_scaff_5158209 | 2043 |
| lg09 | 2B_4 | 123.526 | IWGSC_CSS_2BS_scaff_5158209 | 2089 |
| lg09 | 2B_105 | 132.336 | 2B | 48467440 |
| lg09 | 2B_96 | 132.356 | 2B | 35710089 |
| lg09 | 2B_98 | 132.356 | 2B | 35710185 |
| lg09 | 2B_97 | 132.359 | 2B | 35710106 |
| lg09 | 2B_94 | 132.359 | 2B | 35710052 |
| lg09 | 2B_93 | 132.609 | 2B | 35710031 |
| lg09 | 2B_95 | 132.86 | 2B | 35710084 |
| lg09 | 2B_112 | 133.211 | 2B | 54755513 |
| lg09 | 2B_108 | 134.106 | 2B | 48967786 |
| lg09 | 2B_106 | 134.119 | 2B | 48967674 |
| lg09 | 2B_111 | 134.119 | 2B | 48967895 |
| lg09 | 2B_107 | 134.119 | 2B | 48967695 |
| lg09 | 2B_109 | 134.119 | 2B | 48967839 |
| lg09 | 2B_110 | 134.119 | 2B | 48967841 |
| lg09 | 2B_102 | 134.37 | 2B | 42446897 |
| lg09 | 2B_103 | 134.872 | 2B | 44748560 |
| lg09 | 2B_104 | 134.872 | 2B | 44748720 |
| lg09 | 2B_100 | 135.243 | 2B | 39878184 |
| lg09 | 2B_101 | 135.253 | 2B | 39878188 |
| lg09 | 2B_115 | 135.984 | 2B | 64759008 |
| lg09 | 2B_92 | 137.675 | 2B | 31666725 |
| lg09 | 2B_99 | 140.545 | 2B | 35753400 |
| lg09 | 2B_91 | 145.95 | 2B | 30560125 |
| lg09 | 2B_270 | 157.27 | IWGSC_CSS_2BS_scaff_5167539 | 10794 |
| lg09 | 2B_89 | 168.646 | 2B | 16731644 |
| lg09 | 2B_88 | 168.649 | 2B | 16731584 |
| lg09 | 2B_90 | 168.654 | 2B | 16731710 |
| lg09 | 2B_87 | 170.817 | 2B | 14536030 |
| lg09 | 2B_80 | 171.703 | 2B | 10990529 |
| lg09 | 2B_85 | 172.132 | 2B | 13487578 |
| lg09 | 2B_86 | 172.134 | 2B | 13487604 |
| lg09 | 2B_83 | 172.964 | 2B | 12810284 |
| lg09 | 2B_82 | 172.964 | 2B | 12810280 |
| lg09 | 2B_79 | 173.679 | 2B | 10725361 |
| lg09 | 2B_78 | 174.539 | 2B | 10510309 |
| lg09 | 2B_84 | 175.608 | 2B | 13345454 |
| lg09 | 2B_81 | 177.597 | 2B | 12736701 |
| lg09 | 2B_70 | 194.325 | 2B | 7856712 |
| lg09 | 2B_72 | 194.34 | 2B | 7856752 |
| lg09 | 2B_75 | 195.989 | 2B | 9299199 |
| lg09 | 2B_73 | 196.313 | 2B | 8055530 |
| lg09 | 2B_65 | 196.611 | 2B | 7533112 |
| lg09 | 2B_67 | 197.296 | 2B | 7854348 |
| lg09 | 2B_68 | 197.296 | 2B | 7854357 |
| lg09 | 2B_66 | 197.566 | 2B | 7854310 |
| lg09 | 2B_74 | 197.842 | 2B | 8055538 |
| lg09 | 2B_69 | 199.368 | 2B | 7856645 |
| lg09 | 2B_71 | 199.388 | 2B | 7856722 |
| lg09 | 2B_77 | 201.763 | 2B | 9580266 |
| lg09 | 2B_76 | 201.776 | 2B | 9580244 |
| lg09 | 2B_56 | 207.13 | 2B | 4581897 |
| lg09 | 2B_63 | 207.998 | 2B | 4720344 |
| lg09 | 2B_59 | 208.236 | 2B | 4720305 |
| lg09 | 2B_60 | 208.426 | 2B | 4720319 |
| lg09 | 2B_57 | 208.541 | 2B | 4720260 |
| lg09 | 2B_64 | 208.641 | 2B | 4720406 |
| lg09 | 2B_58 | 208.779 | 2B | 4720283 |
| lg09 | 2B_61 | 208.879 | 2B | 4720338 |
| lg09 | 2B_62 | 208.984 | 2B | 4720340 |
| lg09 | 2B_55 | 209.295 | 2B | 4581837 |
| lg09 | 2B_38 | 227.1 | IWGSC_CSS_2BS_scaff_5166352 | 1434 |
| lg09 | 2B_37 | 227.183 | IWGSC_CSS_2BS_scaff_5166352 | 1303 |
| lg09 | 2B_39 | 227.233 | IWGSC_CSS_2BS_scaff_5166352 | 1503 |
| lg09 | 2B_266 | 227.456 | IWGSC_CSS_2BS_scaff_5172771 | 933 |
| lg09 | 2B_267 | 227.458 | IWGSC_CSS_2BS_scaff_5172771 | 940 |
| lg09 | 2B_25 | 230.456 | IWGSC_CSS_2BS_scaff_5246058 | 6964 |
| lg09 | 2B_24 | 230.456 | IWGSC_CSS_2BS_scaff_5246058 | 6933 |
| lg09 | 2B_26 | 230.456 | IWGSC_CSS_2BS_scaff_5246058 | 7085 |
| lg09 | 2B_23 | 230.456 | IWGSC_CSS_2BS_scaff_5246058 | 6888 |
| lg09 | 2B_42 | 232.49 | IWGSC_CSS_2BS_scaff_5173734 | 3683 |
| lg09 | 2B_41 | 232.497 | IWGSC_CSS_2BS_scaff_5173734 | 3594 |
| lg09 | 2B_43 | 232.497 | IWGSC_CSS_2BS_scaff_5173734 | 3700 |
| lg09 | 2B_45 | 232.497 | IWGSC_CSS_2BS_scaff_5173734 | 3821 |
| lg09 | 2B_44 | 232.497 | IWGSC_CSS_2BS_scaff_5173734 | 3730 |
| lg09 | 2B_265 | 233.088 | IWGSC_CSS_2BS_scaff_5157815 | 5686 |
| lg09 | 2B_33 | 233.755 | IWGSC_CSS_2BS_scaff_5173679 | 6935 |
| lg09 | 2B_27 | 233.755 | IWGSC_CSS_2BS_scaff_5173679 | 6809 |
| lg09 | 2B_30 | 233.755 | IWGSC_CSS_2BS_scaff_5173679 | 6841 |
| lg09 | 2B_28 | 233.755 | IWGSC_CSS_2BS_scaff_5173679 | 6820 |
| lg09 | 2B_29 | 233.755 | IWGSC_CSS_2BS_scaff_5173679 | 6836 |
| lg09 | 2B_31 | 234.258 | IWGSC_CSS_2BS_scaff_5173679 | 6893 |
| lg09 | 2B_32 | 234.508 | IWGSC_CSS_2BS_scaff_5173679 | 6911 |
| lg09 | 2B_52 | 235.289 | 2B | 1446871 |
| lg09 | 2B_53 | 235.302 | 2B | 1446888 |
| lg09 | 2B_47 | 235.324 | 2B | 1446783 |
| lg09 | 2B_51 | 235.344 | 2B | 1446863 |
| lg09 | 2B_49 | 235.362 | 2B | 1446786 |
| lg09 | 2B_54 | 235.412 | 2B | 1446966 |
| lg09 | 2B_48 | 235.452 | 2B | 1446784 |
| lg09 | 2B_46 | 235.859 | 2B | 1446766 |
| lg09 | 2B_50 | 235.921 | 2B | 1446854 |
| lg16 | 2D_28 | 0 | 2D | 16053619 |
| lg16 | 2D_40 | 2.769 | 2D | 20545670 |
| lg16 | 2D_42 | 2.769 | 2D | 20545714 |
| lg16 | 2D_41 | 2.769 | 2D | 20545682 |
| lg16 | 2D_49 | 4.812 | 2D | 21809541 |
| lg16 | 2D_30 | 5.246 | 2D | 16534281 |
| lg16 | 2D_48 | 5.559 | 2D | 21346707 |
| lg16 | 2D_1 | 6.405 | IWGSC_CSS_2DS_scaff_5333751 | 6118 |
| lg16 | 2D_37 | 6.563 | 2D | 18751646 |
| lg16 | 2D_29 | 6.59 | 2D | 16363511 |
| lg16 | 2D_36 | 6.69 | 2D | 17937032 |
| lg16 | 2D_31 | 6.722 | 2D | 16938892 |
| lg16 | 2D_39 | 6.776 | 2D | 19490987 |
| lg16 | 2D_125 | 6.794 | IWGSC_CSS_2DS_scaff_5348667 | 737 |
| lg16 | 2D_38 | 6.836 | 2D | 18946547 |
| lg16 | 2D_47 | 6.999 | 2D | 21087778 |
| lg16 | 2D_46 | 7.001 | 2D | 21087775 |
| lg16 | 2D_45 | 7.001 | 2D | 21087767 |
| lg16 | 2D_44 | 7.008 | 2D | 21087707 |
| lg16 | 2D_33 | 7.165 | 2D | 17326150 |
| lg16 | 2D_32 | 7.233 | 2D | 16948326 |
| lg16 | 2D_2 | 7.441 | IWGSC_CSS_2DS_scaff_5368605 | 11567 |
| lg16 | 2D_43 | 7.757 | 2D | 20575650 |
| lg16 | 2D_35 | 8.14 | 2D | 17659271 |
| lg16 | 2D_34 | 8.142 | 2D | 17659261 |
| lg16 | 2D_61 | 24.387 | 2D | 55248021 |
| lg16 | 2D_128 | 24.837 | IWGSC_CSS_2DS_scaff_5315993 | 840 |
| lg16 | 2D_58 | 25.551 | 2D | 51171046 |
| lg16 | 2D_55 | 27.73 | 2D | 44371074 |
| lg16 | 2D_62 | 28.769 | 2D | 57395800 |
| lg16 | 2D_57 | 30.165 | 2D | 48659193 |
| lg16 | 2D_74 | 32.964 | 2D | 88320719 |
| lg16 | 2D_86 | 33.574 | 2D | 110699296 |
| lg16 | 2D_73 | 33.786 | 2D | 82427676 |
| lg16 | 2D_75 | 34.343 | 2D | 92243441 |
| lg16 | 2D_69 | 34.401 | 2D | 75228895 |
| lg16 | 2D_68 | 34.478 | 2D | 73464899 |
| lg16 | 2D_70 | 34.497 | 2D | 75228915 |
| lg16 | 2D_77 | 34.615 | 2D | 93186683 |
| lg16 | 2D_67 | 34.97 | 2D | 72867645 |
| lg16 | 2D_72 | 35.292 | 2D | 79031484 |
| lg16 | 2D_59 | 36.26 | 2D | 51315229 |
| lg16 | 2D_54 | 36.274 | 2D | 43272996 |
| lg16 | 2D_60 | 36.486 | 2D | 53932269 |
| lg16 | 2D_84 | 36.659 | 2D | 106258993 |
| lg16 | 2D_56 | 36.796 | 2D | 47369409 |
| lg16 | 2D_83 | 37.039 | 2D | 103424559 |
| lg16 | 2D_81 | 37.18 | 2D | 100076103 |
| lg16 | 2D_82 | 37.47 | 2D | 100697411 |
| lg16 | 2D_76 | 37.972 | 2D | 92784031 |
| lg16 | 2D_127 | 38.107 | IWGSC_CSS_2DS_scaff_5364252 | 3980 |
| lg16 | 2D_65 | 38.278 | 2D | 67429960 |
| lg16 | 2D_88 | 38.322 | 2D | 114175563 |
| lg16 | 2D_78 | 38.331 | 2D | 94979628 |
| lg16 | 2D_124 | 38.503 | IWGSC_CSS_2DL_scaff_9852164 | 3411 |
| lg16 | 2D_87 | 38.521 | 2D | 114119074 |
| lg16 | 2D_85 | 38.634 | 2D | 107215786 |
| lg16 | 2D_90 | 38.717 | 2D | 117514514 |
| lg16 | 2D_66 | 38.792 | 2D | 70005762 |
| lg16 | 2D_64 | 38.926 | 2D | 67315195 |
| lg16 | 2D_79 | 39.286 | 2D | 96304625 |
| lg16 | 2D_63 | 39.579 | 2D | 64783460 |
| lg16 | 2D_52 | 45.659 | 2D | 32020637 |
| lg16 | 2D_71 | 46.273 | 2D | 78436544 |
| lg16 | 2D_50 | 47.491 | 2D | 24695996 |
| lg16 | 2D_53 | 47.999 | 2D | 37633012 |
| lg16 | 2D_51 | 48.619 | 2D | 26464412 |
| lg16 | 2D_89 | 49.751 | 2D | 115801312 |
| lg16 | 2D_93 | 52.18 | 2D | 132019236 |
| lg16 | 2D_92 | 53.662 | 2D | 131433891 |
| lg16 | 2D_91 | 57.111 | 2D | 130164574 |
| lg16 | 2D_94 | 59.382 | 2D | 133230793 |
| lg16 | 2D_97 | 60.848 | 2D | 139194814 |
| lg16 | 2D_118 | 69.257 | 2D | 144628470 |
| lg16 | 2D_121 | 69.308 | 2D | 144628600 |
| lg16 | 2D_111 | 69.67 | 2D | 142125105 |
| lg16 | 2D_109 | 69.683 | 2D | 142125075 |
| lg16 | 2D_107 | 69.683 | 2D | 142125001 |
| lg16 | 2D_106 | 69.686 | 2D | 142124926 |
| lg16 | 2D_110 | 69.981 | 2D | 142125083 |
| lg16 | 2D_108 | 69.984 | 2D | 142125036 |
| lg16 | 2D_99 | 69.996 | 2D | 140736014 |
| lg16 | 2D_104 | 70.026 | 2D | 141953300 |
| lg16 | 2D_98 | 70.039 | 2D | 140735816 |
| lg16 | 2D_105 | 70.062 | 2D | 141953373 |
| lg16 | 2D_102 | 70.071 | 2D | 141953177 |
| lg16 | 2D_101 | 70.071 | 2D | 141953176 |
| lg16 | 2D_100 | 70.071 | 2D | 141953168 |
| lg16 | 2D_103 | 70.076 | 2D | 141953212 |
| lg16 | 2D_113 | 70.143 | 2D | 143108887 |
| lg16 | 2D_117 | 70.15 | 2D | 143109021 |
| lg16 | 2D_116 | 70.15 | 2D | 143109019 |
| lg16 | 2D_115 | 70.4 | 2D | 143108954 |
| lg16 | 2D_114 | 70.452 | 2D | 143108902 |
| lg16 | 2D_120 | 70.707 | 2D | 144628526 |
| lg16 | 2D_119 | 70.741 | 2D | 144628483 |
| lg16 | 2D_112 | 71.083 | 2D | 142376838 |
| lg03 | 3A_12 | 0 | 3A | 15454756 |
| lg03 | 3A_13 | 0.14 | 3A | 15454793 |
| lg03 | 3A_11 | 0.152 | 3A | 15454660 |
| lg03 | 3A_10 | 0.254 | 3A | 14595668 |
| lg03 | 3A_14 | 2.655 | 3A | 16375289 |
| lg03 | 3A_47 | 3.909 | 3A | 102967413 |
| lg03 | 3A_18 | 4.281 | 3A | 26544574 |
| lg03 | 3A_21 | 4.489 | 3A | 42944895 |
| lg03 | 3A_39 | 4.725 | 3A | 75655074 |
| lg03 | 3A_40 | 4.725 | 3A | 75655088 |
| lg03 | 3A_38 | 4.771 | 3A | 75655065 |
| lg03 | 3A_20 | 4.941 | 3A | 42944872 |
| lg03 | 3A_22 | 5.085 | 3A | 42944963 |
| lg03 | 3A_35 | 5.102 | 3A | 65591840 |
| lg03 | 3A_48 | 6.084 | 3A | 103157887 |
| lg03 | 3A_31 | 6.1 | 3A | 63885486 |
| lg03 | 3A_45 | 6.653 | 3A | 102957138 |
| lg03 | 3A_46 | 6.742 | 3A | 102957159 |
| lg03 | 3A_28 | 7.153 | 3A | 59209323 |
| lg03 | 3A_34 | 7.423 | 3A | 65450431 |
| lg03 | 3A_26 | 7.46 | 3A | 52900025 |
| lg03 | 3A_25 | 7.481 | 3A | 52900021 |
| lg03 | 3A_5 | 7.529 | IWGSC_CSS_3AS_scaff_3441525 | 1002 |
| lg03 | 3A_33 | 7.55 | 3A | 64549407 |
| lg03 | 3A_49 | 7.674 | 3A | 107910739 |
| lg03 | 3A_50 | 7.68 | 3A | 107910862 |
| lg03 | 3A_41 | 7.698 | 3A | 76297005 |
| lg03 | 3A_29 | 7.801 | 3A | 59851997 |
| lg03 | 3A_37 | 7.962 | 3A | 73813594 |
| lg03 | 3A_36 | 8.043 | 3A | 73813577 |
| lg03 | 3A_30 | 8.185 | 3A | 62241340 |
| lg03 | 3A_32 | 8.239 | 3A | 63939427 |
| lg03 | 3A_44 | 8.639 | 3A | 99465601 |
| lg03 | 3A_54 | 8.83 | 3A | 125548321 |
| lg03 | 3A_52 | 8.934 | 3A | 110450167 |
| lg03 | 3A_27 | 9.097 | 3A | 59086390 |
| lg03 | 3A_19 | 9.149 | 3A | 37735686 |
| lg03 | 3A_53 | 9.281 | 3A | 118581132 |
| lg03 | 3A_42 | 9.605 | 3A | 78694380 |
| lg03 | 3A_51 | 9.802 | 3A | 108436242 |
| lg03 | 3A_6 | 9.97 | IWGSC_CSS_3AL_scaff_4397143 | 3832 |
| lg03 | 3A_43 | 10.137 | 3A | 91806553 |
| lg03 | 3A_23 | 10.652 | 3A | 44067845 |
| lg03 | 3A_24 | 12.272 | 3A | 51370988 |
| lg03 | 3A_59 | 23.51 | 3A | 142425030 |
| lg03 | 3A_60 | 30.197 | 3A | 150112221 |
| lg03 | 3A_56 | 35.267 | 3A | 140113494 |
| lg03 | 3A_58 | 35.288 | 3A | 140113605 |
| lg03 | 3A_55 | 35.288 | 3A | 140113429 |
| lg03 | 3A_57 | 35.299 | 3A | 140113535 |
| lg03 | 3A_62 | 43.906 | 3A | 160423843 |
| lg03 | 3A_61 | 43.955 | 3A | 160423633 |
| lg03 | 3A_63 | 55.849 | 3A | 164295855 |
| lg03 | 3A_64 | 56.702 | 3A | 164335006 |
| lg03 | 3A_65 | 59.148 | 3A | 164830884 |
| lg03 | 3A_66 | 73.497 | 3A | 171153437 |
| lg03 | 3A_67 | 73.888 | 3A | 172056429 |
| lg03 | 3A_78 | 86.933 | 3A | 177359710 |
| lg03 | 3A_74 | 87.919 | 3A | 176034854 |
| lg03 | 3A_75 | 87.943 | 3A | 176034860 |
| lg03 | 3A_76 | 88.052 | 3A | 176034982 |
| lg03 | 3A_77 | 88.052 | 3A | 176034984 |
| lg03 | 3A_71 | 88.061 | 3A | 176034832 |
| lg03 | 3A_69 | 88.061 | 3A | 176034770 |
| lg03 | 3A_72 | 88.065 | 3A | 176034834 |
| lg03 | 3A_70 | 88.092 | 3A | 176034783 |
| lg03 | 3A_73 | 88.178 | 3A | 176034840 |
| lg03 | 3A_68 | 88.6 | 3A | 175554454 |
| lg03 | 3A_79 | 89.776 | 3A | 177781289 |
| lg03 | 3A_80 | 92.324 | 3A | 178515097 |
| lg03 | 3A_82 | 99.939 | 3A | 180220827 |
| lg03 | 3A_81 | 99.95 | 3A | 180220826 |
| lg03 | 3A_83 | 99.994 | 3A | 180220828 |
| lg03 | 3A_100 | 100.473 | IWGSC_CSS_3AL_scaff_4369241 | 3036 |
| lg03 | 3A_101 | 100.499 | IWGSC_CSS_3AL_scaff_4369241 | 3060 |
| lg03 | 3A_87 | 106.318 | 3A | 181657207 |
| lg03 | 3A_86 | 106.371 | 3A | 181657200 |
| lg03 | 3A_85 | 106.428 | 3A | 181622403 |
| lg03 | 3A_84 | 106.488 | 3A | 181622402 |
| lg03 | 3A_2 | 118.908 | IWGSC_CSS_3AL_scaff_4297021 | 2465 |
| lg03 | 3A_1 | 119.402 | IWGSC_CSS_3AL_scaff_4297021 | 1105 |
| lg03 | 3A_88 | 120.907 | 3A | 182920486 |
| lg03 | 3A_89 | 122.066 | 3A | 183522719 |
| lg03 | 3A_3 | 122.114 | IWGSC_CSS_3AL_scaff_4424987 | 3781 |
| lg03 | 3A_94 | 122.567 | 3A | 183523434 |
| lg03 | 3A_93 | 122.614 | 3A | 183523419 |
| lg03 | 3A_92 | 122.647 | 3A | 183523402 |
| lg03 | 3A_95 | 122.657 | 3A | 183523441 |
| lg03 | 3A_90 | 122.706 | 3A | 183523322 |
| lg03 | 3A_91 | 122.712 | 3A | 183523400 |
| lg03 | 3A_96 | 123.086 | 3A | 183921280 |
| lg03 | 3A_97 | 124.036 | 3A | 184609182 |
| lg03 | 3A_98 | 124.178 | 3A | 184609275 |
| lg03 | 3A_99 | 124.196 | 3A | 184609376 |
| lg10 | 3B_40 | 0 | 3B | 10899443 |
| lg10 | 3B_39 | 2.012 | 3B | 10836408 |
| lg10 | 3B_44 | 3.325 | 3B | 13075722 |
| lg10 | 3B_43 | 3.341 | 3B | 13075720 |
| lg10 | 3B_17 | 4.122 | 3B | 7596034 |
| lg10 | 3B_16 | 4.128 | 3B | 7596009 |
| lg10 | 3B_14 | 4.131 | 3B | 7595960 |
| lg10 | 3B_19 | 4.382 | 3B | 10475658 |
| lg10 | 3B_18 | 4.382 | 3B | 9619316 |
| lg10 | 3B_10 | 5.808 | IWGSC_CSS_3B_scaff_10475865 | 1404 |
| lg10 | 3B_38 | 7.062 | 3B | 10724473 |
| lg10 | 3B_37 | 7.479 | 3B | 10724433 |
| lg10 | 3B_48 | 7.934 | 3B | 13342001 |
| lg10 | 3B_41 | 8.844 | 3B | 10907624 |
| lg10 | 3B_35 | 9.567 | 3B | 10717590 |
| lg10 | 3B_51 | 10.466 | 3B | 13393288 |
| lg10 | 3B_49 | 10.468 | 3B | 13393170 |
| lg10 | 3B_50 | 10.469 | 3B | 13393255 |
| lg10 | 3B_33 | 11.594 | 3B | 10717553 |
| lg10 | 3B_34 | 11.66 | 3B | 10717570 |
| lg10 | 3B_36 | 11.684 | 3B | 10717595 |
| lg10 | 3B_20 | 12.347 | 3B | 10675035 |
| lg10 | 3B_45 | 12.989 | 3B | 13341838 |
| lg10 | 3B_47 | 12.989 | 3B | 13341983 |
| lg10 | 3B_46 | 12.989 | 3B | 13341895 |
| lg10 | 3B_52 | 13.466 | 3B | 14282377 |
| lg10 | 3B_29 | 14.463 | 3B | 10711502 |
| lg10 | 3B_30 | 14.878 | 3B | 10711541 |
| lg10 | 3B_32 | 14.926 | 3B | 10711583 |
| lg10 | 3B_42 | 15.511 | 3B | 12823425 |
| lg10 | 3B_25 | 16.155 | 3B | 10711457 |
| lg10 | 3B_21 | 17.283 | 3B | 10675740 |
| lg10 | 3B_23 | 18.026 | 3B | 10711428 |
| lg10 | 3B_31 | 18.028 | 3B | 10711546 |
| lg10 | 3B_26 | 18.028 | 3B | 10711479 |
| lg10 | 3B_22 | 18.028 | 3B | 10711413 |
| lg10 | 3B_28 | 18.033 | 3B | 10711487 |
| lg10 | 3B_27 | 18.035 | 3B | 10711480 |
| lg10 | 3B_24 | 18.038 | 3B | 10711432 |
| lg10 | 3B_53 | 22.11 | 3B | 19853349 |
| lg10 | 3B_54 | 23.019 | 3B | 21581584 |
| lg10 | 3B_55 | 27.1 | 3B | 23697719 |
| lg10 | 3B_56 | 28.074 | 3B | 25400353 |
| lg10 | 3B_58 | 28.091 | 3B | 25400520 |
| lg10 | 3B_57 | 28.091 | 3B | 25400519 |
| lg10 | 3B_60 | 29.815 | 3B | 26478499 |
| lg10 | 3B_61 | 29.819 | 3B | 26478536 |
| lg10 | 3B_66 | 30.203 | 3B | 26507050 |
| lg10 | 3B_64 | 30.206 | 3B | 26506922 |
| lg10 | 3B_67 | 30.206 | 3B | 26507147 |
| lg10 | 3B_73 | 31.456 | 3B | 28589940 |
| lg10 | 3B_72 | 31.468 | 3B | 28589930 |
| lg10 | 3B_69 | 31.471 | 3B | 28396753 |
| lg10 | 3B_68 | 31.474 | 3B | 28396686 |
| lg10 | 3B_70 | 31.476 | 3B | 28396803 |
| lg10 | 3B_74 | 39.873 | 3B | 38301797 |
| lg10 | 3B_75 | 42.048 | 3B | 39154422 |
| lg10 | 3B_79 | 47.462 | 3B | 43701816 |
| lg10 | 3B_78 | 47.465 | 3B | 43701718 |
| lg10 | 3B_96 | 48.382 | 3B | 47243422 |
| lg10 | 3B_77 | 50.322 | 3B | 42777581 |
| lg10 | 3B_82 | 52.268 | 3B | 44331323 |
| lg10 | 3B_84 | 52.268 | 3B | 44331347 |
| lg10 | 3B_83 | 52.268 | 3B | 44331338 |
| lg10 | 3B_90 | 52.947 | 3B | 44425993 |
| lg10 | 3B_94 | 53.231 | 3B | 45197189 |
| lg10 | 3B_93 | 53.273 | 3B | 44554277 |
| lg10 | 3B_92 | 53.273 | 3B | 44554204 |
| lg10 | 3B_86 | 53.388 | 3B | 44425914 |
| lg10 | 3B_87 | 53.527 | 3B | 44425932 |
| lg10 | 3B_88 | 53.559 | 3B | 44425977 |
| lg10 | 3B_95 | 53.886 | 3B | 45333643 |
| lg10 | 3B_80 | 55.047 | 3B | 43764018 |
| lg10 | 3B_91 | 56.029 | 3B | 44426121 |
| lg10 | 3B_85 | 56.055 | 3B | 44425883 |
| lg10 | 3B_89 | 56.058 | 3B | 44425989 |
| lg10 | 3B_97 | 56.365 | 3B | 47272919 |
| lg10 | 3B_98 | 60.512 | 3B | 58387608 |
| lg10 | 3B_102 | 66.982 | 3B | 62941422 |
| lg10 | 3B_101 | 67.705 | 3B | 62452815 |
| lg10 | 3B_99 | 69.874 | 3B | 59672555 |
| lg10 | 3B_100 | 69.874 | 3B | 59672560 |
| lg10 | 3B_106 | 76.089 | 3B | 75480944 |
| lg10 | 3B_107 | 76.094 | 3B | 75480993 |
| lg10 | 3B_104 | 78.237 | 3B | 71805922 |
| lg10 | 3B_105 | 78.717 | 3B | 73778509 |
| lg10 | 3B_111 | 80.436 | 3B | 81592100 |
| lg10 | 3B_109 | 81.106 | 3B | 81291841 |
| lg10 | 3B_108 | 82.002 | 3B | 79964946 |
| lg10 | 3B_112 | 83.583 | 3B | 92907911 |
| lg10 | 3B_110 | 85.398 | 3B | 81433362 |
| lg10 | 3B_120 | 86.897 | 3B | 118141330 |
| lg10 | 3B_133 | 88.536 | 3B | 130223933 |
| lg10 | 3B_138 | 89.321 | 3B | 131418075 |
| lg10 | 3B_136 | 89.321 | 3B | 131418049 |
| lg10 | 3B_137 | 89.321 | 3B | 131418064 |
| lg10 | 3B_130 | 90.561 | 3B | 124316617 |
| lg10 | 3B_129 | 90.861 | 3B | 124316516 |
| lg10 | 3B_140 | 91.845 | 3B | 132085791 |
| lg10 | 3B_128 | 92.979 | 3B | 121956803 |
| lg10 | 3B_121 | 93.087 | 3B | 120565483 |
| lg10 | 3B_134 | 94.012 | 3B | 130691760 |
| lg10 | 3B_135 | 94.042 | 3B | 130691767 |
| lg10 | 3B_118 | 97.536 | 3B | 117265889 |
| lg10 | 3B_117 | 100.455 | 3B | 105204139 |
| lg10 | 3B_119 | 101.398 | 3B | 117780502 |
| lg10 | 3B_114 | 102.702 | 3B | 97200911 |
| lg10 | 3B_115 | 103.614 | 3B | 97534734 |
| lg10 | 3B_113 | 105.139 | 3B | 96123350 |
| lg10 | 3B_148 | 106.814 | 3B | 150673317 |
| lg10 | 3B_141 | 106.876 | 3B | 134956474 |
| lg10 | 3B_127 | 106.919 | 3B | 121220211 |
| lg10 | 3B_131 | 107.701 | 3B | 124348104 |
| lg10 | 3B_123 | 108.293 | 3B | 120999273 |
| lg10 | 3B_125 | 108.461 | 3B | 120999315 |
| lg10 | 3B_146 | 108.602 | 3B | 139316759 |
| lg10 | 3B_124 | 108.606 | 3B | 120999300 |
| lg10 | 3B_122 | 108.617 | 3B | 120999227 |
| lg10 | 3B_126 | 108.632 | 3B | 120999370 |
| lg10 | 3B_132 | 110.716 | 3B | 125779980 |
| lg10 | 3B_162 | 115.504 | 3B | 162213142 |
| lg10 | 3B_159 | 115.524 | 3B | 162213089 |
| lg10 | 3B_160 | 115.526 | 3B | 162213105 |
| lg10 | 3B_161 | 115.627 | 3B | 162213137 |
| lg10 | 3B_163 | 115.711 | 3B | 162213220 |
| lg10 | 3B_154 | 117.086 | 3B | 161247430 |
| lg10 | 3B_157 | 117.508 | 3B | 162106097 |
| lg10 | 3B_153 | 118.806 | 3B | 159596314 |
| lg10 | 3B_150 | 119.982 | 3B | 152280966 |
| lg10 | 3B_152 | 120.199 | 3B | 159269186 |
| lg10 | 3B_164 | 120.617 | 3B | 162553457 |
| lg10 | 3B_165 | 121.072 | 3B | 167484015 |
| lg10 | 3B_155 | 122.4 | 3B | 161684570 |
| lg10 | 3B_156 | 123.763 | 3B | 161684585 |
| lg10 | 3B_151 | 124.725 | 3B | 155136166 |
| lg10 | 3B_149 | 126.11 | 3B | 152052087 |
| lg10 | 3B_142 | 127.797 | 3B | 136058989 |
| lg10 | 3B_143 | 127.798 | 3B | 136059022 |
| lg10 | 3B_171 | 130.553 | 3B | 181827608 |
| lg10 | 3B_184 | 131.573 | 3B | 188444096 |
| lg10 | 3B_226 | 132.207 | 3B | 245464816 |
| lg10 | 3B_205 | 132.372 | 3B | 227258882 |
| lg10 | 3B_174 | 133.49 | 3B | 185478413 |
| lg10 | 3B_183 | 134.259 | 3B | 187252653 |
| lg10 | 3B_179 | 134.287 | 3B | 187252479 |
| lg10 | 3B_182 | 134.312 | 3B | 187252642 |
| lg10 | 3B_181 | 134.335 | 3B | 187252505 |
| lg10 | 3B_180 | 134.367 | 3B | 187252498 |
| lg10 | 3B_185 | 135.242 | 3B | 188839797 |
| lg10 | 3B_169 | 135.953 | 3B | 181172789 |
| lg10 | 3B_203 | 138.218 | 3B | 214637301 |
| lg10 | 3B_208 | 140.357 | 3B | 228562880 |
| lg10 | 3B_223 | 141.237 | 3B | 241564702 |
| lg10 | 3B_219 | 142.097 | 3B | 236141408 |
| lg10 | 3B_220 | 142.158 | 3B | 236141570 |
| lg10 | 3B_201 | 143.088 | 3B | 209713915 |
| lg10 | 3B_214 | 143.46 | 3B | 234027733 |
| lg10 | 3B_215 | 143.462 | 3B | 234027748 |
| lg10 | 3B_176 | 144.722 | 3B | 186741811 |
| lg10 | 3B_178 | 144.724 | 3B | 186741934 |
| lg10 | 3B_175 | 144.726 | 3B | 186741725 |
| lg10 | 3B_199 | 146.095 | 3B | 204906322 |
| lg10 | 3B_198 | 146.095 | 3B | 204906277 |
| lg10 | 3B_172 | 146.828 | 3B | 184082342 |
| lg10 | 3B_218 | 147.884 | 3B | 234660743 |
| lg10 | 3B_222 | 148.109 | 3B | 241276718 |
| lg10 | 3B_202 | 150.65 | 3B | 214637241 |
| lg10 | 3B_212 | 153.094 | 3B | 230056184 |
| lg10 | 3B_204 | 154.529 | 3B | 216787604 |
| lg10 | 3B_189 | 154.579 | 3B | 196720028 |
| lg10 | 3B_228 | 156.123 | 3B | 249232129 |
| lg10 | 3B_196 | 157.621 | 3B | 199969983 |
| lg10 | 3B_195 | 157.745 | 3B | 199942527 |
| lg10 | 3B_200 | 158.878 | 3B | 208916042 |
| lg10 | 3B_213 | 159.935 | 3B | 230394223 |
| lg10 | 3B_188 | 160.047 | 3B | 192485326 |
| lg10 | 3B_210 | 160.377 | 3B | 228563004 |
| lg10 | 3B_209 | 160.382 | 3B | 228562980 |
| lg10 | 3B_206 | 160.383 | 3B | 228562838 |
| lg10 | 3B_207 | 160.383 | 3B | 228562861 |
| lg10 | 3B_103 | 160.76 | 3B | 70078849 |
| lg10 | 3B_2 | 161.036 | IWGSC_CSS_3B_scaff_10608564 | 7939 |
| lg10 | 3B_1 | 161.04 | IWGSC_CSS_3B_scaff_10608564 | 7900 |
| lg10 | 3B_3 | 161.041 | IWGSC_CSS_3B_scaff_10608564 | 8075 |
| lg10 | 3B_194 | 162.408 | 3B | 198247055 |
| lg10 | 3B_193 | 162.451 | 3B | 198247053 |
| lg10 | 3B_186 | 163.105 | 3B | 189598405 |
| lg10 | 3B_224 | 163.751 | 3B | 244110364 |
| lg10 | 3B_191 | 164.506 | 3B | 198031509 |
| lg10 | 3B_190 | 165.099 | 3B | 197075842 |
| lg10 | 3B_192 | 165.747 | 3B | 198246992 |
| lg10 | 3B_170 | 165.889 | 3B | 181825390 |
| lg10 | 3B_216 | 167.055 | 3B | 234027794 |
| lg10 | 3B_217 | 167.079 | 3B | 234027823 |
| lg10 | 3B_225 | 168.332 | 3B | 245082850 |
| lg10 | 3B_187 | 169.384 | 3B | 189963797 |
| lg10 | 3B_168 | 170.575 | 3B | 172721029 |
| lg10 | 3B_243 | 172.678 | IWGSC_CSS_3B_scaff_10685350 | 2772 |
| lg10 | 3B_145 | 173.925 | 3B | 139013850 |
| lg10 | 3B_144 | 173.926 | 3B | 139013833 |
| lg10 | 3B_158 | 175.049 | 3B | 162106101 |
| lg10 | 3B_147 | 176.037 | 3B | 150005286 |
| lg10 | 3B_167 | 177.487 | 3B | 170079120 |
| lg10 | 3B_166 | 180.498 | 3B | 168287099 |
| lg10 | 3B_12 | 193.249 | 3B | 6791668 |
| lg10 | 3B_235 | 193.257 | 3B | 362364163 |
| lg10 | 3B_234 | 193.51 | 3B | 362364160 |
| lg10 | 3B_230 | 193.882 | 3B | 362323835 |
| lg10 | 3B_233 | 194.409 | 3B | 362364143 |
| lg10 | 3B_13 | 194.919 | 3B | 7105264 |
| lg10 | 3B_238 | 198.747 | 3B | 394158203 |
| lg10 | 3B_239 | 198.766 | 3B | 394158319 |
| lg10 | 3B_240 | 200.137 | 3B | 394184034 |
| lg10 | 3B_8 | 204.608 | IWGSC_CSS_3B_scaff_10422433 | 2930 |
| lg10 | 3B_7 | 204.608 | IWGSC_CSS_3B_scaff_10422433 | 2879 |
| lg17 | 3D_28 | 0 | 3D | 93432297 |
| lg17 | 3D_29 | 5.336 | 3D | 94118128 |
| lg17 | 3D_27 | 10.072 | 3D | 91056230 |
| lg17 | 3D_19 | 23.078 | 3D | 55322293 |
| lg17 | 3D_14 | 25.077 | 3D | 34944729 |
| lg17 | 3D_15 | 27.388 | 3D | 41539475 |
| lg17 | 3D_24 | 28.216 | 3D | 72298153 |
| lg17 | 3D_17 | 29.29 | 3D | 45857886 |
| lg17 | 3D_44 | 30.403 | IWGSC_CSS_3DL_scaff_6907474 | 2197 |
| lg17 | 3D_4 | 31.53 | 3D | 13954981 |
| lg17 | 3D_11 | 32.351 | 3D | 27483284 |
| lg17 | 3D_10 | 34.646 | 3D | 23250352 |
| lg17 | 3D_20 | 34.928 | 3D | 58143393 |
| lg17 | 3D_13 | 35.126 | 3D | 34386504 |
| lg17 | 3D_26 | 35.144 | 3D | 83328228 |
| lg17 | 3D_12 | 35.209 | 3D | 34386403 |
| lg17 | 3D_18 | 35.469 | 3D | 53330238 |
| lg17 | 3D_16 | 35.696 | 3D | 43875120 |
| lg17 | 3D_7 | 35.917 | 3D | 19761010 |
| lg17 | 3D_9 | 35.922 | 3D | 19761012 |
| lg17 | 3D_8 | 35.927 | 3D | 19761011 |
| lg17 | 3D_22 | 35.931 | 3D | 66469099 |
| lg17 | 3D_5 | 36.41 | 3D | 16071149 |
| lg17 | 3D_21 | 36.603 | 3D | 60948000 |
| lg17 | 3D_45 | 37.674 | IWGSC_CSS_3DS_scaff_2603003 | 5168 |
| lg17 | 3D_6 | 38.698 | 3D | 18466337 |
| lg17 | 3D_23 | 40.78 | 3D | 66674374 |
| lg04 | 4A_7 | 0 | IWGSC_CSS_4AS_scaff_5970687 | 8264 |
| lg04 | 4A_12 | 3.30 | 4A | 32510412 |
| lg04 | 4A_16 | 7.954 | 4A | 58854127 |
| lg04 | 4A_28 | 9.968 | 4A | 119665730 |
| lg04 | 4A_36 | 10.11 | 4A | 157351316 |
| lg04 | 4A_34 | 10.30 | 4A | 149816395 |
| lg04 | 4A_10 | 10.316 | 4A | 8129032 |
| lg04 | 4A_31 | 10.712 | 4A | 140435022 |
| lg04 | 4A_29 | 10.715 | 4A | 140434779 |
| lg04 | 4A_30 | 10.717 | 4A | 140434800 |
| lg04 | 4A_39 | 10.944 | 4A | 162571642 |
| lg04 | 4A_38 | 11.1 | 4A | 158812250 |
| lg04 | 4A_33 | 11.225 | 4A | 149357651 |
| lg04 | 4A_22 | 11.581 | 4A | 94420329 |
| lg04 | 4A_32 | 11.584 | 4A | 143359731 |
| lg04 | 4A_18 | 11.811 | 4A | 76299872 |
| lg04 | 4A_35 | 11.827 | 4A | 155589459 |
| lg04 | 4A_2 | 11.836 | IWGSC_CSS_4AL_scaff_7173869 | 3770 |
| lg04 | 4A_23 | 12.11 | 4A | 104727516 |
| lg04 | 4A_19 | 12.826 | 4A | 78725545 |
| lg04 | 4A_9 | 13.012 | IWGSC_CSS_4AL_scaff_7077010 | 7130 |
| lg04 | 4A_6 | 13.109 | IWGSC_CSS_4AL_scaff_7106622 | 4014 |
| lg04 | 4A_80 | 13.211 | IWGSC_CSS_4AL_scaff_7158625 | 1604 |
| lg04 | 4A_25 | 13.319 | 4A | 112524215 |
| lg04 | 4A_79 | 13.321 | IWGSC_CSS_4AS_scaff_5983419 | 3291 |
| lg04 | 4A_27 | 13.33 | 4A | 118636683 |
| lg04 | 4A_14 | 13.401 | 4A | 37443057 |
| lg04 | 4A_20 | 13.534 | 4A | 81035072 |
| lg04 | 4A_82 | 13.541 | IWGSC_CSS_4AS_scaff_5939355 | 9864 |
| lg04 | 4A_26 | 13.627 | 4A | 118624684 |
| lg04 | 4A_1 | 13.701 | IWGSC_CSS_4AS_scaff_6015240 | 627 |
| lg04 | 4A_21 | 13.82 | 4A | 88123539 |
| lg04 | 4A_13 | 13.912 | 4A | 36922236 |
| lg04 | 4A_11 | 14.125 | 4A | 15211687 |
| lg04 | 4A_83 | 14.215 | IWGSC_CSS_4AS_scaff_5964437 | 2621 |
| lg04 | 4A_17 | 14.813 | 4A | 60182387 |
| lg04 | 4A_40 | 18.171 | 4A | 168037510 |
| lg04 | 4A_42 | 18.298 | 4A | 168037558 |
| lg04 | 4A_41 | 18.321 | 4A | 168037551 |
| lg04 | 4A_109 | 19.212 | 4A | 162840757 |
| lg04 | 4A_119 | 21.2 | 4A | 168037558 |
| lg04 | 4A_37 | 27.122 | 4A | 157657246 |
| lg04 | 4A_75 | 30.612 | IWGSC_CSS_4AL_scaff_7124932 | 411 |
| lg04 | 4A_74 | 30.619 | IWGSC_CSS_4AL_scaff_7124932 | 399 |
| lg04 | 4A_76 | 30.823 | IWGSC_CSS_4AL_scaff_7124932 | 523 |
| lg04 | 4A_43 | 42.516 | 4A | 171262760 |
| lg04 | 4A_44 | 43.233 | 4A | 172040270 |
| lg04 | 4A_81 | 46.524 | IWGSC_CSS_4AL_scaff_7131732 | 160 |
| lg04 | 4A_3 | 56.229 | IWGSC_CSS_4AL_scaff_7177306 | 15952 |
| lg04 | 4A_5 | 63.735 | IWGSC_CSS_4AL_scaff_7165527 | 6703 |
| lg04 | 4A_4 | 63.819 | IWGSC_CSS_4AL_scaff_7165527 | 6628 |
| lg04 | 4A_8 | 67.816 | IWGSC_CSS_4AL_scaff_7171359 | 1110 |
| lg04 | 4A_50 | 80.613 | 4A | 196033012 |
| lg04 | 4A_51 | 87.035 | 4A | 199133362 |
| lg04 | 4A_45 | 87.221 | 4A | 180288435 |
| lg04 | 4A_48 | 87.82 | 4A | 186619062 |
| lg04 | 4A_78 | 88.092 | IWGSC_CSS_4AL_scaff_7148407 | 486 |
| lg04 | 4A_46 | 88.129 | 4A | 182706322 |
| lg04 | 4A_47 | 88.220 | 4A | 182706380 |
| lg04 | 4A_49 | 92.81 | 4A | 188270762 |
| lg04 | 4A_52 | 101.513 | 4A | 201290766 |
| lg04 | 4A_54 | 102.549 | 4A | 201841159 |
| lg04 | 4A_62 | 102.916 | 4A | 202071628 |
| lg04 | 4A_61 | 103.224 | 4A | 202071517 |
| lg04 | 4A_67 | 103.428 | 4A | 205912744 |
| lg04 | 4A_69 | 103.61 | 4A | 206789823 |
| lg04 | 4A_64 | 103.721 | 4A | 202637177 |
| lg04 | 4A_63 | 103.745 | 4A | 202637174 |
| lg04 | 4A_66 | 103.81 | 4A | 202809663 |
| lg04 | 4A_65 | 103.84 | 4A | 202637190 |
| lg04 | 4A_56 | 104.017 | 4A | 201924569 |
| lg04 | 4A_57 | 104.018 | 4A | 201924629 |
| lg04 | 4A_59 | 104.101 | 4A | 201924729 |
| lg04 | 4A_58 | 104.103 | 4A | 201924690 |
| lg04 | 4A_60 | 104.104 | 4A | 201924768 |
| lg04 | 4A_68 | 104.314 | 4A | 205912750 |
| lg04 | 4A_55 | 104.506 | 4A | 201841221 |
| lg04 | 4A_72 | 105.615 | 4A | 207064606 |
| lg04 | 4A_71 | 105.626 | 4A | 207064591 |
| lg04 | 4A_73 | 105.649 | 4A | 207064608 |
| lg04 | 4A_70 | 105.71 | 4A | 207064580 |
| lg11 | 4B_106 | 0 | 4B | 314762154 |
| lg11 | 4B_109 | 2.62 | 4B | 316138447 |
| lg11 | 4B_108 | 2.967 | 4B | 316136844 |
| lg11 | 4B_1 | 4.008 | IWGSC_CSS_4BL_scaff_7007648 | 4251 |
| lg11 | 4B_110 | 6.846 | 4B | 317093189 |
| lg11 | 4B_105 | 19.429 | 4B | 314642844 |
| lg11 | 4B_2 | 20.459 | IWGSC_CSS_4BL_scaff_6979541 | 1338 |
| lg11 | 4B_104 | 24.379 | 4B | 311638680 |
| lg11 | 4B_103 | 39.576 | 4B | 304260082 |
| lg11 | 4B_96 | 42.004 | 4B | 300537492 |
| lg11 | 4B_97 | 42.572 | 4B | 300978423 |
| lg11 | 4B_99 | 42.615 | 4B | 300978590 |
| lg11 | 4B_100 | 42.615 | 4B | 300978594 |
| lg11 | 4B_101 | 42.615 | 4B | 300978645 |
| lg11 | 4B_98 | 42.615 | 4B | 300978572 |
| lg11 | 4B_66 | 43.712 | 4B | 272384561 |
| lg11 | 4B_65 | 43.772 | 4B | 272384470 |
| lg11 | 4B_48 | 43.978 | 4B | 125735596 |
| lg11 | 4B_69 | 44.534 | 4B | 275989758 |
| lg11 | 4B_71 | 44.565 | 4B | 275989915 |
| lg11 | 4B_72 | 44.576 | 4B | 275989969 |
| lg11 | 4B_70 | 44.592 | 4B | 275989842 |
| lg11 | 4B_77 | 44.971 | 4B | 277312390 |
| lg11 | 4B_87 | 45.133 | 4B | 286640607 |
| lg11 | 4B_81 | 45.242 | 4B | 284665480 |
| lg11 | 4B_60 | 45.392 | 4B | 271167748 |
| lg11 | 4B_93 | 45.456 | 4B | 290922311 |
| lg11 | 4B_95 | 45.476 | 4B | 295274561 |
| lg11 | 4B_94 | 45.582 | 4B | 295130281 |
| lg11 | 4B_76 | 45.636 | 4B | 276718466 |
| lg11 | 4B_75 | 45.636 | 4B | 276718444 |
| lg11 | 4B_73 | 45.75 | 4B | 276718265 |
| lg11 | 4B_102 | 45.937 | 4B | 301837956 |
| lg11 | 4B_64 | 46.096 | 4B | 271992976 |
| lg11 | 4B_74 | 46.239 | 4B | 276718360 |
| lg11 | 4B_111 | 47.609 | IWGSC_CSS_4BL_scaff_7017238 | 1960 |
| lg11 | 4B_78 | 47.96 | 4B | 278366738 |
| lg11 | 4B_88 | 48.185 | 4B | 286737596 |
| lg11 | 4B_59 | 48.356 | 4B | 266917961 |
| lg11 | 4B_61 | 48.466 | 4B | 271189754 |
| lg11 | 4B_80 | 48.555 | 4B | 279258846 |
| lg11 | 4B_79 | 48.613 | 4B | 279258786 |
| lg11 | 4B_86 | 48.71 | 4B | 285833629 |
| lg11 | 4B_82 | 48.732 | 4B | 285833472 |
| lg11 | 4B_85 | 48.751 | 4B | 285833595 |
| lg11 | 4B_83 | 48.763 | 4B | 285833547 |
| lg11 | 4B_84 | 48.772 | 4B | 285833580 |
| lg11 | 4B_68 | 49.138 | 4B | 275042003 |
| lg11 | 4B_62 | 49.369 | 4B | 271435803 |
| lg11 | 4B_92 | 49.564 | 4B | 289514425 |
| lg11 | 4B_91 | 49.564 | 4B | 289514410 |
| lg11 | 4B_89 | 49.871 | 4B | 288047435 |
| lg11 | 4B_90 | 49.895 | 4B | 288047460 |
| lg11 | 4B_57 | 53.566 | 4B | 249154954 |
| lg11 | 4B_113 | 57.489 | IWGSC_CSS_4BL_scaff_6972931 | 3169 |
| lg11 | 4B_58 | 58.366 | 4B | 262477265 |
| lg11 | 4B_67 | 58.609 | 4B | 274927521 |
| lg11 | 4B_42 | 60.766 | 4B | 91258070 |
| lg11 | 4B_36 | 61.186 | 4B | 34629279 |
| lg11 | 4B_49 | 61.329 | 4B | 132092481 |
| lg11 | 4B_41 | 61.492 | 4B | 85932995 |
| lg11 | 4B_37 | 61.642 | 4B | 37556865 |
| lg11 | 4B_56 | 61.705 | 4B | 204184887 |
| lg11 | 4B_54 | 61.856 | 4B | 194121191 |
| lg11 | 4B_55 | 61.993 | 4B | 195158635 |
| lg11 | 4B_46 | 62.247 | 4B | 118720645 |
| lg11 | 4B_43 | 62.564 | 4B | 94968061 |
| lg11 | 4B_40 | 62.962 | 4B | 79297622 |
| lg11 | 4B_51 | 64.072 | 4B | 170140567 |
| lg11 | 4B_52 | 64.308 | 4B | 176157318 |
| lg11 | 4B_47 | 65.003 | 4B | 120176012 |
| lg11 | 4B_112 | 65.757 | IWGSC_CSS_4BS_scaff_4954395 | 740 |
| lg11 | 4B_53 | 66.519 | 4B | 182028383 |
| lg11 | 4B_39 | 67.444 | 4B | 57253925 |
| lg11 | 4B_26 | 79.932 | 4B | 9898554 |
| lg11 | 4B_25 | 80.475 | 4B | 9898516 |
| lg11 | 4B_24 | 80.563 | 4B | 9898416 |
| lg11 | 4B_23 | 80.589 | 4B | 9898409 |
| lg11 | 4B_29 | 80.607 | 4B | 9898575 |
| lg11 | 4B_31 | 80.635 | 4B | 9898601 |
| lg11 | 4B_30 | 80.672 | 4B | 9898597 |
| lg11 | 4B_27 | 80.719 | 4B | 9898557 |
| lg11 | 4B_28 | 81.396 | 4B | 9898566 |
| lg11 | 4B_18 | 88.744 | 4B | 6244193 |
| lg11 | 4B_19 | 89.038 | 4B | 6244229 |
| lg11 | 4B_21 | 89.136 | 4B | 6244271 |
| lg11 | 4B_11 | 89.637 | 4B | 4760858 |
| lg11 | 4B_10 | 89.686 | 4B | 4760815 |
| lg11 | 4B_8 | 89.686 | 4B | 4760754 |
| lg11 | 4B_14 | 89.698 | 4B | 4760945 |
| lg11 | 4B_13 | 89.746 | 4B | 4760892 |
| lg11 | 4B_7 | 89.757 | 4B | 4760752 |
| lg11 | 4B_9 | 89.761 | 4B | 4760780 |
| lg11 | 4B_12 | 89.793 | 4B | 4760868 |
| lg11 | 4B_15 | 89.826 | 4B | 4760955 |
| lg11 | 4B_16 | 89.886 | 4B | 6064426 |
| lg11 | 4B_22 | 90.714 | 4B | 6244288 |
| lg11 | 4B_20 | 90.754 | 4B | 6244243 |
| lg11 | 4B_17 | 90.914 | 4B | 6244180 |
| lg11 | 4B_5 | 101.017 | 4B | 2589026 |
| lg11 | 4B_45 | 110.444 | 4B | 115545635 |
| lg18 | 4D_3 | 0 | 4D | 4685104 |
| lg18 | 4D_2 | 3.045 | 4D | 597353 |
| lg18 | 4D_1 | 8.947 | 4D | 422338 |
| lg18 | 4D_4 | 9.894 | 4D | 8179897 |
| lg18 | 4D_5 | 16.061 | 4D | 16867828 |
| lg18 | 4D_17 | 26.836 | IWGSC_CSS_4DL_scaff_14301861 | 497 |
| lg18 | 4D_6 | 32.882 | 4D | 19002053 |
| lg18 | 4D_9 | 38.57 | 4D | 113713671 |
| lg18 | 4D_8 | 46.644 | 4D | 113313253 |
| lg18 | 4D_10 | 63.165 | IWGSC_CSS_4DL_scaff_14371651 | 3875 |
| lg18 | 4D_18 | 66.186 | IWGSC_CSS_4DL_scaff_14457020 | 7613 |
| lg05 | 5A_181 | 0 | IWGSC_CSS_5AS_scaff_1504222 | 288 |
| lg05 | 5A_180 | 0.005 | IWGSC_CSS_5AS_scaff_1504222 | 277 |
| lg05 | 5A_182 | 1.407 | IWGSC_CSS_5AS_scaff_1504222 | 1759 |
| lg05 | 5A_49 | 2.313 | 5A | 37499301 |
| lg05 | 5A_199 | 13.739 | IWGSC_CSS_5AS_scaff_1509222 | 5116 |
| lg05 | 5A_16 | 14.214 | IWGSC_CSS_5AS_scaff_1553294 | 1945 |
| lg05 | 5A_64 | 14.646 | 5A | 61225721 |
| lg05 | 5A_65 | 14.932 | 5A | 61225744 |
| lg05 | 5A_66 | 15.23 | 5A | 61225790 |
| lg05 | 5A_48 | 16.614 | 5A | 33815506 |
| lg05 | 5A_201 | 18.096 | IWGSC_CSS_5AS_scaff_1515704 | 3786 |
| lg05 | 5A_56 | 24.533 | 5A | 49229965 |
| lg05 | 5A_215 | 24.879 | IWGSC_CSS_5AS_scaff_1516477 | 3033 |
| lg05 | 5A_15 | 24.897 | IWGSC_CSS_5AS_scaff_1506407 | 414 |
| lg05 | 5A_13 | 24.897 | IWGSC_CSS_5AS_scaff_1506407 | 240 |
| lg05 | 5A_161 | 25.658 | IWGSC_CSS_5AS_scaff_1503189 | 4699 |
| lg05 | 5A_14 | 26.635 | IWGSC_CSS_5AS_scaff_1506407 | 362 |
| lg05 | 5A_5 | 26.805 | IWGSC_CSS_5AS_scaff_1510502 | 2134 |
| lg05 | 5A_2 | 26.852 | IWGSC_CSS_5AS_scaff_1510502 | 2095 |
| lg05 | 5A_7 | 26.875 | IWGSC_CSS_5AS_scaff_1510502 | 2282 |
| lg05 | 5A_3 | 26.905 | IWGSC_CSS_5AS_scaff_1510502 | 2113 |
| lg05 | 5A_4 | 26.925 | IWGSC_CSS_5AS_scaff_1510502 | 2114 |
| lg05 | 5A_54 | 27.09 | 5A | 44895680 |
| lg05 | 5A_170 | 27.691 | IWGSC_CSS_5AS_scaff_1536214 | 2526 |
| lg05 | 5A_216 | 28.259 | IWGSC_CSS_5AS_scaff_1514363 | 6189 |
| lg05 | 5A_35 | 28.719 | 5A | 1976087 |
| lg05 | 5A_70 | 29.401 | 5A | 64482189 |
| lg05 | 5A_72 | 29.424 | 5A | 64482306 |
| lg05 | 5A_71 | 29.429 | 5A | 64482241 |
| lg05 | 5A_166 | 29.702 | IWGSC_CSS_5AS_scaff_1538678 | 3433 |
| lg05 | 5A_190 | 30.167 | IWGSC_CSS_5AS_scaff_1552773 | 632 |
| lg05 | 5A_46 | 31.108 | 5A | 27677773 |
| lg05 | 5A_63 | 33.274 | 5A | 59985169 |
| lg05 | 5A_195 | 36.198 | IWGSC_CSS_5AS_scaff_1535834 | 9950 |
| lg05 | 5A_194 | 36.198 | IWGSC_CSS_5AS_scaff_1535834 | 9926 |
| lg05 | 5A_167 | 37.667 | IWGSC_CSS_5AS_scaff_1538678 | 3447 |
| lg05 | 5A_68 | 37.995 | 5A | 64482153 |
| lg05 | 5A_227 | 38.133 | IWGSC_CSS_5AS_scaff_1519270 | 1150 |
| lg05 | 5A_69 | 38.141 | 5A | 64482161 |
| lg05 | 5A_40 | 38.364 | 5A | 19697999 |
| lg05 | 5A_41 | 38.499 | 5A | 19698013 |
| lg05 | 5A_226 | 38.87 | IWGSC_CSS_5AS_scaff_1519270 | 1080 |
| lg05 | 5A_44 | 39.98 | 5A | 26603935 |
| lg05 | 5A_28 | 40.794 | IWGSC_CSS_5AS_scaff_1550617 | 3524 |
| lg05 | 5A_168 | 41.567 | IWGSC_CSS_5AS_scaff_1505066 | 2443 |
| lg05 | 5A_22 | 42.27 | IWGSC_CSS_5AS_scaff_1533335 | 2131 |
| lg05 | 5A_23 | 42.27 | IWGSC_CSS_5AS_scaff_1533335 | 2133 |
| lg05 | 5A_192 | 42.541 | IWGSC_CSS_5AS_scaff_1552691 | 2857 |
| lg05 | 5A_208 | 43.635 | IWGSC_CSS_5AS_scaff_1543778 | 3011 |
| lg05 | 5A_209 | 43.68 | IWGSC_CSS_5AS_scaff_1543778 | 3075 |
| lg05 | 5A_207 | 43.738 | IWGSC_CSS_5AS_scaff_1543778 | 2965 |
| lg05 | 5A_160 | 44.592 | IWGSC_CSS_5AS_scaff_1471219 | 5497 |
| lg05 | 5A_184 | 44.672 | IWGSC_CSS_5AS_scaff_1552240 | 2735 |
| lg05 | 5A_43 | 44.735 | 5A | 26404280 |
| lg05 | 5A_55 | 45.296 | 5A | 46369994 |
| lg05 | 5A_164 | 45.544 | IWGSC_CSS_5AS_scaff_1534143 | 1529 |
| lg05 | 5A_12 | 46.155 | IWGSC_CSS_5AS_scaff_1477412 | 15623 |
| lg05 | 5A_204 | 46.989 | IWGSC_CSS_5AS_scaff_1524153 | 4566 |
| lg05 | 5A_42 | 47.258 | 5A | 26404279 |
| lg05 | 5A_17 | 48.418 | IWGSC_CSS_5AS_scaff_1540844 | 869 |
| lg05 | 5A_47 | 50.042 | 5A | 28283104 |
| lg05 | 5A_188 | 51.429 | IWGSC_CSS_5AS_scaff_1474573 | 2880 |
| lg05 | 5A_58 | 51.855 | 5A | 49601322 |
| lg05 | 5A_185 | 51.893 | IWGSC_CSS_5AS_scaff_1552240 | 2777 |
| lg05 | 5A_187 | 51.893 | IWGSC_CSS_5AS_scaff_1552240 | 2936 |
| lg05 | 5A_193 | 51.893 | IWGSC_CSS_5AS_scaff_1552691 | 2859 |
| lg05 | 5A_186 | 51.893 | IWGSC_CSS_5AS_scaff_1552240 | 2829 |
| lg05 | 5A_176 | 51.933 | IWGSC_CSS_5AS_scaff_1505285 | 2625 |
| lg05 | 5A_203 | 52.466 | IWGSC_CSS_5AS_scaff_1535197 | 5902 |
| lg05 | 5A_26 | 53.059 | IWGSC_CSS_5AS_scaff_1549111 | 3943 |
| lg05 | 5A_183 | 53.24 | IWGSC_CSS_5AS_scaff_1481686 | 2980 |
| lg05 | 5A_6 | 53.445 | IWGSC_CSS_5AS_scaff_1510502 | 2264 |
| lg05 | 5A_202 | 53.475 | IWGSC_CSS_5AS_scaff_1535197 | 5893 |
| lg05 | 5A_212 | 54.498 | IWGSC_CSS_5AS_scaff_1534606 | 6655 |
| lg05 | 5A_213 | 54.57 | IWGSC_CSS_5AS_scaff_1534606 | 6769 |
| lg05 | 5A_211 | 54.588 | IWGSC_CSS_5AS_scaff_1534606 | 6648 |
| lg05 | 5A_196 | 55.777 | IWGSC_CSS_5AS_scaff_1538270 | 5633 |
| lg05 | 5A_200 | 56.889 | IWGSC_CSS_5AS_scaff_1540810 | 3408 |
| lg05 | 5A_162 | 58.407 | IWGSC_CSS_5AS_scaff_1526262 | 1925 |
| lg05 | 5A_169 | 59.65 | IWGSC_CSS_5AS_scaff_1510322 | 3178 |
| lg05 | 5A_18 | 59.908 | IWGSC_CSS_5AS_scaff_1549408 | 11256 |
| lg05 | 5A_20 | 59.983 | IWGSC_CSS_5AS_scaff_1549408 | 11337 |
| lg05 | 5A_189 | 59.991 | IWGSC_CSS_5AS_scaff_1474573 | 2891 |
| lg05 | 5A_21 | 59.991 | IWGSC_CSS_5AS_scaff_1549408 | 11386 |
| lg05 | 5A_19 | 59.996 | IWGSC_CSS_5AS_scaff_1549408 | 11311 |
| lg05 | 5A_57 | 60.076 | 5A | 49601154 |
| lg05 | 5A_59 | 60.128 | 5A | 49601335 |
| lg05 | 5A_163 | 61.77 | IWGSC_CSS_5AS_scaff_1526262 | 1934 |
| lg05 | 5A_45 | 63.471 | 5A | 27464929 |
| lg05 | 5A_191 | 64.209 | IWGSC_CSS_5AS_scaff_1552691 | 2757 |
| lg05 | 5A_61 | 64.532 | 5A | 58191835 |
| lg05 | 5A_159 | 64.638 | IWGSC_CSS_5AS_scaff_1471219 | 5488 |
| lg05 | 5A_60 | 65.752 | 5A | 53113600 |
| lg05 | 5A_37 | 69.118 | 5A | 18212239 |
| lg05 | 5A_51 | 69.379 | 5A | 38315985 |
| lg05 | 5A_53 | 69.451 | 5A | 38316061 |
| lg05 | 5A_52 | 69.451 | 5A | 38316059 |
| lg05 | 5A_50 | 69.556 | 5A | 38315924 |
| lg05 | 5A_38 | 69.757 | 5A | 18212244 |
| lg05 | 5A_36 | 69.757 | 5A | 18212209 |
| lg05 | 5A_39 | 70.563 | 5A | 18227324 |
| lg05 | 5A_179 | 72.311 | IWGSC_CSS_5AL_scaff_2781486 | 2809 |
| lg05 | 5A_177 | 72.311 | IWGSC_CSS_5AL_scaff_2781486 | 2610 |
| lg05 | 5A_178 | 72.311 | IWGSC_CSS_5AL_scaff_2781486 | 2647 |
| lg05 | 5A_8 | 73.86 | IWGSC_CSS_5AL_scaff_2811947 | 2644 |
| lg05 | 5A_214 | 75.776 | IWGSC_CSS_5AL_scaff_2795556 | 1651 |
| lg05 | 5A_27 | 77.392 | IWGSC_CSS_5AL_scaff_2732974 | 567 |
| lg05 | 5A_30 | 78.601 | IWGSC_CSS_5AL_scaff_2767266 | 2387 |
| lg05 | 5A_32 | 78.619 | IWGSC_CSS_5AL_scaff_2767266 | 2423 |
| lg05 | 5A_29 | 78.626 | IWGSC_CSS_5AL_scaff_2767266 | 2353 |
| lg05 | 5A_33 | 78.659 | IWGSC_CSS_5AL_scaff_2767266 | 2440 |
| lg05 | 5A_31 | 78.671 | IWGSC_CSS_5AL_scaff_2767266 | 2418 |
| lg05 | 5A_34 | 78.761 | IWGSC_CSS_5AL_scaff_2767266 | 10969 |
| lg05 | 5A_25 | 80.256 | IWGSC_CSS_5AL_scaff_2783478 | 12634 |
| lg05 | 5A_217 | 80.979 | IWGSC_CSS_5AL_scaff_2731998 | 1040 |
| lg05 | 5A_218 | 80.994 | IWGSC_CSS_5AL_scaff_2731998 | 1071 |
| lg05 | 5A_67 | 85.19 | 5A | 62584383 |
| lg05 | 5A_62 | 85.453 | 5A | 59692862 |
| lg05 | 5A_76 | 89.343 | 5A | 65932043 |
| lg05 | 5A_79 | 92.41 | 5A | 66104094 |
| lg05 | 5A_78 | 93.011 | 5A | 66058304 |
| lg05 | 5A_75 | 93.282 | 5A | 65765142 |
| lg05 | 5A_77 | 93.744 | 5A | 66058303 |
| lg05 | 5A_80 | 93.822 | 5A | 66148673 |
| lg05 | 5A_87 | 94.819 | 5A | 71263696 |
| lg05 | 5A_88 | 94.819 | 5A | 71263714 |
| lg05 | 5A_86 | 94.819 | 5A | 71263694 |
| lg05 | 5A_84 | 96.19 | 5A | 68578284 |
| lg05 | 5A_73 | 100.504 | 5A | 65560228 |
| lg05 | 5A_74 | 100.504 | 5A | 65560325 |
| lg05 | 5A_173 | 106.05 | IWGSC_CSS_5AL_scaff_2767073 | 3202 |
| lg05 | 5A_175 | 106.05 | IWGSC_CSS_5AL_scaff_2767073 | 3207 |
| lg05 | 5A_172 | 106.05 | IWGSC_CSS_5AL_scaff_2767073 | 3138 |
| lg05 | 5A_171 | 106.05 | IWGSC_CSS_5AL_scaff_2767073 | 3109 |
| lg05 | 5A_174 | 106.05 | IWGSC_CSS_5AL_scaff_2767073 | 3204 |
| lg05 | 5A_85 | 111.432 | 5A | 70046381 |
| lg05 | 5A_82 | 112.363 | 5A | 67061754 |
| lg05 | 5A_83 | 112.368 | 5A | 67061842 |
| lg05 | 5A_81 | 112.371 | 5A | 67061747 |
| lg05 | 5A_89 | 115.297 | 5A | 74700182 |
| lg05 | 5A_91 | 120.858 | 5A | 76866801 |
| lg05 | 5A_92 | 120.858 | 5A | 76866802 |
| lg05 | 5A_90 | 122.021 | 5A | 76168632 |
| lg05 | 5A_103 | 122.825 | 5A | 80307443 |
| lg05 | 5A_100 | 122.87 | 5A | 80307333 |
| lg05 | 5A_104 | 122.87 | 5A | 80307449 |
| lg05 | 5A_101 | 122.87 | 5A | 80307423 |
| lg05 | 5A_102 | 122.87 | 5A | 80307441 |
| lg05 | 5A_99 | 122.87 | 5A | 80307309 |
| lg05 | 5A_105 | 122.878 | 5A | 80307463 |
| lg05 | 5A_111 | 136.096 | 5A | 90810267 |
| lg05 | 5A_108 | 138.776 | 5A | 83140818 |
| lg05 | 5A_109 | 140.99 | 5A | 83884349 |
| lg05 | 5A_107 | 141.748 | 5A | 81585211 |
| lg05 | 5A_112 | 142.95 | 5A | 90903415 |
| lg05 | 5A_110 | 143.389 | 5A | 86720619 |
| lg05 | 5A_106 | 145.088 | 5A | 81249492 |
| lg05 | 5A_94 | 146.564 | 5A | 79420651 |
| lg05 | 5A_95 | 146.564 | 5A | 79420663 |
| lg05 | 5A_96 | 146.564 | 5A | 79420674 |
| lg05 | 5A_93 | 146.579 | 5A | 79420581 |
| lg05 | 5A_97 | 146.624 | 5A | 79420716 |
| lg05 | 5A_98 | 146.649 | 5A | 79420755 |
| lg05 | 5A_1 | 148.361 | IWGSC_CSS_5AL_scaff_2792699 | 5552 |
| lg12 | 5B_23 | 0 | IWGSC_CSS_5BS_scaff_2288060 | 2180 |
| lg12 | 5B_24 | 1.16 | IWGSC_CSS_5BS_scaff_2288060 | 2525 |
| lg12 | 5B_6 | 10.944 | IWGSC_CSS_5BS_scaff_2241026 | 2605 |
| lg12 | 5B_5 | 11.002 | IWGSC_CSS_5BS_scaff_2241026 | 2510 |
| lg12 | 5B_8 | 11.002 | IWGSC_CSS_5BS_scaff_2241026 | 2654 |
| lg12 | 5B_16 | 11.888 | IWGSC_CSS_5BS_scaff_2274983 | 3547 |
| lg12 | 5B_15 | 11.891 | IWGSC_CSS_5BS_scaff_2274983 | 3536 |
| lg12 | 5B_17 | 11.9 | IWGSC_CSS_5BS_scaff_2274983 | 3553 |
| lg12 | 5B_14 | 12.752 | IWGSC_CSS_5BL_scaff_10825762 | 3994 |
| lg12 | 5B_11 | 12.956 | IWGSC_CSS_5BS_scaff_2252280 | 3370 |
| lg12 | 5B_28 | 13.038 | IWGSC_CSS_5BS_scaff_2244676 | 5101 |
| lg12 | 5B_13 | 13.182 | IWGSC_CSS_5BS_scaff_2246861 | 2818 |
| lg12 | 5B_39 | 13.249 | IWGSC_CSS_5BS_scaff_2242166 | 1917 |
| lg12 | 5B_21 | 13.332 | IWGSC_CSS_5BS_scaff_2282267 | 865 |
| lg12 | 5B_22 | 13.373 | IWGSC_CSS_5BS_scaff_2284917 | 1976 |
| lg12 | 5B_20 | 13.549 | IWGSC_CSS_5BS_scaff_2288883 | 1157 |
| lg12 | 5B_30 | 13.79 | IWGSC_CSS_5BL_scaff_10845242 | 14616 |
| lg12 | 5B_32 | 13.809 | IWGSC_CSS_5BL_scaff_10845242 | 14624 |
| lg12 | 5B_29 | 13.819 | IWGSC_CSS_5BL_scaff_10845242 | 14597 |
| lg12 | 5B_31 | 13.82 | IWGSC_CSS_5BL_scaff_10845242 | 14623 |
| lg12 | 5B_33 | 13.82 | IWGSC_CSS_5BL_scaff_10845242 | 14635 |
| lg12 | 5B_43 | 14.045 | IWGSC_CSS_5BL_scaff_10907461 | 9937 |
| lg12 | 5B_7 | 14.271 | IWGSC_CSS_5BS_scaff_2241026 | 2653 |
| lg12 | 5B_27 | 16.365 | IWGSC_CSS_5BS_scaff_2265335 | 1150 |
| lg12 | 5B_38 | 19.257 | IWGSC_CSS_5BL_scaff_10918325 | 2886 |
| lg12 | 5B_4 | 22.616 | IWGSC_CSS_5BL_scaff_10808685 | 3302 |
| lg12 | 5B_41 | 23.032 | IWGSC_CSS_5BL_scaff_10871343 | 2474 |
| lg12 | 5B_19 | 23.447 | IWGSC_CSS_5BL_scaff_10813184 | 1277 |
| lg12 | 5B_18 | 23.734 | IWGSC_CSS_5BL_scaff_10813184 | 1273 |
| lg12 | 5B_34 | 35.443 | IWGSC_CSS_5BL_scaff_10870033 | 2749 |
| lg12 | 5B_12 | 36.23 | IWGSC_CSS_5BL_scaff_10922378 | 4803 |
| lg12 | 5B_40 | 37.586 | IWGSC_CSS_5BL_scaff_10925284 | 2480 |
| lg19 | 5D_89 | 0 | 5D | 156097313 |
| lg19 | 5D_91 | 0.033 | 5D | 156097391 |
| lg19 | 5D_87 | 0.037 | 5D | 156097252 |
| lg19 | 5D_90 | 0.04 | 5D | 156097362 |
| lg19 | 5D_88 | 0.069 | 5D | 156097295 |
| lg19 | 5D_78 | 1.001 | 5D | 154645505 |
| lg19 | 5D_77 | 1.054 | 5D | 154645472 |
| lg19 | 5D_79 | 1.212 | 5D | 154645580 |
| lg19 | 5D_86 | 1.557 | 5D | 154648731 |
| lg19 | 5D_81 | 1.557 | 5D | 154648583 |
| lg19 | 5D_82 | 1.557 | 5D | 154648706 |
| lg19 | 5D_84 | 1.557 | 5D | 154648724 |
| lg19 | 5D_85 | 1.559 | 5D | 154648730 |
| lg19 | 5D_83 | 1.561 | 5D | 154648707 |
| lg19 | 5D_80 | 1.563 | 5D | 154648539 |
| lg19 | 5D_102 | 13.382 | IWGSC_CSS_5DL_scaff_4536817 | 1668 |
| lg19 | 5D_41 | 18.533 | 5D | 130431392 |
| lg19 | 5D_67 | 25.809 | 5D | 152910904 |
| lg19 | 5D_66 | 25.892 | 5D | 152910843 |
| lg19 | 5D_68 | 26.384 | 5D | 152913953 |
| lg19 | 5D_101 | 26.603 | IWGSC_CSS_5DL_scaff_4604042 | 1616 |
| lg19 | 5D_75 | 26.785 | 5D | 152922565 |
| lg19 | 5D_72 | 26.785 | 5D | 152922413 |
| lg19 | 5D_70 | 26.785 | 5D | 152922388 |
| lg19 | 5D_73 | 26.785 | 5D | 152922418 |
| lg19 | 5D_71 | 26.785 | 5D | 152922389 |
| lg19 | 5D_74 | 26.787 | 5D | 152922544 |
| lg19 | 5D_69 | 26.787 | 5D | 152922365 |
| lg19 | 5D_63 | 26.836 | 5D | 150380558 |
| lg19 | 5D_64 | 26.836 | 5D | 150380571 |
| lg19 | 5D_76 | 26.874 | 5D | 152922756 |
| lg19 | 5D_112 | 27.348 | IWGSC_CSS_5DL_scaff_4608677 | 2124 |
| lg19 | 5D_113 | 27.348 | IWGSC_CSS_5DL_scaff_4608677 | 2137 |
| lg19 | 5D_65 | 27.728 | 5D | 152910807 |
| lg19 | 5D_1 | 27.749 | IWGSC_CSS_5DL_scaff_4607437 | 3196 |
| lg19 | 5D_42 | 30.752 | 5D | 131465646 |
| lg19 | 5D_4 | 36.54 | IWGSC_CSS_5DL_scaff_4491546 | 11 |
| lg19 | 5D_43 | 39.874 | 5D | 137938058 |
| lg19 | 5D_62 | 47.603 | 5D | 147969364 |
| lg19 | 5D_44 | 51.114 | 5D | 139939058 |
| lg19 | 5D_53 | 54.865 | 5D | 143381123 |
| lg19 | 5D_61 | 55.846 | 5D | 146696221 |
| lg19 | 5D_39 | 56.431 | 5D | 114218159 |
| lg19 | 5D_54 | 58.698 | 5D | 144104486 |
| lg19 | 5D_46 | 59.27 | 5D | 141493881 |
| lg19 | 5D_45 | 59.275 | 5D | 141493877 |
| lg19 | 5D_47 | 59.291 | 5D | 141493890 |
| lg19 | 5D_52 | 59.721 | 5D | 143205651 |
| lg19 | 5D_48 | 59.969 | 5D | 141895371 |
| lg19 | 5D_50 | 60.924 | 5D | 142014854 |
| lg19 | 5D_49 | 60.978 | 5D | 142014762 |
| lg19 | 5D_40 | 61.268 | 5D | 115669362 |
| lg19 | 5D_55 | 61.87 | 5D | 146014392 |
| lg19 | 5D_57 | 61.873 | 5D | 146014408 |
| lg19 | 5D_60 | 61.884 | 5D | 146014623 |
| lg19 | 5D_58 | 61.886 | 5D | 146014538 |
| lg19 | 5D_56 | 61.889 | 5D | 146014394 |
| lg19 | 5D_59 | 61.892 | 5D | 146014547 |
| lg19 | 5D_36 | 62.736 | 5D | 111256002 |
| lg19 | 5D_51 | 63.06 | 5D | 142014950 |
| lg19 | 5D_107 | 65.181 | IWGSC_CSS_5DL_scaff_4569775 | 3077 |
| lg19 | 5D_35 | 70.39 | 5D | 109064709 |
| lg19 | 5D_33 | 74.687 | 5D | 105487289 |
| lg19 | 5D_34 | 77.008 | 5D | 106270114 |
| lg19 | 5D_32 | 86.207 | 5D | 103837020 |
| lg19 | 5D_14 | 97.007 | 5D | 30421196 |
| lg19 | 5D_24 | 105.051 | 5D | 66757627 |
| lg19 | 5D_21 | 107.68 | 5D | 49625505 |
| lg19 | 5D_13 | 108.972 | 5D | 28460305 |
| lg19 | 5D_17 | 109.305 | 5D | 37129441 |
| lg19 | 5D_120 | 109.523 | IWGSC_CSS_5DS_scaff_2738942 | 2727 |
| lg19 | 5D_97 | 109.814 | IWGSC_CSS_5DL_scaff_4564381 | 1520 |
| lg19 | 5D_27 | 109.919 | 5D | 81469975 |
| lg19 | 5D_114 | 110.068 | IWGSC_CSS_5DL_scaff_4523697 | 1197 |
| lg19 | 5D_115 | 110.248 | IWGSC_CSS_5DS_scaff_2762772 | 4325 |
| lg19 | 5D_23 | 110.391 | 5D | 63665537 |
| lg19 | 5D_5 | 110.423 | IWGSC_CSS_5DS_scaff_2772588 | 1439 |
| lg19 | 5D_29 | 110.442 | 5D | 86634206 |
| lg19 | 5D_94 | 110.536 | IWGSC_CSS_5DL_scaff_4523313 | 4257 |
| lg19 | 5D_121 | 110.594 | IWGSC_CSS_5DS_scaff_2729867 | 2010 |
| lg19 | 5D_31 | 110.731 | 5D | 99597745 |
| lg19 | 5D_25 | 110.801 | 5D | 75480277 |
| lg19 | 5D_28 | 110.929 | 5D | 85887780 |
| lg19 | 5D_93 | 111.171 | IWGSC_CSS_5DL_scaff_4584488 | 7266 |
| lg19 | 5D_30 | 111.416 | 5D | 87690313 |
| lg19 | 5D_116 | 111.525 | IWGSC_CSS_5DS_scaff_2755873 | 4097 |
| lg19 | 5D_15 | 111.605 | 5D | 33581740 |
| lg19 | 5D_118 | 112.137 | IWGSC_CSS_5DL_scaff_4572167 | 430 |
| lg19 | 5D_106 | 112.536 | IWGSC_CSS_5DS_scaff_2731185 | 558 |
| lg19 | 5D_2 | 113.08 | IWGSC_CSS_5DS_scaff_2753681 | 2722 |
| lg19 | 5D_119 | 113.411 | IWGSC_CSS_5DS_scaff_2737848 | 1418 |
| lg19 | 5D_19 | 113.812 | 5D | 39078010 |
| lg19 | 5D_18 | 115.467 | 5D | 38465395 |
| lg19 | 5D_16 | 115.909 | 5D | 33687362 |
| lg19 | 5D_100 | 116.207 | IWGSC_CSS_5DS_scaff_2763043 | 4842 |
| lg19 | 5D_12 | 118.435 | 5D | 22131911 |
| lg19 | 5D_3 | 120.233 | IWGSC_CSS_5DS_scaff_1659958 | 3447 |
| lg19 | 5D_11 | 127.376 | 5D | 17446246 |
| lg19 | 5D_96 | 129.434 | IWGSC_CSS_5DS_scaff_2730721 | 1700 |
| lg19 | 5D_10 | 131.015 | 5D | 15763285 |
| lg19 | 5D_95 | 135.313 | IWGSC_CSS_5DS_scaff_2743836 | 668 |
| lg19 | 5D_6 | 136.243 | 5D | 6514822 |
| lg06 | 6A_121 | 0 | IWGSC_CSS_6AL_scaff_5772476 | 2171 |
| lg06 | 6A_8 | 1.405 | 6A | 2119702 |
| lg06 | 6A_1 | 1.466 | IWGSC_CSS_6AL_scaff_5775286 | 819 |
| lg06 | 6A_97 | 2.027 | 6A | 194956347 |
| lg06 | 6A_96 | 2.531 | 6A | 192959562 |
| lg06 | 6A_94 | 2.552 | 6A | 192959367 |
| lg06 | 6A_55 | 3.916 | 6A | 165435382 |
| lg06 | 6A_58 | 4.108 | 6A | 180461944 |
| lg06 | 6A_4 | 4.583 | 6A | 466892 |
| lg06 | 6A_7 | 4.586 | 6A | 466928 |
| lg06 | 6A_6 | 4.587 | 6A | 466922 |
| lg06 | 6A_5 | 4.592 | 6A | 466921 |
| lg06 | 6A_3 | 4.598 | 6A | 466815 |
| lg06 | 6A_113 | 4.635 | IWGSC_CSS_6AL_scaff_5829643 | 663 |
| lg06 | 6A_64 | 4.724 | 6A | 182335430 |
| lg06 | 6A_74 | 4.852 | 6A | 187664703 |
| lg06 | 6A_67 | 4.964 | 6A | 183693188 |
| lg06 | 6A_62 | 5.045 | 6A | 181750733 |
| lg06 | 6A_63 | 5.06 | 6A | 181750735 |
| lg06 | 6A_72 | 5.176 | 6A | 185606674 |
| lg06 | 6A_75 | 5.224 | 6A | 187664713 |
| lg06 | 6A_66 | 5.275 | 6A | 182806872 |
| lg06 | 6A_70 | 5.311 | 6A | 184363121 |
| lg06 | 6A_69 | 5.4 | 6A | 184363117 |
| lg06 | 6A_52 | 5.559 | 6A | 162833386 |
| lg06 | 6A_73 | 5.919 | 6A | 187234358 |
| lg06 | 6A_79 | 5.958 | 6A | 190311776 |
| lg06 | 6A_77 | 5.974 | 6A | 190311769 |
| lg06 | 6A_82 | 5.976 | 6A | 190311892 |
| lg06 | 6A_78 | 5.977 | 6A | 190311775 |
| lg06 | 6A_83 | 5.978 | 6A | 190311899 |
| lg06 | 6A_80 | 5.979 | 6A | 190311781 |
| lg06 | 6A_85 | 5.979 | 6A | 190311952 |
| lg06 | 6A_76 | 5.979 | 6A | 190311763 |
| lg06 | 6A_84 | 5.983 | 6A | 190311916 |
| lg06 | 6A_71 | 5.995 | 6A | 185365111 |
| lg06 | 6A_81 | 6.034 | 6A | 190311862 |
| lg06 | 6A_86 | 6.469 | 6A | 190414886 |
| lg06 | 6A_53 | 6.504 | 6A | 163587033 |
| lg06 | 6A_92 | 6.556 | 6A | 190659407 |
| lg06 | 6A_90 | 6.557 | 6A | 190659342 |
| lg06 | 6A_91 | 6.557 | 6A | 190659348 |
| lg06 | 6A_88 | 6.557 | 6A | 190659217 |
| lg06 | 6A_87 | 6.557 | 6A | 190659206 |
| lg06 | 6A_89 | 6.557 | 6A | 190659245 |
| lg06 | 6A_93 | 6.686 | 6A | 190659414 |
| lg06 | 6A_68 | 6.913 | 6A | 183739680 |
| lg06 | 6A_61 | 7.065 | 6A | 181418791 |
| lg06 | 6A_65 | 7.222 | 6A | 182335445 |
| lg06 | 6A_60 | 7.352 | 6A | 181327965 |
| lg06 | 6A_59 | 7.426 | 6A | 181327958 |
| lg06 | 6A_95 | 8.698 | 6A | 192959377 |
| lg06 | 6A_51 | 9.442 | 6A | 154007625 |
| lg06 | 6A_50 | 9.543 | 6A | 154007431 |
| lg06 | 6A_9 | 10.542 | 6A | 3586699 |
| lg06 | 6A_57 | 13.061 | 6A | 179711807 |
| lg06 | 6A_54 | 13.211 | 6A | 164015832 |
| lg06 | 6A_117 | 15.473 | IWGSC_CSS_6AL_scaff_5798469 | 3616 |
| lg06 | 6A_11 | 16.082 | 6A | 4214072 |
| lg06 | 6A_10 | 16.135 | 6A | 4214055 |
| lg06 | 6A_47 | 16.249 | 6A | 81248321 |
| lg06 | 6A_12 | 16.358 | 6A | 4214078 |
| lg06 | 6A_114 | 17.061 | IWGSC_CSS_6AL_scaff_5763373 | 745 |
| lg06 | 6A_49 | 17.316 | 6A | 147260337 |
| lg06 | 6A_46 | 18.001 | 6A | 44974235 |
| lg06 | 6A_48 | 18.176 | 6A | 128855175 |
| lg06 | 6A_2 | 19.164 | IWGSC_CSS_6AS_scaff_4343520 | 3617 |
| lg06 | 6A_45 | 29.58 | 6A | 22026839 |
| lg06 | 6A_116 | 30.47 | IWGSC_CSS_6AS_scaff_4373717 | 17471 |
| lg06 | 6A_115 | 30.543 | IWGSC_CSS_6AS_scaff_4373717 | 17454 |
| lg06 | 6A_43 | 31.448 | 6A | 19173341 |
| lg06 | 6A_42 | 31.682 | 6A | 19173242 |
| lg06 | 6A_44 | 31.94 | 6A | 19173463 |
| lg06 | 6A_35 | 38.719 | 6A | 11456065 |
| lg06 | 6A_36 | 38.993 | 6A | 11456066 |
| lg06 | 6A_18 | 39.738 | 6A | 6535483 |
| lg06 | 6A_15 | 39.761 | 6A | 6535438 |
| lg06 | 6A_17 | 39.791 | 6A | 6535472 |
| lg06 | 6A_13 | 39.799 | 6A | 6535322 |
| lg06 | 6A_19 | 39.846 | 6A | 6535506 |
| lg06 | 6A_16 | 39.905 | 6A | 6535459 |
| lg06 | 6A_14 | 39.923 | 6A | 6535341 |
| lg06 | 6A_119 | 40.94 | IWGSC_CSS_6AS_scaff_4428587 | 1905 |
| lg06 | 6A_40 | 41.362 | 6A | 14199610 |
| lg06 | 6A_41 | 41.363 | 6A | 14199641 |
| lg06 | 6A_118 | 41.82 | IWGSC_CSS_6AS_scaff_4428587 | 1831 |
| lg06 | 6A_38 | 42.84 | 6A | 12163689 |
| lg06 | 6A_37 | 42.92 | 6A | 12163674 |
| lg06 | 6A_39 | 48.592 | 6A | 12974685 |
| lg06 | 6A_22 | 63.355 | 6A | 9571332 |
| lg06 | 6A_34 | 63.717 | 6A | 9571459 |
| lg06 | 6A_32 | 63.717 | 6A | 9571454 |
| lg06 | 6A_29 | 63.718 | 6A | 9571443 |
| lg06 | 6A_21 | 63.718 | 6A | 9571330 |
| lg06 | 6A_33 | 63.773 | 6A | 9571458 |
| lg06 | 6A_31 | 63.773 | 6A | 9571451 |
| lg06 | 6A_28 | 63.776 | 6A | 9571431 |
| lg06 | 6A_30 | 63.776 | 6A | 9571445 |
| lg06 | 6A_27 | 63.776 | 6A | 9571430 |
| lg06 | 6A_26 | 63.803 | 6A | 9571415 |
| lg06 | 6A_24 | 63.826 | 6A | 9571357 |
| lg06 | 6A_20 | 63.826 | 6A | 9571315 |
| lg06 | 6A_25 | 63.826 | 6A | 9571368 |
| lg06 | 6A_23 | 63.849 | 6A | 9571336 |
| lg13 | 6B_254 | 0 | 6B | 199960622 |
| lg13 | 6B_253 | 7.582 | 6B | 197245774 |
| lg13 | 6B_252 | 7.592 | 6B | 197245765 |
| lg13 | 6B_250 | 11.161 | 6B | 193579649 |
| lg13 | 6B_251 | 11.166 | 6B | 193579705 |
| lg13 | 6B_248 | 16.839 | 6B | 192169915 |
| lg13 | 6B_249 | 16.841 | 6B | 192169943 |
| lg13 | 6B_247 | 22.651 | 6B | 192034688 |
| lg13 | 6B_246 | 23.766 | 6B | 189632551 |
| lg13 | 6B_232 | 35.581 | 6B | 182459592 |
| lg13 | 6B_239 | 36.27 | 6B | 183046099 |
| lg13 | 6B_236 | 37.393 | 6B | 182890131 |
| lg13 | 6B_240 | 37.933 | 6B | 184035562 |
| lg13 | 6B_234 | 38.259 | 6B | 182889997 |
| lg13 | 6B_235 | 38.259 | 6B | 182890065 |
| lg13 | 6B_233 | 38.264 | 6B | 182889963 |
| lg13 | 6B_243 | 39.172 | 6B | 184515855 |
| lg13 | 6B_244 | 39.172 | 6B | 184515863 |
| lg13 | 6B_242 | 39.172 | 6B | 184515710 |
| lg13 | 6B_241 | 39.172 | 6B | 184515683 |
| lg13 | 6B_238 | 40.523 | 6B | 182931356 |
| lg13 | 6B_237 | 40.538 | 6B | 182931323 |
| lg13 | 6B_245 | 44.175 | 6B | 186888773 |
| lg13 | 6B_231 | 47.994 | 6B | 179037959 |
| lg13 | 6B_230 | 48.012 | 6B | 179037936 |
| lg13 | 6B_223 | 50.785 | 6B | 174988543 |
| lg13 | 6B_222 | 50.787 | 6B | 174988542 |
| lg13 | 6B_17 | 52.779 | IWGSC_CSS_6BL_scaff_4391332 | 1573 |
| lg13 | 6B_16 | 52.794 | IWGSC_CSS_6BL_scaff_4391332 | 1453 |
| lg13 | 6B_8 | 53.312 | IWGSC_CSS_6BL_scaff_4401879 | 3728 |
| lg13 | 6B_209 | 53.791 | 6B | 147262357 |
| lg13 | 6B_220 | 54.616 | 6B | 174432016 |
| lg13 | 6B_228 | 54.959 | 6B | 177072114 |
| lg13 | 6B_227 | 55.132 | 6B | 177072018 |
| lg13 | 6B_224 | 55.607 | 6B | 177007848 |
| lg13 | 6B_226 | 55.609 | 6B | 177007851 |
| lg13 | 6B_225 | 55.614 | 6B | 177007850 |
| lg13 | 6B_221 | 57.729 | 6B | 174556664 |
| lg13 | 6B_218 | 59.544 | 6B | 166485599 |
| lg13 | 6B_216 | 59.544 | 6B | 166485553 |
| lg13 | 6B_219 | 59.544 | 6B | 166485719 |
| lg13 | 6B_217 | 59.566 | 6B | 166485597 |
| lg13 | 6B_210 | 60.319 | 6B | 154437600 |
| lg13 | 6B_211 | 60.364 | 6B | 154437618 |
| lg13 | 6B_286 | 62.299 | IWGSC_CSS_6BL_scaff_4369299 | 5792 |
| lg13 | 6B_285 | 63.004 | IWGSC_CSS_6BL_scaff_4347983 | 2662 |
| lg13 | 6B_215 | 64.586 | 6B | 166171892 |
| lg13 | 6B_213 | 66.075 | 6B | 157207909 |
| lg13 | 6B_212 | 66.088 | 6B | 157207908 |
| lg13 | 6B_267 | 67.572 | IWGSC_CSS_6BL_scaff_4274073 | 1404 |
| lg13 | 6B_204 | 67.58 | 6B | 145094809 |
| lg13 | 6B_150 | 67.652 | 6B | 69912535 |
| lg13 | 6B_280 | 68.16 | IWGSC_CSS_6BL_scaff_1036473 | 2269 |
| lg13 | 6B_208 | 68.192 | 6B | 146185896 |
| lg13 | 6B_207 | 68.197 | 6B | 146185811 |
| lg13 | 6B_281 | 68.197 | IWGSC_CSS_6BL_scaff_1036473 | 2303 |
| lg13 | 6B_206 | 68.197 | 6B | 146185698 |
| lg13 | 6B_282 | 68.2 | IWGSC_CSS_6BL_scaff_1036473 | 2333 |
| lg13 | 6B_279 | 68.212 | IWGSC_CSS_6BL_scaff_1036473 | 2207 |
| lg13 | 6B_214 | 68.478 | 6B | 165391110 |
| lg13 | 6B_205 | 68.699 | 6B | 145094910 |
| lg13 | 6B_270 | 68.699 | IWGSC_CSS_6BL_scaff_4274073 | 1426 |
| lg13 | 6B_202 | 68.699 | 6B | 145094780 |
| lg13 | 6B_268 | 68.699 | IWGSC_CSS_6BL_scaff_4274073 | 1413 |
| lg13 | 6B_149 | 68.704 | 6B | 69912519 |
| lg13 | 6B_18 | 69.201 | IWGSC_CSS_6BL_scaff_4400794 | 3196 |
| lg13 | 6B_229 | 69.645 | 6B | 177223192 |
| lg13 | 6B_203 | 70.052 | 6B | 145094808 |
| lg13 | 6B_10 | 70.572 | IWGSC_CSS_6BL_scaff_4400546 | 5400 |
| lg13 | 6B_9 | 70.773 | IWGSC_CSS_6BL_scaff_3470 | 219 |
| lg13 | 6B_109 | 71.051 | 6B | 39851035 |
| lg13 | 6B_263 | 71.503 | IWGSC_CSS_6BL_scaff_4334254 | 1416 |
| lg13 | 6B_13 | 72.32 | IWGSC_CSS_6BL_scaff_4401422 | 4925 |
| lg13 | 6B_12 | 72.342 | IWGSC_CSS_6BL_scaff_4401422 | 4914 |
| lg13 | 6B_269 | 73.098 | IWGSC_CSS_6BL_scaff_4274073 | 1415 |
| lg13 | 6B_126 | 75.356 | 6B | 54575964 |
| lg13 | 6B_112 | 76.038 | 6B | 42352954 |
| lg13 | 6B_114 | 76.76 | 6B | 42541944 |
| lg13 | 6B_156 | 76.81 | 6B | 74791872 |
| lg13 | 6B_188 | 77.938 | 6B | 119102394 |
| lg13 | 6B_187 | 78.309 | 6B | 119102227 |
| lg13 | 6B_201 | 79.065 | 6B | 135359770 |
| lg13 | 6B_125 | 79.892 | 6B | 54575927 |
| lg13 | 6B_117 | 80.668 | 6B | 45790538 |
| lg13 | 6B_124 | 81.401 | 6B | 54575849 |
| lg13 | 6B_127 | 81.491 | 6B | 54575995 |
| lg13 | 6B_119 | 82.795 | 6B | 53130095 |
| lg13 | 6B_198 | 83.823 | 6B | 130603442 |
| lg13 | 6B_142 | 84.424 | 6B | 62320260 |
| lg13 | 6B_179 | 84.6 | 6B | 103064624 |
| lg13 | 6B_129 | 86.544 | 6B | 56187352 |
| lg13 | 6B_128 | 86.916 | 6B | 56187298 |
| lg13 | 6B_168 | 87.692 | 6B | 86175175 |
| lg13 | 6B_130 | 88.29 | 6B | 56187449 |
| lg13 | 6B_132 | 89.566 | 6B | 58284886 |
| lg13 | 6B_57 | 89.973 | 6B | 25353897 |
| lg13 | 6B_197 | 90.44 | 6B | 130603326 |
| lg13 | 6B_151 | 90.495 | 6B | 70219653 |
| lg13 | 6B_283 | 90.733 | IWGSC_CSS_6BL_scaff_4282958 | 1344 |
| lg13 | 6B_171 | 91.578 | 6B | 87343455 |
| lg13 | 6B_169 | 91.673 | 6B | 87343397 |
| lg13 | 6B_170 | 91.681 | 6B | 87343419 |
| lg13 | 6B_172 | 91.758 | 6B | 87343487 |
| lg13 | 6B_158 | 92.478 | 6B | 77377428 |
| lg13 | 6B_121 | 92.659 | 6B | 53739489 |
| lg13 | 6B_157 | 93.09 | 6B | 74791914 |
| lg13 | 6B_152 | 93.608 | 6B | 70730994 |
| lg13 | 6B_115 | 94.121 | 6B | 42541975 |
| lg13 | 6B_287 | 94.618 | IWGSC_CSS_6BL_scaff_4375581 | 1454 |
| lg13 | 6B_176 | 95.184 | 6B | 94243033 |
| lg13 | 6B_256 | 95.334 | IWGSC_CSS_6BS_scaff_2967162 | 612 |
| lg13 | 6B_118 | 96.016 | 6B | 46906005 |
| lg13 | 6B_15 | 96.754 | IWGSC_CSS_6BL_scaff_4304739 | 3724 |
| lg13 | 6B_160 | 96.762 | 6B | 80862567 |
| lg13 | 6B_154 | 96.767 | 6B | 71356520 |
| lg13 | 6B_111 | 97.156 | 6B | 41577313 |
| lg13 | 6B_194 | 97.454 | 6B | 128371489 |
| lg13 | 6B_186 | 97.909 | 6B | 118302441 |
| lg13 | 6B_275 | 98.479 | IWGSC_CSS_6BS_scaff_2979597 | 128 |
| lg13 | 6B_284 | 99.495 | IWGSC_CSS_6BL_scaff_4274962 | 4319 |
| lg13 | 6B_153 | 99.785 | 6B | 71356444 |
| lg13 | 6B_146 | 99.906 | 6B | 66430756 |
| lg13 | 6B_123 | 100.626 | 6B | 54375169 |
| lg13 | 6B_181 | 102.928 | 6B | 109915210 |
| lg13 | 6B_173 | 104.761 | 6B | 88928051 |
| lg13 | 6B_195 | 105.216 | 6B | 128371539 |
| lg13 | 6B_175 | 105.888 | 6B | 91380437 |
| lg13 | 6B_110 | 106.74 | 6B | 39861019 |
| lg13 | 6B_189 | 107.957 | 6B | 119296435 |
| lg13 | 6B_155 | 108.675 | 6B | 74791856 |
| lg13 | 6B_116 | 108.705 | 6B | 42542016 |
| lg13 | 6B_113 | 108.707 | 6B | 42541908 |
| lg13 | 6B_271 | 109.626 | IWGSC_CSS_6BS_scaff_2949067 | 2027 |
| lg13 | 6B_273 | 109.626 | IWGSC_CSS_6BS_scaff_2949067 | 2199 |
| lg13 | 6B_274 | 109.633 | IWGSC_CSS_6BS_scaff_2949067 | 2236 |
| lg13 | 6B_272 | 109.643 | IWGSC_CSS_6BS_scaff_2949067 | 2190 |
| lg13 | 6B_196 | 110.899 | 6B | 129334852 |
| lg13 | 6B_141 | 111.465 | 6B | 61575598 |
| lg13 | 6B_145 | 112.19 | 6B | 65137148 |
| lg13 | 6B_134 | 115.964 | 6B | 59211232 |
| lg13 | 6B_133 | 116.813 | 6B | 59211194 |
| lg13 | 6B_178 | 118.901 | 6B | 99074626 |
| lg13 | 6B_159 | 120.19 | 6B | 79186488 |
| lg13 | 6B_147 | 121.211 | 6B | 68815609 |
| lg13 | 6B_148 | 121.386 | 6B | 68815648 |
| lg13 | 6B_183 | 121.534 | 6B | 115342881 |
| lg13 | 6B_182 | 121.534 | 6B | 115342870 |
| lg13 | 6B_131 | 121.539 | 6B | 57189115 |
| lg13 | 6B_167 | 122.155 | 6B | 85167139 |
| lg13 | 6B_161 | 123.339 | 6B | 83549850 |
| lg13 | 6B_139 | 124.316 | 6B | 61567015 |
| lg13 | 6B_138 | 124.316 | 6B | 61566972 |
| lg13 | 6B_140 | 124.398 | 6B | 61567089 |
| lg13 | 6B_174 | 125.999 | 6B | 89335345 |
| lg13 | 6B_137 | 126.325 | 6B | 59749203 |
| lg13 | 6B_108 | 126.342 | 6B | 38506372 |
| lg13 | 6B_135 | 126.342 | 6B | 59749129 |
| lg13 | 6B_136 | 126.342 | 6B | 59749161 |
| lg13 | 6B_166 | 126.678 | 6B | 83949457 |
| lg13 | 6B_164 | 127.348 | 6B | 83860243 |
| lg13 | 6B_165 | 127.348 | 6B | 83860275 |
| lg13 | 6B_163 | 127.348 | 6B | 83860183 |
| lg13 | 6B_162 | 127.348 | 6B | 83860171 |
| lg13 | 6B_180 | 128.478 | 6B | 109115908 |
| lg13 | 6B_120 | 128.867 | 6B | 53708328 |
| lg13 | 6B_122 | 129.118 | 6B | 53758443 |
| lg13 | 6B_177 | 130.087 | 6B | 96342380 |
| lg13 | 6B_143 | 131.68 | 6B | 63390672 |
| lg13 | 6B_144 | 131.68 | 6B | 63390832 |
| lg13 | 6B_14 | 134.476 | IWGSC_CSS_6BS_scaff_2938830 | 91 |
| lg13 | 6B_185 | 136.26 | 6B | 115931306 |
| lg13 | 6B_184 | 136.263 | 6B | 115931288 |
| lg13 | 6B_191 | 137.347 | 6B | 125010139 |
| lg13 | 6B_190 | 138.014 | 6B | 119848665 |
| lg13 | 6B_199 | 138.107 | 6B | 132673357 |
| lg13 | 6B_11 | 139.857 | IWGSC_CSS_6BS_scaff_2996025 | 2554 |
| lg13 | 6B_192 | 142.227 | 6B | 125723367 |
| lg13 | 6B_193 | 142.327 | 6B | 126064815 |
| lg13 | 6B_200 | 143.511 | 6B | 133446087 |
| lg13 | 6B_277 | 144.503 | IWGSC_CSS_6BS_scaff_3032260 | 2072 |
| lg13 | 6B_276 | 144.887 | IWGSC_CSS_6BS_scaff_3032260 | 2035 |
| lg13 | 6B_20 | 144.89 | IWGSC_CSS_6BS_scaff_2950653 | 6715 |
| lg13 | 6B_19 | 145.279 | IWGSC_CSS_6BS_scaff_2950653 | 6712 |
| lg13 | 6B_265 | 146.606 | IWGSC_CSS_6BS_scaff_3049234 | 4076 |
| lg13 | 6B_264 | 146.614 | IWGSC_CSS_6BS_scaff_3049234 | 4059 |
| lg13 | 6B_6 | 148.898 | IWGSC_CSS_6BS_scaff_2983593 | 2021 |
| lg13 | 6B_7 | 148.898 | IWGSC_CSS_6BS_scaff_2983593 | 2032 |
| lg13 | 6B_5 | 148.898 | IWGSC_CSS_6BS_scaff_2983593 | 1947 |
| lg13 | 6B_4 | 149.352 | IWGSC_CSS_6BS_scaff_2983593 | 589 |
| lg13 | 6B_2 | 149.4 | IWGSC_CSS_6BS_scaff_2983593 | 508 |
| lg13 | 6B_1 | 149.4 | IWGSC_CSS_6BS_scaff_2983593 | 489 |
| lg13 | 6B_3 | 149.402 | IWGSC_CSS_6BS_scaff_2983593 | 517 |
| lg13 | 6B_54 | 151.389 | 6B | 24338937 |
| lg13 | 6B_53 | 151.391 | 6B | 24338912 |
| lg13 | 6B_60 | 154.888 | 6B | 27163220 |
| lg13 | 6B_59 | 154.888 | 6B | 27163099 |
| lg13 | 6B_101 | 155.952 | 6B | 35040808 |
| lg13 | 6B_104 | 155.952 | 6B | 35040862 |
| lg13 | 6B_70 | 156.25 | 6B | 29451592 |
| lg13 | 6B_32 | 156.32 | 6B | 16801072 |
| lg13 | 6B_31 | 156.32 | 6B | 16800997 |
| lg13 | 6B_72 | 156.664 | 6B | 29451632 |
| lg13 | 6B_83 | 157.078 | 6B | 35038337 |
| lg13 | 6B_87 | 157.078 | 6B | 35038437 |
| lg13 | 6B_69 | 157.254 | 6B | 29451499 |
| lg13 | 6B_39 | 158.091 | 6B | 16802705 |
| lg13 | 6B_46 | 158.091 | 6B | 16802841 |
| lg13 | 6B_48 | 158.091 | 6B | 16802850 |
| lg13 | 6B_42 | 158.091 | 6B | 16802775 |
| lg13 | 6B_45 | 158.091 | 6B | 16802837 |
| lg13 | 6B_47 | 158.091 | 6B | 16802848 |
| lg13 | 6B_35 | 158.253 | 6B | 16802673 |
| lg13 | 6B_37 | 158.341 | 6B | 16802677 |
| lg13 | 6B_44 | 158.341 | 6B | 16802830 |
| lg13 | 6B_38 | 158.849 | 6B | 16802687 |
| lg13 | 6B_49 | 159.536 | 6B | 16802853 |
| lg13 | 6B_36 | 159.849 | 6B | 16802676 |
| lg13 | 6B_34 | 160.314 | 6B | 16802670 |
| lg13 | 6B_43 | 160.331 | 6B | 16802783 |
| lg13 | 6B_33 | 160.351 | 6B | 16802652 |
| lg13 | 6B_41 | 160.351 | 6B | 16802765 |
| lg13 | 6B_40 | 160.351 | 6B | 16802741 |
| lg13 | 6B_68 | 161.965 | 6B | 29451403 |
| lg13 | 6B_99 | 162.127 | 6B | 35040743 |
| lg13 | 6B_102 | 162.127 | 6B | 35040818 |
| lg13 | 6B_76 | 162.524 | 6B | 31638334 |
| lg13 | 6B_52 | 162.845 | 6B | 23775241 |
| lg13 | 6B_58 | 162.945 | 6B | 27019767 |
| lg13 | 6B_278 | 164.149 | IWGSC_CSS_6BS_scaff_3000404 | 2025 |
| lg13 | 6B_50 | 165.374 | 6B | 18557816 |
| lg13 | 6B_55 | 166.221 | 6B | 24621382 |
| lg13 | 6B_56 | 166.256 | 6B | 24621407 |
| lg13 | 6B_62 | 167.223 | 6B | 27837235 |
| lg13 | 6B_67 | 167.343 | 6B | 27837368 |
| lg13 | 6B_64 | 167.436 | 6B | 27837272 |
| lg13 | 6B_61 | 167.453 | 6B | 27837197 |
| lg13 | 6B_65 | 167.484 | 6B | 27837333 |
| lg13 | 6B_100 | 167.948 | 6B | 35040748 |
| lg13 | 6B_71 | 168.367 | 6B | 29451631 |
| lg13 | 6B_91 | 168.56 | 6B | 35038502 |
| lg13 | 6B_255 | 170.078 | IWGSC_CSS_6BS_scaff_3048008 | 2851 |
| lg13 | 6B_30 | 170.742 | 6B | 16582103 |
| lg13 | 6B_80 | 171.24 | 6B | 33156219 |
| lg13 | 6B_75 | 171.245 | 6B | 30509545 |
| lg13 | 6B_78 | 171.747 | 6B | 31896113 |
| lg13 | 6B_79 | 171.747 | 6B | 31896155 |
| lg13 | 6B_77 | 171.747 | 6B | 31896022 |
| lg13 | 6B_97 | 173.524 | 6B | 35039989 |
| lg13 | 6B_84 | 173.529 | 6B | 35038341 |
| lg13 | 6B_73 | 173.529 | 6B | 29451636 |
| lg13 | 6B_89 | 173.529 | 6B | 35038479 |
| lg13 | 6B_81 | 173.529 | 6B | 35038315 |
| lg13 | 6B_82 | 173.529 | 6B | 35038335 |
| lg13 | 6B_85 | 173.529 | 6B | 35038366 |
| lg13 | 6B_90 | 173.529 | 6B | 35038482 |
| lg13 | 6B_93 | 173.621 | 6B | 35039918 |
| lg13 | 6B_92 | 173.696 | 6B | 35039890 |
| lg13 | 6B_94 | 173.779 | 6B | 35039945 |
| lg13 | 6B_95 | 173.779 | 6B | 35039961 |
| lg13 | 6B_96 | 173.879 | 6B | 35039984 |
| lg13 | 6B_88 | 174.029 | 6B | 35038473 |
| lg13 | 6B_86 | 174.029 | 6B | 35038431 |
| lg13 | 6B_74 | 174.102 | 6B | 29451638 |
| lg13 | 6B_98 | 174.45 | 6B | 35040703 |
| lg13 | 6B_106 | 174.463 | 6B | 35040915 |
| lg13 | 6B_105 | 174.478 | 6B | 35040898 |
| lg13 | 6B_103 | 174.508 | 6B | 35040861 |
| lg13 | 6B_66 | 174.937 | 6B | 27837363 |
| lg13 | 6B_63 | 174.972 | 6B | 27837242 |
| lg13 | 6B_51 | 176.315 | 6B | 21460251 |
| lg13 | 6B_107 | 176.619 | 6B | 36755718 |
| lg13 | 6B_29 | 180.097 | 6B | 12078486 |
| lg13 | 6B_28 | 180.441 | 6B | 12078474 |
| lg13 | 6B_27 | 190.017 | 6B | 10141159 |
| lg13 | 6B_24 | 201.971 | 6B | 7582612 |
| lg13 | 6B_26 | 202.961 | 6B | 8156374 |
| lg13 | 6B_25 | 205.956 | 6B | 8032152 |
| lg13 | 6B_23 | 224.17 | 6B | 5893387 |
| lg13 | 6B_22 | 235.321 | 6B | 3196030 |
| lg13 | 6B_257 | 243.626 | IWGSC_CSS_6BS_scaff_2960555 | 784 |
| lg13 | 6B_261 | 243.629 | IWGSC_CSS_6BS_scaff_2960555 | 935 |
| lg13 | 6B_258 | 243.629 | IWGSC_CSS_6BS_scaff_2960555 | 814 |
| lg13 | 6B_262 | 243.629 | IWGSC_CSS_6BS_scaff_2960555 | 1013 |
| lg13 | 6B_259 | 243.629 | IWGSC_CSS_6BS_scaff_2960555 | 849 |
| lg13 | 6B_260 | 243.631 | IWGSC_CSS_6BS_scaff_2960555 | 918 |
| lg13 | 6B_21 | 245.889 | 6B | 1756761 |
| lg13 | 6B_266 | 248.646 | IWGSC_CSS_6BS_scaff_3003296 | 4946 |
| lg20 | 6D_30 | 0 | 6D | 15065197 |
| lg20 | 6D_124 | 2.109 | IWGSC_CSS_6DS_scaff_2100355 | 1605 |
| lg20 | 6D_3 | 4.58 | IWGSC_CSS_6DS_scaff_2125133 | 1555 |
| lg20 | 6D_37 | 5.497 | 6D | 26870239 |
| lg20 | 6D_31 | 6.176 | 6D | 18283876 |
| lg20 | 6D_38 | 7.336 | 6D | 29099863 |
| lg20 | 6D_36 | 8.314 | 6D | 26517670 |
| lg20 | 6D_5 | 8.916 | IWGSC_CSS_6DS_scaff_2083700 | 485 |
| lg20 | 6D_32 | 9.378 | 6D | 22064745 |
| lg20 | 6D_35 | 11.889 | 6D | 24874088 |
| lg20 | 6D_6 | 16.138 | IWGSC_CSS_6DS_scaff_2116527 | 6069 |
| lg20 | 6D_44 | 16.189 | 6D | 51996321 |
| lg20 | 6D_40 | 16.812 | 6D | 31670020 |
| lg20 | 6D_41 | 17.125 | 6D | 33873252 |
| lg20 | 6D_65 | 17.745 | 6D | 104659387 |
| lg20 | 6D_126 | 17.808 | IWGSC_CSS_6DS_scaff_2098765 | 107 |
| lg20 | 6D_68 | 18.059 | 6D | 113382874 |
| lg20 | 6D_66 | 18.139 | 6D | 106439672 |
| lg20 | 6D_73 | 18.312 | 6D | 135484146 |
| lg20 | 6D_45 | 18.362 | 6D | 52615191 |
| lg20 | 6D_46 | 18.431 | 6D | 53253994 |
| lg20 | 6D_56 | 18.469 | 6D | 80584575 |
| lg20 | 6D_62 | 18.583 | 6D | 98605759 |
| lg20 | 6D_58 | 18.667 | 6D | 86945961 |
| lg20 | 6D_4 | 18.893 | IWGSC_CSS_6DS_scaff_2092286 | 2106 |
| lg20 | 6D_54 | 18.919 | 6D | 75264048 |
| lg20 | 6D_59 | 19.047 | 6D | 88135301 |
| lg20 | 6D_51 | 19.194 | 6D | 64155456 |
| lg20 | 6D_52 | 19.258 | 6D | 66655763 |
| lg20 | 6D_72 | 19.47 | 6D | 129058897 |
| lg20 | 6D_57 | 19.597 | 6D | 86818610 |
| lg20 | 6D_49 | 19.881 | 6D | 54345176 |
| lg20 | 6D_75 | 20.006 | 6D | 135864056 |
| lg20 | 6D_47 | 20.259 | 6D | 54054793 |
| lg20 | 6D_48 | 20.315 | 6D | 54054804 |
| lg20 | 6D_63 | 21.11 | 6D | 99072175 |
| lg20 | 6D_123 | 23.288 | IWGSC_CSS_6DL_scaff_3328110 | 2022 |
| lg20 | 6D_50 | 23.714 | 6D | 60564764 |
| lg20 | 6D_125 | 23.894 | IWGSC_CSS_6DL_scaff_3283376 | 501 |
| lg20 | 6D_71 | 24.988 | 6D | 128641042 |
| lg20 | 6D_69 | 25.574 | 6D | 119745124 |
| lg20 | 6D_43 | 26.201 | 6D | 48992541 |
| lg20 | 6D_67 | 26.381 | 6D | 107529911 |
| lg20 | 6D_70 | 26.486 | 6D | 123591063 |
| lg20 | 6D_55 | 26.776 | 6D | 76662498 |
| lg20 | 6D_60 | 27.225 | 6D | 89450351 |
| lg20 | 6D_77 | 28.969 | 6D | 139685666 |
| lg20 | 6D_74 | 31.244 | 6D | 135699356 |
| lg20 | 6D_39 | 31.598 | 6D | 29492226 |
| lg20 | 6D_127 | 31.992 | IWGSC_CSS_6DL_scaff_3260443 | 5420 |
| lg20 | 6D_76 | 32.295 | 6D | 136337893 |
| lg20 | 6D_78 | 34.733 | 6D | 146958003 |
| lg20 | 6D_61 | 35.79 | 6D | 98102908 |
| lg20 | 6D_79 | 38.209 | 6D | 149982363 |
| lg20 | 6D_80 | 42.386 | 6D | 152069991 |
| lg20 | 6D_84 | 52.302 | 6D | 160985323 |
| lg20 | 6D_88 | 58.539 | 6D | 166091477 |
| lg20 | 6D_85 | 59.778 | 6D | 163416345 |
| lg20 | 6D_83 | 60.388 | 6D | 160367276 |
| lg20 | 6D_122 | 60.984 | IWGSC_CSS_6DL_scaff_3299485 | 4443 |
| lg20 | 6D_87 | 66.352 | 6D | 165443403 |
| lg20 | 6D_86 | 68.241 | 6D | 164510693 |
| lg20 | 6D_89 | 72.442 | 6D | 167159401 |
| lg20 | 6D_42 | 87.909 | 6D | 42398255 |
| lg20 | 6D_92 | 88.434 | 6D | 169996096 |
| lg20 | 6D_91 | 88.483 | 6D | 169996000 |
| lg20 | 6D_93 | 88.487 | 6D | 169996201 |
| lg20 | 6D_90 | 88.487 | 6D | 169995981 |
| lg20 | 6D_98 | 89.078 | 6D | 171441730 |
| lg20 | 6D_97 | 89.078 | 6D | 171441712 |
| lg20 | 6D_96 | 89.078 | 6D | 171441683 |
| lg20 | 6D_99 | 89.175 | 6D | 171441746 |
| lg20 | 6D_94 | 90.067 | 6D | 170826008 |
| lg20 | 6D_95 | 90.998 | 6D | 171216260 |
| lg20 | 6D_120 | 107.58 | 6D | 175425276 |
| lg20 | 6D_110 | 111.823 | 6D | 174201470 |
| lg20 | 6D_109 | 111.857 | 6D | 174201358 |
| lg20 | 6D_111 | 112.107 | 6D | 174300286 |
| lg20 | 6D_113 | 112.545 | 6D | 174361774 |
| lg20 | 6D_112 | 112.589 | 6D | 174361611 |
| lg20 | 6D_119 | 113.92 | 6D | 175391727 |
| lg20 | 6D_101 | 114.576 | 6D | 172244418 |
| lg20 | 6D_102 | 114.643 | 6D | 172408083 |
| lg20 | 6D_103 | 114.643 | 6D | 172408116 |
| lg20 | 6D_105 | 114.951 | 6D | 173181742 |
| lg20 | 6D_104 | 114.954 | 6D | 173181722 |
| lg20 | 6D_108 | 114.954 | 6D | 173181895 |
| lg20 | 6D_107 | 115.052 | 6D | 173181811 |
| lg20 | 6D_106 | 115.069 | 6D | 173181792 |
| lg20 | 6D_115 | 115.753 | 6D | 174464426 |
| lg20 | 6D_116 | 115.831 | 6D | 174464440 |
| lg20 | 6D_100 | 116.017 | 6D | 171999228 |
| lg20 | 6D_114 | 116.233 | 6D | 174464093 |
| lg20 | 6D_1 | 116.963 | IWGSC_CSS_6DL_scaff_3262345 | 2605 |
| lg20 | 6D_117 | 117.493 | 6D | 174878249 |
| lg20 | 6D_118 | 117.493 | 6D | 174878409 |
| lg07 | 7A_171 | 0 | 7A | 181492788 |
| lg07 | 7A_172 | 0.007 | 7A | 181492845 |
| lg07 | 7A_166 | 1.328 | 7A | 179817824 |
| lg07 | 7A_17 | 3.181 | IWGSC_CSS_7AL_scaff_4557336 | 2129 |
| lg07 | 7A_165 | 15.704 | 7A | 178361084 |
| lg07 | 7A_163 | 18.608 | 7A | 177341295 |
| lg07 | 7A_176 | 21.262 | IWGSC_CSS_7AL_scaff_4443517 | 3095 |
| lg07 | 7A_177 | 21.294 | IWGSC_CSS_7AL_scaff_4443517 | 3160 |
| lg07 | 7A_178 | 21.34 | IWGSC_CSS_7AL_scaff_4443517 | 3208 |
| lg07 | 7A_164 | 22.364 | 7A | 177601488 |
| lg07 | 7A_162 | 25.482 | 7A | 175697699 |
| lg07 | 7A_161 | 30.851 | 7A | 174645189 |
| lg07 | 7A_159 | 35.941 | 7A | 173355409 |
| lg07 | 7A_158 | 35.941 | 7A | 173355397 |
| lg07 | 7A_156 | 36.433 | 7A | 172106307 |
| lg07 | 7A_157 | 36.433 | 7A | 172106479 |
| lg07 | 7A_160 | 36.904 | 7A | 173467925 |
| lg07 | 7A_154 | 50.016 | 7A | 167254077 |
| lg07 | 7A_155 | 50.016 | 7A | 167254108 |
| lg07 | 7A_152 | 56.691 | 7A | 165182468 |
| lg07 | 7A_151 | 57.285 | 7A | 165054095 |
| lg07 | 7A_150 | 57.547 | 7A | 165053960 |
| lg07 | 7A_153 | 58.531 | 7A | 166291818 |
| lg07 | 7A_13 | 62.83 | IWGSC_CSS_7AL_scaff_4556551 | 4802 |
| lg07 | 7A_12 | 63.172 | IWGSC_CSS_7AL_scaff_4458184 | 932 |
| lg07 | 7A_208 | 63.877 | IWGSC_CSS_7AL_scaff_4538509 | 5097 |
| lg07 | 7A_207 | 63.899 | IWGSC_CSS_7AL_scaff_4538509 | 5094 |
| lg07 | 7A_149 | 66.137 | 7A | 163432106 |
| lg07 | 7A_148 | 68.494 | 7A | 161922281 |
| lg07 | 7A_147 | 77.14 | 7A | 159621863 |
| lg07 | 7A_146 | 87.511 | 7A | 157455545 |
| lg07 | 7A_197 | 88.164 | IWGSC_CSS_7AL_scaff_4554167 | 1369 |
| lg07 | 7A_196 | 88.164 | IWGSC_CSS_7AL_scaff_4554167 | 1348 |
| lg07 | 7A_198 | 88.164 | IWGSC_CSS_7AL_scaff_4554167 | 1555 |
| lg07 | 7A_145 | 90.426 | 7A | 157000164 |
| lg07 | 7A_8 | 91.831 | IWGSC_CSS_7AL_scaff_4546052 | 841 |
| lg07 | 7A_9 | 91.975 | IWGSC_CSS_7AL_scaff_4546052 | 908 |
| lg07 | 7A_7 | 91.975 | IWGSC_CSS_7AL_scaff_4546052 | 790 |
| lg07 | 7A_6 | 92.029 | IWGSC_CSS_7AL_scaff_4546052 | 761 |
| lg07 | 7A_199 | 92.302 | IWGSC_CSS_7AL_scaff_4432508 | 3289 |
| lg07 | 7A_140 | 94.477 | 7A | 149565631 |
| lg07 | 7A_143 | 95.183 | 7A | 153116011 |
| lg07 | 7A_141 | 95.327 | 7A | 153115876 |
| lg07 | 7A_142 | 95.332 | 7A | 153116006 |
| lg07 | 7A_144 | 98.271 | 7A | 155422492 |
| lg07 | 7A_132 | 104.515 | 7A | 141241826 |
| lg07 | 7A_131 | 104.678 | 7A | 141241807 |
| lg07 | 7A_133 | 104.807 | 7A | 141309295 |
| lg07 | 7A_127 | 104.896 | 7A | 133248208 |
| lg07 | 7A_126 | 104.903 | 7A | 133248142 |
| lg07 | 7A_129 | 105.055 | 7A | 138511578 |
| lg07 | 7A_123 | 106.178 | 7A | 127618449 |
| lg07 | 7A_11 | 106.614 | IWGSC_CSS_7AL_scaff_4430146 | 3323 |
| lg07 | 7A_10 | 106.625 | IWGSC_CSS_7AL_scaff_4430146 | 3321 |
| lg07 | 7A_125 | 106.815 | 7A | 130184407 |
| lg07 | 7A_117 | 107.089 | 7A | 126761355 |
| lg07 | 7A_120 | 107.128 | 7A | 126761522 |
| lg07 | 7A_119 | 107.135 | 7A | 126761519 |
| lg07 | 7A_121 | 107.143 | 7A | 126761563 |
| lg07 | 7A_118 | 107.146 | 7A | 126761441 |
| lg07 | 7A_122 | 107.39 | 7A | 127298960 |
| lg07 | 7A_116 | 107.681 | 7A | 124440892 |
| lg07 | 7A_128 | 107.94 | 7A | 138397387 |
| lg07 | 7A_53 | 116.64 | 7A | 45514361 |
| lg07 | 7A_14 | 117.991 | IWGSC_CSS_7AS_scaff_4256923 | 460 |
| lg07 | 7A_191 | 119.421 | IWGSC_CSS_7AL_scaff_4486452 | 1071 |
| lg07 | 7A_4 | 119.925 | IWGSC_CSS_7AL_scaff_4490433 | 13297 |
| lg07 | 7A_3 | 120.006 | IWGSC_CSS_7AL_scaff_4490433 | 13226 |
| lg07 | 7A_201 | 120.502 | IWGSC_CSS_7AS_scaff_4207649 | 4378 |
| lg07 | 7A_200 | 120.633 | IWGSC_CSS_7AL_scaff_4399057 | 3657 |
| lg07 | 7A_202 | 120.854 | IWGSC_CSS_7AS_scaff_4207649 | 4497 |
| lg07 | 7A_86 | 121.233 | 7A | 74486283 |
| lg07 | 7A_174 | 121.354 | IWGSC_CSS_7AS_scaff_4117484 | 1431 |
| lg07 | 7A_114 | 121.518 | 7A | 110169283 |
| lg07 | 7A_54 | 122.357 | 7A | 45531046 |
| lg07 | 7A_95 | 122.613 | 7A | 92588269 |
| lg07 | 7A_77 | 122.672 | 7A | 59539025 |
| lg07 | 7A_78 | 122.696 | 7A | 59539062 |
| lg07 | 7A_70 | 123.15 | 7A | 55943479 |
| lg07 | 7A_106 | 123.151 | 7A | 104553160 |
| lg07 | 7A_99 | 123.157 | 7A | 102959851 |
| lg07 | 7A_109 | 123.329 | 7A | 109107533 |
| lg07 | 7A_104 | 123.329 | 7A | 103754139 |
| lg07 | 7A_101 | 123.333 | 7A | 103753959 |
| lg07 | 7A_102 | 123.333 | 7A | 103753987 |
| lg07 | 7A_105 | 123.333 | 7A | 103754142 |
| lg07 | 7A_100 | 123.333 | 7A | 103753949 |
| lg07 | 7A_103 | 123.352 | 7A | 103754122 |
| lg07 | 7A_108 | 123.606 | 7A | 108723385 |
| lg07 | 7A_41 | 123.755 | 7A | 34373222 |
| lg07 | 7A_111 | 123.793 | 7A | 109571456 |
| lg07 | 7A_112 | 123.803 | 7A | 109571534 |
| lg07 | 7A_42 | 123.916 | 7A | 34373228 |
| lg07 | 7A_107 | 124.041 | 7A | 104553191 |
| lg07 | 7A_96 | 124.248 | 7A | 94073324 |
| lg07 | 7A_98 | 124.348 | 7A | 101241352 |
| lg07 | 7A_110 | 124.64 | 7A | 109338958 |
| lg07 | 7A_173 | 124.957 | IWGSC_CSS_7AS_scaff_4217727 | 7676 |
| lg07 | 7A_83 | 125.314 | 7A | 67080974 |
| lg07 | 7A_82 | 125.626 | 7A | 66959984 |
| lg07 | 7A_81 | 125.652 | 7A | 66959972 |
| lg07 | 7A_51 | 126.113 | 7A | 43055785 |
| lg07 | 7A_80 | 126.904 | 7A | 60320836 |
| lg07 | 7A_124 | 127.21 | 7A | 129591736 |
| lg07 | 7A_37 | 138.558 | 7A | 27554440 |
| lg07 | 7A_36 | 140.562 | 7A | 26564736 |
| lg07 | 7A_75 | 146.649 | 7A | 56102044 |
| lg07 | 7A_35 | 147.359 | 7A | 20689453 |
| lg07 | 7A_34 | 147.367 | 7A | 20689434 |
| lg07 | 7A_184 | 155.878 | IWGSC_CSS_7AS_scaff_4252814 | 4998 |
| lg07 | 7A_183 | 155.878 | IWGSC_CSS_7AS_scaff_4252814 | 4995 |
| lg07 | 7A_181 | 155.944 | IWGSC_CSS_7AS_scaff_4252814 | 4949 |
| lg07 | 7A_180 | 155.944 | IWGSC_CSS_7AS_scaff_4252814 | 4894 |
| lg07 | 7A_186 | 155.945 | IWGSC_CSS_7AS_scaff_4252814 | 5046 |
| lg07 | 7A_182 | 155.947 | IWGSC_CSS_7AS_scaff_4252814 | 4973 |
| lg07 | 7A_185 | 155.956 | IWGSC_CSS_7AS_scaff_4252814 | 5008 |
| lg07 | 7A_169 | 157.994 | 7A | 180078390 |
| lg07 | 7A_168 | 158.045 | 7A | 180078214 |
| lg07 | 7A_167 | 158.046 | 7A | 180078194 |
| lg07 | 7A_33 | 164.455 | 7A | 19288006 |
| lg07 | 7A_32 | 164.481 | 7A | 19287990 |
| lg07 | 7A_31 | 164.83 | 7A | 19174745 |
| lg07 | 7A_30 | 164.841 | 7A | 19174739 |
| lg07 | 7A_29 | 164.874 | 7A | 19174738 |
| lg07 | 7A_187 | 179.974 | IWGSC_CSS_7AS_scaff_4249835 | 3715 |
| lg07 | 7A_189 | 179.984 | IWGSC_CSS_7AS_scaff_4249835 | 3742 |
| lg07 | 7A_188 | 179.993 | IWGSC_CSS_7AS_scaff_4249835 | 3741 |
| lg07 | 7A_22 | 182.685 | 7A | 12801233 |
| lg07 | 7A_21 | 183.86 | 7A | 12153721 |
| lg14 | 7B_134 | 0 | 7B | 251510527 |
| lg14 | 7B_131 | 2.683 | 7B | 248698212 |
| lg14 | 7B_133 | 3.168 | 7B | 249989434 |
| lg14 | 7B_132 | 7.635 | 7B | 248960011 |
| lg14 | 7B_129 | 10.734 | 7B | 244898182 |
| lg14 | 7B_130 | 12.256 | 7B | 245787712 |
| lg14 | 7B_128 | 21.796 | 7B | 243914338 |
| lg14 | 7B_126 | 33.162 | 7B | 242260073 |
| lg14 | 7B_127 | 33.17 | 7B | 242260164 |
| lg14 | 7B_123 | 33.173 | 7B | 242260052 |
| lg14 | 7B_124 | 33.185 | 7B | 242260068 |
| lg14 | 7B_125 | 33.186 | 7B | 242260071 |
| lg14 | 7B_3 | 40.176 | IWGSC_CSS_7BL_scaff_6701524 | 3022 |
| lg14 | 7B_119 | 56.013 | 7B | 236323185 |
| lg14 | 7B_122 | 56.975 | 7B | 236754144 |
| lg14 | 7B_120 | 56.975 | 7B | 236754120 |
| lg14 | 7B_121 | 56.994 | 7B | 236754138 |
| lg14 | 7B_118 | 57.658 | 7B | 235782164 |
| lg14 | 7B_116 | 67.672 | 7B | 232607882 |
| lg14 | 7B_6 | 67.984 | IWGSC_CSS_7BL_scaff_1617014 | 100 |
| lg14 | 7B_7 | 67.987 | IWGSC_CSS_7BL_scaff_1617014 | 188 |
| lg14 | 7B_5 | 68.051 | IWGSC_CSS_7BL_scaff_1617014 | 82 |
| lg14 | 7B_2 | 68.659 | IWGSC_CSS_7BL_scaff_6740180 | 2286 |
| lg14 | 7B_1 | 68.68 | IWGSC_CSS_7BL_scaff_6740180 | 2283 |
| lg14 | 7B_115 | 68.711 | 7B | 230043933 |
| lg14 | 7B_114 | 68.746 | 7B | 230043774 |
| lg14 | 7B_117 | 68.886 | 7B | 233245797 |
| lg14 | 7B_110 | 83.91 | 7B | 229279757 |
| lg14 | 7B_112 | 83.91 | 7B | 229279863 |
| lg14 | 7B_111 | 84.09 | 7B | 229279790 |
| lg14 | 7B_113 | 84.212 | 7B | 229279887 |
| lg14 | 7B_109 | 84.221 | 7B | 229279755 |
| lg14 | 7B_108 | 87.954 | 7B | 228673655 |
| lg14 | 7B_105 | 88.596 | 7B | 226014825 |
| lg14 | 7B_107 | 88.678 | 7B | 226014973 |
| lg14 | 7B_106 | 88.811 | 7B | 226014952 |
| lg14 | 7B_104 | 89.036 | 7B | 226014739 |
| lg14 | 7B_102 | 89.372 | 7B | 222916394 |
| lg14 | 7B_103 | 89.372 | 7B | 222916418 |
| lg14 | 7B_96 | 89.526 | 7B | 214933321 |
| lg14 | 7B_100 | 89.564 | 7B | 220791099 |
| lg14 | 7B_101 | 89.574 | 7B | 220791190 |
| lg14 | 7B_97 | 89.587 | 7B | 216077438 |
| lg14 | 7B_98 | 89.855 | 7B | 217700175 |
| lg14 | 7B_99 | 89.892 | 7B | 217700179 |
| lg14 | 7B_91 | 96.736 | 7B | 207673649 |
| lg14 | 7B_90 | 96.816 | 7B | 207673611 |
| lg14 | 7B_85 | 97.497 | 7B | 202786172 |
| lg14 | 7B_82 | 97.668 | 7B | 186938244 |
| lg14 | 7B_94 | 97.817 | 7B | 211822529 |
| lg14 | 7B_89 | 98.006 | 7B | 207353905 |
| lg14 | 7B_83 | 98.28 | 7B | 202317123 |
| lg14 | 7B_84 | 98.291 | 7B | 202317150 |
| lg14 | 7B_92 | 98.498 | 7B | 209324514 |
| lg14 | 7B_93 | 98.648 | 7B | 209386338 |
| lg14 | 7B_95 | 98.769 | 7B | 213382580 |
| lg14 | 7B_86 | 99.023 | 7B | 203193734 |
| lg14 | 7B_88 | 99.544 | 7B | 204761938 |
| lg14 | 7B_87 | 99.726 | 7B | 204761893 |
| lg14 | 7B_81 | 102.857 | 7B | 175760951 |
| lg14 | 7B_136 | 106.119 | IWGSC_CSS_7BL_scaff_6707770 | 2068 |
| lg14 | 7B_135 | 106.788 | IWGSC_CSS_7BL_scaff_6695910 | 3854 |
| lg14 | 7B_137 | 107.073 | IWGSC_CSS_7BL_scaff_6734669 | 234 |
| lg14 | 7B_62 | 110.154 | 7B | 87491201 |
| lg14 | 7B_75 | 111.001 | 7B | 137179010 |
| lg14 | 7B_60 | 111.292 | 7B | 84886576 |
| lg14 | 7B_55 | 111.408 | 7B | 82504630 |
| lg14 | 7B_73 | 111.809 | 7B | 135141334 |
| lg14 | 7B_72 | 111.849 | 7B | 135001023 |
| lg14 | 7B_64 | 112.197 | 7B | 94401953 |
| lg14 | 7B_63 | 112.299 | 7B | 87553344 |
| lg14 | 7B_44 | 112.538 | 7B | 63246495 |
| lg14 | 7B_45 | 112.98 | 7B | 63640487 |
| lg14 | 7B_54 | 113.14 | 7B | 82295834 |
| lg14 | 7B_42 | 113.324 | 7B | 60007552 |
| lg14 | 7B_49 | 113.395 | 7B | 72039867 |
| lg14 | 7B_51 | 113.436 | 7B | 72040071 |
| lg14 | 7B_50 | 113.443 | 7B | 72039886 |
| lg14 | 7B_37 | 113.514 | 7B | 42475292 |
| lg14 | 7B_39 | 113.562 | 7B | 54139811 |
| lg14 | 7B_40 | 113.617 | 7B | 54140016 |
| lg14 | 7B_53 | 113.651 | 7B | 75986112 |
| lg14 | 7B_47 | 113.687 | 7B | 70124566 |
| lg14 | 7B_71 | 113.752 | 7B | 132134503 |
| lg14 | 7B_70 | 113.846 | 7B | 105521356 |
| lg14 | 7B_69 | 113.851 | 7B | 105521351 |
| lg14 | 7B_35 | 113.872 | 7B | 41508732 |
| lg14 | 7B_36 | 113.913 | 7B | 41508825 |
| lg14 | 7B_34 | 113.913 | 7B | 41508676 |
| lg14 | 7B_59 | 113.981 | 7B | 83291168 |
| lg14 | 7B_67 | 114.032 | 7B | 102699528 |
| lg14 | 7B_78 | 114.042 | 7B | 151080735 |
| lg14 | 7B_66 | 114.051 | 7B | 102699513 |
| lg14 | 7B_79 | 114.084 | 7B | 151080850 |
| lg14 | 7B_43 | 114.117 | 7B | 62806910 |
| lg14 | 7B_80 | 114.183 | 7B | 164564344 |
| lg14 | 7B_52 | 114.249 | 7B | 75908175 |
| lg14 | 7B_65 | 114.318 | 7B | 102613295 |
| lg14 | 7B_38 | 114.38 | 7B | 44970391 |
| lg14 | 7B_48 | 114.506 | 7B | 72035366 |
| lg14 | 7B_77 | 114.695 | 7B | 144754776 |
| lg14 | 7B_68 | 114.828 | 7B | 104473619 |
| lg14 | 7B_76 | 115.208 | 7B | 138387193 |
| lg14 | 7B_33 | 115.59 | 7B | 40436137 |
| lg14 | 7B_74 | 116.961 | 7B | 137178999 |
| lg14 | 7B_4 | 117.467 | IWGSC_CSS_7BS_scaff_3105531 | 2163 |
| lg14 | 7B_61 | 118.684 | 7B | 87491188 |
| lg14 | 7B_58 | 119.37 | 7B | 82504786 |
| lg14 | 7B_57 | 119.387 | 7B | 82504723 |
| lg14 | 7B_56 | 119.403 | 7B | 82504632 |
| lg14 | 7B_46 | 119.736 | 7B | 69771231 |
| lg14 | 7B_32 | 119.986 | 7B | 32222177 |
| lg14 | 7B_31 | 120.504 | 7B | 31700456 |
| lg14 | 7B_30 | 127.031 | 7B | 26362006 |
| lg14 | 7B_29 | 127.213 | 7B | 26361985 |
| lg14 | 7B_13 | 128.594 | 7B | 8160841 |
| lg14 | 7B_14 | 128.617 | 7B | 8160886 |
| lg14 | 7B_12 | 129.16 | 7B | 7204082 |
| lg14 | 7B_28 | 129.616 | 7B | 19907220 |
| lg14 | 7B_9 | 133.266 | IWGSC_CSS_7BS_scaff_3088687 | 2231 |
| lg14 | 7B_24 | 133.786 | 7B | 18863353 |
| lg14 | 7B_25 | 133.808 | 7B | 18863422 |
| lg14 | 7B_23 | 133.829 | 7B | 18863292 |
| lg14 | 7B_16 | 134.272 | 7B | 11818736 |
| lg14 | 7B_17 | 134.273 | 7B | 11818742 |
| lg14 | 7B_18 | 134.274 | 7B | 11818828 |
| lg14 | 7B_19 | 134.274 | 7B | 11818888 |
| lg14 | 7B_15 | 134.281 | 7B | 11818725 |
| lg14 | 7B_20 | 134.325 | 7B | 13183833 |
| lg14 | 7B_22 | 134.598 | 7B | 15222334 |
| lg14 | 7B_21 | 134.642 | 7B | 14849890 |
| lg14 | 7B_8 | 134.815 | IWGSC_CSS_7BS_scaff_3162401 | 690 |
| lg14 | 7B_27 | 135.142 | 7B | 19799297 |
| lg14 | 7B_26 | 135.161 | 7B | 19799293 |
| lg21 | 7D_32 | 0 | 7D | 198134128 |
| lg21 | 7D_34 | 0.236 | 7D | 203127388 |
| lg21 | 7D_36 | 0.241 | 7D | 203127442 |
| lg21 | 7D_35 | 0.262 | 7D | 203127417 |
| lg21 | 7D_38 | 0.282 | 7D | 203127550 |
| lg21 | 7D_37 | 0.301 | 7D | 203127472 |
| lg21 | 7D_33 | 3.277 | 7D | 202338004 |
| lg21 | 7D_31 | 3.671 | 7D | 185860456 |
| lg21 | 7D_30 | 4.958 | 7D | 185368714 |
| lg21 | 7D_12 | 8.711 | 7D | 47339220 |
| lg21 | 7D_25 | 9.008 | 7D | 161876739 |
| lg21 | 7D_14 | 9.484 | 7D | 57742352 |
| lg21 | 7D_13 | 11.93 | 7D | 50298470 |
| lg21 | 7D_19 | 12.078 | 7D | 85471011 |
| lg21 | 7D_26 | 12.986 | 7D | 163005202 |
| lg21 | 7D_15 | 13.73 | 7D | 70038627 |
| lg21 | 7D_24 | 15.384 | 7D | 157977381 |
| lg21 | 7D_43 | 18.214 | IWGSC_CSS_7DL_scaff_3392388 | 3885 |
| lg21 | 7D_17 | 19.638 | 7D | 84732657 |
| lg21 | 7D_27 | 20.125 | 7D | 165478534 |
| lg21 | 7D_21 | 20.345 | 7D | 126330842 |
| lg21 | 7D_18 | 20.78 | 7D | 85289856 |
| lg21 | 7D_28 | 21.703 | 7D | 167576002 |
| lg21 | 7D_22 | 22.867 | 7D | 127337095 |
| lg21 | 7D_23 | 32.514 | 7D | 141837722 |
| lg21 | 7D_8 | 35.186 | 7D | 31698026 |
| lg21 | 7D_1 | 35.557 | IWGSC_CSS_7DS_scaff_3893906 | 4632 |
| lg21 | 7D_11 | 36.209 | 7D | 38902127 |
| lg21 | 7D_10 | 37.95 | 7D | 32611427 |
| lg21 | 7D_42 | 41.335 | IWGSC_CSS_7DS_scaff_3913232 | 1424 |
| lg21 | 7D_9 | 42.909 | 7D | 31710813 |
| lg21 | 7D_44 | 51.725 | IWGSC_CSS_7DS_scaff_3903885 | 1812 |
| lg21 | 7D_41 | 57.728 | IWGSC_CSS_7DS_scaff_3856153 | 1608 |
| lg21 | 7D_4 | 71.178 | 7D | 17973127 |
| lg21 | 7D_3 | 71.182 | 7D | 17973096 |
| lg21 | 7D_6 | 79.839 | 7D | 24985037 |
| lg21 | 7D_7 | 79.894 | 7D | 24985134 |
| lg21 | 7D_5 | 80.546 | 7D | 24041958 |

**Table S2.**The phenotypic of the F_2_ individuals in HTS-1 × CM28TP

| No. of the F_2_ individual | phenotypic | No. of the F_2_ individual | phenotypic | No. of the F_2_ individual | phenotypic | No. of the F_2_ individual | phenotypic |
| --- | --- | --- | --- | --- | --- | --- | --- |
| CM28TP | normal | 67 | normal | 137 | normal | 210 | normal |
| HTS-1 | pistillody | 68 | normal | 138 | normal | 212 | normal |
| 2 | normal | 69 | normal | 139 | normal | 213 | normal |
| 3 | normal | 70 | normal | 140 | normal | 219 | pistillody |
| 6 | pistillody | 71 | normal | 141 | normal | 257 | normal |
| 7 | normal | 72 | normal | 142 | normal | 261 | normal |
| 8 | normal | 73 | normal | 144 | normal | 277 | pistillody |
| 11 | normal | 74 | normal | 145 | normal | 281 | pistillody |
| 13 | normal | 76 | normal | 146 | normal | 298 | pistillody |
| 14 | normal | 77 | normal | 147 | pistillody | 332 | normal |
| 15 | normal | 78 | normal | 148 | normal | 334 | pistillody |
| 16 | normal | 79 | normal | 149 | normal | 341 | normal |
| 17 | normal | 80 | normal | 152 | pistillody | 342 | normal |
| 18 | normal | 81 | normal | 153 | normal | 356 | normal |
| 19 | normal | 82 | pistillody | 154 | pistillody | 388 | pistillody |
| 20 | normal | 83 | normal | 155 | normal | 394 | normal |
| 21 | normal | 84 | normal | 156 | normal | 399 | pistillody |
| 22 | pistillody | 85 | normal | 160 | normal | 418 | normal |
| 23 | normal | 86 | normal | 161 | normal | 419 | pistillody |
| 25 | normal | 87 | pistillody | 163 | normal | 438 | pistillody |
| 27 | normal | 88 | normal | 164 | normal | 439 | pistillody |
| 28 | normal | 89 | normal | 165 | normal | 440 | normal |
| 29 | normal | 90 | pistillody | 166 | normal | 473 | normal |
| 30 | normal | 91 | normal | 167 | pistillody | 475 | normal |
| 32 | normal | 95 | normal | 168 | normal | 479 | pistillody |
| 33 | normal | 97 | pistillody | 169 | pistillody | 480 | normal |
| 34 | normal | 99 | normal | 170 | normal | 497 | normal |
| 35 | normal | 101 | normal | 172 | normal | 502 | normal |
| 36 | normal | 104 | normal | 174 | normal | 509 | pistillody |
| 38 | normal | 108 | normal | 175 | pistillody | 533 | normal |
| 39 | normal | 109 | normal | 176 | normal | 567 | pistillody |
| 40 | normal | 110 | normal | 178 | normal | 628 | normal |
| 41 | normal | 112 | normal | 181 | normal | 660 | normal |
| 43 | normal | 113 | normal | 184 | normal | 687 | pistillody |
| 44 | normal | 115 | normal | 186 | normal | 697 | normal |
| 45 | normal | 116 | normal | 188 | normal | 718 | pistillody |
| 46 | normal | 118 | pistillody | 189 | normal | 719 | pistillody |
| 47 | normal | 120 | normal | 190 | normal | 754 | normal |
| 48 | normal | 121 | normal | 191 | normal | 813 | normal |
| 49 | normal | 122 | normal | 192 | pistillody | 921 | pistillody |
| 50 | normal | 124 | normal | 193 | normal | 922 | pistillody |
| 54 | normal | 125 | normal | 194 | pistillody | 1132 | pistillody |
| 56 | normal | 126 | pistillody | 195 | normal | 1147 | normal |
| 57 | normal | 127 | normal | 198 | normal | 1149 | normal |
| 59 | normal | 128 | normal | 199 | normal | 1150 | normal |
| 60 | normal | 129 | pistillody | 200 | pistillody | 1167 | pistillody |
| 61 | pistillody | 132 | normal | 203 | normal | 1168 | normal |
| 62 | normal | 133 | normal | 204 | pistillody | 1182 | normal |
| 63 | normal | 134 | pistillody | 205 | normal | 1187 | pistillody |
| 64 | normal | 135 | pistillody | 208 | pistillody |  |  |
| 65 | normal | 136 | pistillody | 209 | normal |  |  |

**Table S3.** Genes located in the intervals of *hts*

| Gene ID | BLAST matching accessionNo. | Annotation | identities |
| --- | --- | --- | --- |
| Traes_4AL_00DB56D41.4 | XM 020294050.1 | BEL1-like homeodomain protein 7 (*Aegilops tauschii* ) | 99% |
| Traes_4AL_0A4CEFC07.2 | XM 020325547.1 | ankyrin repeat domain-containing protein 65-like (Aegilops tauschii ) | 98% |
| Traes_4AL_0BE08D907.1 | BT009232.1 | N/A | 99% |
| Traes_4AL_0C2B420B0.2 | KR055812.1 | early flowering 3-D1 (ELF3-D1) gene （*Triticum aestivum*） | 89% |
| Traes_4AL_0D6C3F8F2.2 | XM 0202323343.1 | suppressor of disruption of TFIIS-like (*Aegilops tauschii* ) | 94% |
| Traes_4AL_0F80D14341.1 | XM 020339205.1 | ethanolamine-phosphate cytidylyltransferase-like (*Aegilops tauschii* ) | 99% |
| Traes_4AL_0FE4AF9D9.1 | XM 020318156.1 | 2-methyl-6-phytyl-1,4-hydroquinone methyltransferase 2 (*Aegilops tauschii* ) | 99% |
| Traes_4AL_1AB7E5F8C.2 | JQ922097.1 | isolate H7 4-hydroxy-3-methylbut-2-enyl diphosphate reductase (Hdr) gene | 91% |
| Traes_4AL_1AF04D69F.1 | KR816810.1 | VRN-B1 （*Triticum aestivum*） | 98% |
| Traes_4AL_1B8226A1D.1 | HG670306.1 | oxysterol-binding protein-related protein 1C （*Triticum aestivum*） | 100% |
| Traes_4AL_1BABE7D89.2 | XM 020300878.1 | beta-glucuronosyltransferase GlcAT14B-like (*Aegilops tauschii* ) | 95% |
| Traes_4AL_1C649DD2E.1 | XM 020325555.1 | PHR1-LIKE 3-like (*Aegilops tauschii* ) | 91% |
| Traes_4AL_1CC83F0F7.1 | XM 020327774.1 | N/A | 98% |
| Traes_4AL_1CD626203.1 | KF242512.1 | cytosolic glutamine synthetase (GSe-A4) gene (*Triticum turgidum*) | 99% |
| Traes_4AL_1CDBA1964.1 | XM 004974284.1 | RNA-dependent RNA polymerase SHL2 (*Setaria italica*) | 83% |
| Traes_4AL_1D4A5919A1.4 | XM 020340351.1 | NRT1/ PTR FAMILY 2.11-like (*Aegilops tauschii*) | 98% |
| Traes_4AL_1D564CFC3.1 | XM 020303830.1 | COBRA-like protein 2 (*Aegilops tauschii*) | 98% |
| Traes_4AL_1E9C37327.1 | XM 0203339330.1 | peptidyl-prolyl cis-trans isomerase FKBP17-2 (*Aegilops tauschii*) | 97% |
| Traes_4AL_02AE47773.1 | XM 020302836.1 | N/A | 83% |
| Traes_4AL_02F7D1B1F.1 | XM 020300956.1 | monogalactosyldiacylglycerol synthase 2 (*Aegilops tauschii*) | 97% |
| Traes_4AL_2A2E81E05.5 | AK250326.1 | N/A | 97% |
| Traes_4AL_2ADD2BE44.1 | XM 020324223.1 | protein argonaute-2-like ( *Aegilops tauschii*) | 99% |
| Traes_4AL_2B70B04B6.1 | XM 020302187.1 | proteinase Asp1-like ( *Aegilops tauschii*) | 99% |
| Traes_4AL_2BCB74D01.1 | XR 002237107.1 | DEAD-box ATP-dependent RNA helicase 21 ( *Aegilops tauschii*) | 98% |
| Traes_4AL_2CA8F34F5.1 | XM 020334326.1 | transcription factor RL9 ( *Aegilops tauschii*) | 99% |
| Traes_4AL_2D42DAE24.1 | XM 020316547.1 | N/A | 98% |
| Traes_4AL_2D1616A52.2 | XM 020314134.1 | protein ETHYLENE-INSENSITIVE 2-like ( *Aegilops tauschii*) | 93% |
| Traes_4AL_2E9B496F6.1 | XM 020300997.1 | protein CASP (*Aegilops tauschii*) | 99% |
| Traes_4AL_2E796609C.1 | XM 020329724.1 | metal transporter Nramp6-like (*Aegilops tauschii*) | 97% |
| Traes_4AL_2EBA96358.2 | XM 020333825.1 | protein FAR1-RELATED SEQUENCE 5-like (*Aegilops tauschii*) | 74% |
| Traes_4AL_2FC39B934.1 | XM 020292676.1 | disease resistance protein RXW24L (*Aegilops tauschii*) | 85% |
| Traes_4AL_2FC8027D2.2 | XR 002232884.1 | N/A | 84% |
| Traes_4AL_3A4B86F31.1 | XM 020292669.1 | disease resistance RPP13-like protein 2 (*Aegilops tauschii*) | 86% |
| Traes_4AL_3C1D2E181.2 | XM 020297357.1 | F-box/LRR-repeat protein 12 (*Aegilops tauschii*) | 97% |
| Traes_4AL_3D2FDD95F.1 | XM 020292680.1 | cysteine-rich receptor-like protein kinase 3 (*Aegilops tauschii*) | 99% |
| Traes_4AL_3D6935A98.2 | XM 020314296.1 | heparan-alpha-glucosaminide N-acetyltransferase-like (*Aegilops tauschii*) | 97% |
| Traes_4AL_3F0864D1C.2 | XM 020328392.1 | serine/threonine-protein kinase CTR1-like (*Aegilops tauschii*) | 99% |
| Traes_4AL_3FB46969C.1 | XM 020294041.1 | protein SABRE (*Aegilops tauschii*) | 98% |
| Traes_4AL_3FFAD44ED.2 | XM 020332058.1 | N/A | 96% |
| Traes_4AL_04BA185C1.1 | XM_020322663.1 | serpin-Z2B (*Aegilops tauschii*) | 80% |
| Traes_4AL_04F2491C2.1 | XM 020312895.1 | cortical cell-delineating protein-like (*Aegilops tauschii*) | 93% |
| Traes_4AL_4A4D6D929.1 | XM 020345482.1 | N/A | 94% |
| Traes_4AL_4AE9E13B4.2 | XM 020321041.1 | beta-D-xylosidase 7 (*Aegilops tauschii*) | 96% |
| Traes_4AL_4AE54FC65.1 | XM 020345442.1 | LRR receptor-like serine/threonine-protein kinase At2g16250 (*Aegilops tauschii*) | 98% |
| Traes_4AL_4CE51A4CA.1 | XM 020327062.1 | mRNA-decapping enzyme-like protein (*Aegilops tauschii*) | 97% |
| Traes_4AL_4D0EE1369.1 | XM 020304695.1 | N/A | 99% |
| Traes_4AL_4D9D7CCD2.1 | XM 020297990.1 | acetolactate synthase small subunit 2, chloroplastic-like (*Aegilops tauschii*) | 98% |
| Traes_4AL_4E6C408DC.1 | XM 020312856.1 | putative 3,4-dihydroxy-2-butanone kinase (*Aegilops tauschii*) | 99% |
| Traes_4AL_4E9796DCB.1 | XM 020293741.1 | N/A | 96% |
| Traes_4AL_4F8DD86CD.1 | XM 020310777.1 | tRNA (cytosine(34)-C(5))-methyltransferase-like (*Aegilops tauschii*) | 95% |
| Traes_4AL_4FF85D8E9.1 | XM 020301693.1 | N/A | 95% |
| Traes_4AL_5A95E58C0.1 | XM 020296172.1 | hydroquinone glucosyltransferase-like (*Aegilops tauschii*) | 94% |
| Traes_4AL_5AC0AF8D3.1 | XM 020339333.1 | myosin-binding protein 3-like (*Aegilops tauschii*) | 97% |
| Traes_4AL_5BC6E4041.3 | XM 020337727.1 | protein CHUP1, chloroplastic-like (*Aegilops tauschii*) | 97% |
| Traes_4AL_5C7A4DA54.1 | KC286910.1 | phosphate starvation response 1 protein (*Aegilops tauschii*) | 88% |
| Traes_4AL_5CC914E5A.1 | XM 020301682.1 | oxalate oxidase GF-2.8 (*Aegilops tauschii*) | 95% |
| Traes_4AL_5D0C38C0D.1 | XM 020323135.1 | ankyrin repeat-containing protein At5g02620-like (*Aegilops tauschii*) | 80% |
| Traes_4AL_5D5A2058A.1 | AK333950.1 | N/A | 99% |
| Traes_4AL_5D452E078.1 | XM 020310490.1 | N/A | 98% |
| Traes_4AL_5D769AED8.1 | XM 020328428.1 | ruvB-like protein 1 (*Aegilops tauschii*) | 99% |
| Traes_4AL_5DE165102.1 | JN381555.1 | cytokinin oxidase/dehydrogenase (CKX2.4) gene （*Triticum aestivum*） | 84% |
| Traes_4AL_5E7F93445.2 | XM 020290612.1 | transcription factor TGAL4-like （*Aegilops tauschii*） | 95% |
| Traes_4AL_5E86DF14B.1 | XM 020310664.1 | transcription factor ERF094-like （*Aegilops tauschii*） | 83% |
| Traes_4AL_5E8588A20.2 | XM 020304697.1 | N/A | 99% |
| Traes_4AL_5EC714CAD.2 | XM 020300649.1 | beta-glucosidase 8-like （*Aegilops tauschii*） | 95% |
| Traes_4AL_5F2CDF7C7.1 | XM 020341038.1 | protein ECERIFERUM 26（*Aegilops tauschii*） | 87% |
| Traes_4AL_6A6EC9A6A.2 | XM 020333164.1 | transcription factor bHLH54-like （*Aegilops tauschii*） | 97% |
| Traes_4AL_6A62271F4.1 | XM 020300815.1 | N/A | 96% |
| Traes_4AL_6AAD7EED7.1 | XM 020298071.1 | N/A | 95% |
| Traes_4AL_6B5C89E7C.1 | XM 015775648.1 | 14-3-3-like protein GF14-F (*Oryza sativa*) | 93% |
| Traes_4AL_6B540C95C.1 | DQ915516.1 | ribosomal protein L3-B2 (*Triticum aestivum*) | 100% |
| Traes_4AL_6B088591C.2 | XM 020292004.1 | inositol-tetrakisphosphate 1-kinase 3 (*Aegilops tauschii* ) | 98% |
| Traes_4AL_6C73AD78E.1 | XM 020317409.1 | pentatricopeptide repeat-containing protein At3g12770-like (*Aegilops tauschii* ) | 95% |
| Traes_4AL_6C152CCAE.2 | XM 010239364.2 | adagio-like protein 3 ( *Brachypodium distachyon* ) | 89% |
| Traes_4AL_6DA775F02.1 | XM_020302662.1 | exocyst complex component SEC3A-like (*Aegilops tauschii* ) | 99% |
| Traes_4AL_6E51971E7.1 | AB182943.1 | Wknox1a gene for KN1 homeobox protein (*Triticum aestivum*) | 99% |
| Traes_4AL_6E5707721.1 | N/A | N/A |  |
| Traes_4AL_07DD1A9CD.1 | XM 020300328.1 | CCR4-NOT transcription complex subunit 11-like (*Aegilops tauschii*) | 99% |
| Traes_4AL_7A49D265B.1 | XM 020314299.1 | N/A | 97% |
| Traes_4AL_7C6CAB5FF.2 | XM 020299728.1 | F-box/kelch-repeat protein At2g44130-like (*Aegilops tauschii*) | 98% |
| Traes_4AL_7CC35DF1D.2 | EU157423.1 | haplotype A DNA repair protein Rad50 gene (*Triticum turgidum* ) | 97% |
| Traes_4AL_7D1F02B2F.2 | XM 020337418.1 | serine/threonine-protein kinase GRIK1-like (*Aegilops tauschii*) | 98% |
| Traes_4AL_7D8E33281.1 | XM 020323597.1 | N/A | 94% |
| Traes_4AL_7D9BCA397.1 | XM 020294024.1 | 26S proteasome non-ATPase regulatory subunit 10-like (*Aegilops tauschii*) | 97% |
| Traes_4AL_7D36AEDC8.1 | XM 020338611.1 | GPN-loop GTPase 3-like (*Aegilops tauschii*) | 98% |
| Traes_4AL_7D673DF48.1 | XM 020343558.1 | N/A | 94% |
| Traes_4AL_7DDCFABE9.1 | XM 020301719.1 | N/A | 98% |
| Traes_4AL_7E93C2ECA.1 | XM 020320384.1 | magnesium-dependent phosphatase 1 (*Aegilops tauschii*) | 98% |
| Traes_4AL_7EE57BE98.1 | XM 020316577.1 | DNA-(apurinic or apyrimidinic site) lyase (*Aegilops tauschii*) | 98% |
| Traes_4AL_7FFE0BBD5.1 | XM 020301726.1 | oxalate oxidase 1-like (*Aegilops tauschii*) | 97% |
| Traes_4AL_08D259134.1 | XM 020300328.1 | CCR4-NOT transcription complex subunit 11-like (*Aegilops tauschii*) | 100% |
| Traes_4AL_8B2D0DFD7.2 | XM 020337723.1 | N/A | 98% |
| Traes_4AL_8B52D54FD.2 | XM 020301019.1 | myb-related protein MYBAS1-like (*Aegilops tauschii*) | 99% |
| Traes_4AL_8BA51BBE8.2 | XM 020327775.1 | serine/threonine-protein kinase At1g54610 (*Aegilops tauschii*) | 98% |
| Traes_4AL_8BCE46958.1 | XM 020300098.1 | proteasome subunit alpha type-5-like (*Aegilops tauschii*) | 100% |
| Traes_4AL_8C4927CF5.3 | XM 020306582.1 | L-type lectin-domain containing receptor kinase IV.1-like (*Aegilops tauschii*) | 96% |
| Traes_4AL_8D6A610BE.1 | XR 002227961.1 | N/A | 99% |
| Traes_4AL_8E903F7A9.1 | XM 020321565.1 | receptor-like protein 2 (*Aegilops tauschii*) | 95% |
| Traes_4AL_8E8063A5D.1 | XM 020316602.1 | methylsterol monooxygenase 2-1 (*Aegilops tauschii*) | 99% |
| Traes_4AL_8E27959FD.2 | XM 014897819.1 | LRR receptor-like serine/threonine-protein kinase At1g05700 (*Brachypodium distachyon*) | 85% |
| Traes_4AL_8EE9280A6.1 | XM 020294025.1 | ankyrin-1-like (*Aegilops tauschii*) | 96% |
| Traes_4AL_8F26C77CE.1 | XM 020307086.1 | ascorbate-specific transmembrane electron transporter 2 （*Aegilops tauschii*） | 97% |
| Traes_4AL_8F0290B9D.1 | XM 020302004.1 | photosynthetic NDH subunit of subcomplex B 1, chloroplastic （*Aegilops tauschii*） | 97% |
| Traes_4AL_09CB42FFB.2 | XM 020300889.1 | N/A | 98% |
| Traes_4AL_9B18E4EAA.1 | N/A | N/A |  |
| Traes_4AL_9BCB37710.2 | XM 020310488.1 | glutamate decarboxylase 1-like (*Aegilops tauschii*) | 98% |
| Traes_4AL_9C61A876C.1 | XM 020310488.1 | glutamate decarboxylase 1-like (*Aegilops tauschii*) | 98% |
| Traes_4AL_9C2555C8A.1 | XM 020291994.1 | CSC1-like protein RXW8*(Aegilops tauschii*) | 98% |
| Traes_4AL_9D9DD9755.2 | XM 020339327.1 | outer envelope protein 64(*Aegilops tauschii*) | 97% |
| Traes_4AL_9D491EC88.2 | XM 020301005.1 | chaperone protein ClpB-like ( *Aegilops tauschii*) | 96% |
| Traes_4AL_9E250501B.2 | XM 020300854.1 | protein UPSTREAM OF FLC(*Aegilops tauschii*) | 95% |
| Traes_4AL_9FA155B6B.1 | XM 020300809.1 | N/A | 97% |
| Traes_4AL_9FC10033D.3 | XM 020314085.1 | N/A | 94% |
| Traes_4AL_10FAB334B.1 | AC063973.10 | T6F08 From Chromosome V( *Arabidopsis*) | 100% |
| Traes_4AL_11BB67057.2 | XM 020328394.1 | N/A | 95% |
| Traes_4AL_14E880659.1 | XM 020298613.1 | sec1 family domain-containing protein MIP3(*Aegilops tauschii*) | 98% |
| Traes_4AL_15E7E9D5F.1 | XM 020323338.1 | L10-interacting MYB domain-containing protein-like(*Aegilops tauschii*) | 97% |
| Traes_4AL_19EFE5ECC.1 | XM 020336004.1 | probable methyltransferase PMT2(*Aegilops tauschii*) | 96% |
| Traes_4AL_20CB03B12.2 | XM 020291997.1 | GDP-mannose transporter GONST1-like(*Aegilops tauschii*) | 96% |
| Traes_4AL_23A71AE94.1 | XM 020308684.1 | dirigent protein 1-like (*Aegilops tauschii*) | 94% |
| Traes_4AL_27EA1105D.2 | XM 020321570.1 | phosphatidylinositol 4-phosphate 5-kinase 1-like(*Aegilops tauschii*) | 97% |
| Traes_4AL_028BD4D07.1 | DQ469712.1 | kinase-like protein ABC1036 gene( *Hordeum vulgare*) | 86% |
| Traes_4AL_31A9169D8.1 | XM 020292247.1 | N/A | 97% |
| Traes_4AL_31C91123B.1 | AB028870.1 | Hv-MMT1 mRNA for S-adenosyl-L-methionine: L-methionine S-methyltransferase( *Hordeum vulgare*) | 78% |
| Traes_4AL_33BD28950.2 | XM 020345507.1 | GDSL esterase/lipase EXL3-like(*Aegilops tauschii*) | 87% |
| Traes_4AL_36D63D2CA.1 | KF926683.1 | alcohol-forming fatty acyl-coenzyme A reductase (FAR1) gene( *Hordeum vulgare*) | 99% |
| Traes_4AL_37B6D6FC3.1 | XM 020315140.1 | magnesium transporter MRS2-A, chloroplastic(*Aegilops tauschii*) | 98% |
| Traes_4AL_38B1ACFF0.5 | XM 020294059.1 | E3 ubiquitin-protein ligase RNF5-like(*Aegilops tauschii*) | 94% |
| Traes_4AL_39C161D66.2 | XM 020318037.1 | N/A | 96% |
| Traes_4AL_40E835A94.2 | XM 020310489.1 | transcription factor MYB52-like(*Aegilops tauschii*) | 96% |
| Traes_4AL_42FFE3197.1 | XM 020321566.1 | senescence-specific cysteine protease SAG39-like(*Aegilops tauschii*) | 94% |
| Traes_4AL_43C067DBB.2 | XM 020311212.1 | transcriptional corepressor SEUSS-like(*Aegilops tauschii*) | 97% |
| Traes_4AL_44C4C0B50.1 | XM 020320726.1 | zinc finger MYM-type protein 1(*Aegilops tauschii*) | 98% |
| Traes_4AL_44E99BA01.1 | KJ018715.1 | SNF1-type serine-threonine protein kinase (SnRK2.10) gene, SnRK2.10-A1a allele(*Triticum aestivum*) | 99% |
| Traes_4AL_44F8B7457.1 | XM 020335131.1 | phospholipase A1 EG1, chloroplastic/mitochondrial-like (*Aegilops tauschii*) | 96% |
| Traes_4AL_45EFB118E.2 | XM 020292665.1 | probable alkaline/neutral invertase F(*Aegilops tauschii*) | 99% |
| Traes_4AL_47E925781.1 | XM 020333029.1 | protein HIRA(*Aegilops tauschii*) | 99% |
| Traes_4AL_47F0C2746.1 | XM 020298869.1 | protein DETOXIFICATION 31-like( *Aegilops tauschii*) | 94% |
| Traes_4AL_48A716ECE.2 | XM 020339789.1 | zinc finger CCCH domain-containing protein 53-like(*Aegilops tauschii*) | 99% |
| Traes_4AL_49C03FD69.2 | XM 020340027.1 | thioredoxin-like protein AAED1, chloroplastic(*Aegilops tauschii*) | 95% |
| Traes_4AL_50F8115BB.1 | XM 020300827.1 | tauschii hydroxyproline O-galactosyltransferase GALT5-like(*Aegilops tauschii*) | 95% |
| Traes_4AL_50F8424FB.1 | XM 020293603.1 | N/A | 93% |
| Traes_4AL_52A7262C9.2 | XM 015762066.1 | lipase-like PAD4(*Oryza*) | 76% |
| Traes_4AL_52B53A5D2.1 | XM 010231163.2 | N/A | 90% |
| Traes_4AL_53B9504F1.1 | XM 020325552.1 | tauschii membrin-11-like(*Aegilops tauschii*) | 95% |
| Traes_4AL_53FFE6D9F.1 | XM 020345932.1 | ABC transporter G family member 11-like(*Aegilops tauschii*) | 86% |
| Traes_4AL_54EB84FD0.1 | XM 020325548.1 | dehydrin HIRD11(*Aegilops tauschii*) | 91% |
| Traes_4AL_54F25AA7C.2 | XM 020345481.1 | zinc finger BED domain-containing protein RICESLEEPER 2(*Aegilops tauschii*) | 99% |
| Traes_4AL_56CBD7646.1 | XM 020316739.1 | dolichol phosphate-mannose biosynthesis regulatory protein(*Aegilops tauschii*) | 97% |
| Traes_4AL_57F62E2EE.1 | XM 020340489.1 | F-box/kelch-repeat protein At2g44130-like(*Aegilops tauschii*) | 98% |
| Traes_4AL_58F89633A.1 | XM 014902181.1 | auxin-responsive protein IAA27-like(*Brachypodium distachyon*) | 95% |
| Traes_4AL_58FD04B85.2 | XM 020291769.1 | protein FAR1-RELATED SEQUENCE 5-like(*Aegilops tauschii*) | 98% |
| Traes_4AL_59B858338.2 | XM 020323697.1 | protein PLASTID MOVEMENT IMPAIRED 2-like(*Aegilops tauschii*) | 87% |
| Traes_4AL_59D5504B1.1 | XM 020331881.1 | protein NRT1/ PTR FAMILY 8.3-like(*Aegilops tauschii*) | 97% |
| Traes_4AL_62A3C8525.1 | XM 020316671.1 | tauschii RING-H2 finger protein ATL74-like(*Aegilops tauschii*) | 98% |
| Traes_4AL_64ACD4217.1 | XM 020310486.1 | N/A | 97% |
| Traes_4AL_64E8AC6A1.1 | XM 020302005.1 | eukaryotic translation initiation factor 3 subunit B-like(*Aegilops tauschii*) | 99% |
| Traes_4AL_66CEF3E24.2 | XM 020314078.1 | 4-coumarate--CoA ligase-like 7(*Aegilops tauschii*) | 97% |
| Traes_4AL_68B60F6AA.1 | XM 020343810.1 | protein FLOWERING LOCUS T-like(*Aegilops tauschii*) | 100% |
| Traes_4AL_69D61737F.1 | XM 020292670.1 | putative disease resistance RPP13-like protein 2 (*Aegilops tauschii*) | 95% |
| Traes_4AL_70F41757C.1 | XM 020304166.1 | N/A | 96% |
| Traes_4AL_72C3E1EAD.2 | JQ791299.1 | vulgare cultivar OWB-R PRR (PRR59) gene(*Hordeum vulgare*) | 91% |
| Traes_4AL_72DCF562.1 | XM 020311212.1 | transcriptional corepressor SEUSS-like(*Aegilops tauschii*) | 98% |
| Traes_4AL_72F31ECA8.1 | XM 020333541.1 | probable serine/threonine-protein kinase PBL25(*Aegilops tauschii*) | 98% |
| Traes_4AL_073E6E7A9.1 | AJ586805.1 | tua2 gene for alpha tubulin(*Setaria viridis*) | 99% |
| Traes_4AL_75D069945.1 | KX401585.1 | teosinte branched 1 protein (tb1) mRNA(*Triticum aestivum*) | 97% |
| Traes_4AL_75E7BE9EE.1 | XM 020338605.1 | mitogen-activated protein kinase kinase 9-like(*Aegilops tauschii*) | 94% |
| Traes_4AL_76D45DCBD.1 | XM 020298621.1 | putative auxin transporter-like protein 4(*Aegilops tauschii*) | 98% |
| Traes_4AL_77BEDA617.1 | XM 020314135.1 | N/A | 97% |
| Traes_4AL_078D4FC3C.1 | XM 020291484.1 | N/A | 95% |
| Traes_4AL_81EF65E93.2 | XM 020301011.1 | DNA (cytosine-5)-methyltransferase DRM2-like(*Aegilops tauschii*) | 98% |
| Traes_4AL_83DC54FD8.1 | XM 020323345.1 | pre-mRNA-splicing factor 18-like(*Aegilops tauschii*) | 95% |
| Traes_4AL_84DFF6A541.1 | XM 020337956.1 | mitogen-activated protein kinase kinase 9-like(Aegilops tauschii) | 96% |
| Traes_4AL_84E1F12C1.1 | XM 020336351.1 | N/A | 98% |
| Traes_4AL_85BCB2AF3.1 | XM 020312991.1 | flagellar radial spoke protein 5(*Aegilops tauschii*) | 96% |
| Traes_4AL_86DD80075.1 | XM 020309778.1 | tauschii uncharacterized LOC109750836(*Aegilops tauschii*) | 94% |
| Traes_4AL_87DA40FD5.2 | XM 020292675.1 | cysteine-rich receptor-like protein kinase 25(*Aegilops tauschii*) | 99% |
| Traes_4AL_87F6E077F.2 | XM 020345417.1 | putative pentatricopeptide repeat-containing protein At1g10330(*Aegilops tauschii*) | 96% |
| Traes_4AL_87F378BE8.1 | XM 020331715.1 | N/A | 92% |
| Traes_4AL_088D3E9A6.1 | XM 020336358.1 | receptor-like protein kinase HSL1(*Aegilops tauschii*) | 97% |
| Traes_4AL_89FFDAF71.1 | XM 020301009.1 | probable inactive receptor kinase At1g48480(*Aegilops tauschii*) | 97% |
| Traes_4AL_91D0D50DD1.1 | XM 020301739.1 | oxalate oxidase 2(*Aegilops tauschii*) | 92% |
| Traes_4AL_92A7B1118.1 | XM 020345440.1 | phenolic glucoside malonyltransferase 2-like(*Aegilops tauschii*) | 94% |
| Traes_4AL_95B0D2082.1 | XM 020290609.1 | N/A | 99% |
| Traes_4AL_95D685A1A.1 | XM 020307288.1 | probable beta-D-xylosidase 7(*Aegilops tauschii*) | 96% |
| Traes_4AL_95DD538FA.1 | XM 020316598.1 | tropinone reductase homolog At2g29290-like(*Aegilops tauschii*) | 98% |
| Traes_4AL_95FC2F534.4 | XM 020297606.1 | peptidyl-prolyl cis-trans isomerase CYP23-like(*Aegilops tauschii*) | 100% |
| Traes_4AL_96A137452.1 | XM 020333540.1 | E3 SUMO-protein ligase SIZ2-lik(*Aegilops tauschii*) | 98% |
| Traes_4AL_96E6567CA.1 | XM 020338614.1 | vam6/Vps39-like protein (*Aegilops tauschii*) | 99% |
| Traes_4AL_98B1C762B.1 | XM 020293740.1 | tauschii protein WRKY1-like(*Aegilops tauschii*) | 92% |
| Traes_4AL_140E3F2AC.4 | XM 020320822.1 | probably inactive leucine-rich repeat receptor-like protein kinase At5g48380(*Aegilops tauschii*) | 96% |
| Traes_4AL_142D09867.2 | XM 020291998.1 | ubiquitin thioesterase 3(*Aegilops tauschii*) | 98% |
| Traes_4AL_193A5756A.1 | XM 020337415.1 | DNA-directed RNA polymerase I subunit RPA12-like(*Aegilops tauschii*) | 100% |
| Traes_4AL_244DC5DFD.1 | XM 020301310.1 | protein OPAQUE10-like(*Aegilops tauschii*) | 96% |
| Traes_4AL_270CB8B2F.2 | XM 020292002.1 | N/A | 90% |
| Traes_4AL_272CFF824.1 | XM 020296190.1 | maf-like protein VV1_3015(*Aegilops tauschii*) | 95% |
| Traes_4AL_276CC5E27.1 | XM 020310479.1 | SWI/SNF complex subunit SWI3D(*Aegilops tauschii*) | 99% |
| Traes_4AL_328B98F9F.2 | XM 015760490.1 | putative receptor-like protein kinase At3g47110(*Oryza sativa*) | 73% |
| Traes_4AL_345A5A89F.2 | XM 020335083.1 | probable plastid-lipid-associated protein 4, chloroplastic(*Aegilops tauschii*) | 98% |
| Traes_4AL_392D11798.1 | XM 020327776.1 | glucuronoxylan 4-O-methyltransferase 3-like(*Aegilops tauschii*) | 96% |
| Traes_4AL_0443FBE18.2 | XM 020337418.1 | serine/threonine-protein kinase GRIK1-like(*Aegilops tauschii*) | 99% |
| Traes_4AL_482E04413.2 | XM 020325082.1 | putative disease resistance RPP13-like protein 3(*Aegilops tauschii*) | 91% |
| Traes_4AL_525B8BAA8.1 | XM 020335076.1 | serine/threonine-protein kinase mph1(*Aegilops tauschii*) | 99% |
| Traes_4AL_573E93FCC.1 | XM 020297438.1 | dof zinc finger protein DOF3.1-like(*Aegilops tauschii*) | 96% |
| Traes_4AL_596BAB8B9.1 | XM 020333354.1 | peroxidase 2-like(*Aegilops tauschii*) | 94% |
| Traes_4AL_597C42552.1 | XM 020301990.1 | chaperone protein ClpC2, chloroplastic-like(*Aegilops tauschii*) | 97% |
| Traes_4AL_680BF4C17.3 | XM 020303436.1 | protein JINGUBANG-like(*Aegilops tauschii*) | 95% |
| Traes_4AL_742EA24EC.2 | XM 016932046.1 | ArfGAP with dual PH domains 2(*Pan troglodytes*) | 97% |
| Traes_4AL_761BE525C.1 | XM 020307921.1 | PHD finger protein MALE MEIOCYTE DEATH 1-like(*Aegilops tauschii*) | 98% |
| Traes_4AL_761FA7038.1 | XM 020301008.1 | phosphatidylinositol 4-kinase alpha 1(*Aegilops tauschii*) | 99% |
| Traes_4AL_783D5A8DE.1 | XM 020292156.1 | zinc finger protein 7-like(*Aegilops tauschii*) | 94% |
| Traes_4AL_795BE6DDA.1 | LC089970.1 | spontaneum MKK3 gene for mitogen-activated protein kinase kinase 3(*Hordeum vulgare*) | 83% |
| Traes_4AL_828CDA9D0.1 | XR 002229012.1 | ubiquitin carboxyl-terminal hydrolase 12-like(*Aegilops tauschii*) | 100% |
| Traes_4AL_858CE90C8.2 | XM 020307686.1 | kininogen-1-like(*Aegilops tauschii*) | 95% |
| Traes_4AL_900AC3A03.2 | XM 020297934.1 | chlorophyll(ide) b reductase NOL, chloroplastic-like(*Aegilops tauschii*) | 99% |
| Traes_4AL_934E4EA576.1 | XM 020301014.1 | N/A | 95% |
| Traes_4AL_944EFB50F.2 | XM 020331232.1 | transcription factor HY5-like(*Aegilops tauschii*) | 92% |
| Traes_4AL_950BBBFA8.1 | XM 020309777.1 | cationic amino acid transporter 3, mitochondrial-like(*Aegilops tauschii*) | 93% |
| Traes_4AL_987BF5EF5.2 | AY691949.1 |  | 87% |
| Traes_4AL_1983DA267.2 | BK005645.1 | histidine-containing phosphotransfer protein 3(*Triticum aestivum*) | 98% |
| Traes_4AL_02072D986.1 | XM 020320388.1 | protein phosphatase inhibitor 2(*Aegilops tauschii*) | 96% |
| Traes_4AL_2475C298D.2 | MF285086.1 | fatty acyl-coenzyme A reductase (FAR3) gene(*Brachypodium distachyon*) | 82% |
| Traes_4AL_2656D85FD.2 | XM 020320554.1 | cysteine-rich receptor-like protein kinase 10(*Aegilops tauschii subsp*) | 93% |
| Traes_4AL_3193F9838.2 | XM 020325556.1 | protein PHR1-LIKE 3-like(*Aegilops tauschii*) | 97% |
| Traes_4AL_3216C08CD.1 | XM 020321561.1 | putative UDP-rhamnose:rhamnosyltransferase 1(*Aegilops tauschii*) | 92% |
| Traes_4AL_3339E4870.1 | XM 020318083.1 | microsomal glutathione S-transferase 3-like(*Aegilops tauschii*) | 97% |
| Traes_4AL_3477AAC0B.1 | XM 020316625.1 | thaumatin-like protein(*Aegilops tauschii*) | 99% |
| Traes_4AL_3505A065D.1 | XM_020310926.1 | N/A | 97% |
| Traes_4AL_3742AB62C.1 | AY320037.1 | RNA binding protein Rp120 gene(*Oryza sativa*) | 79% |
| Traes_4AL_04307A116.2 | XM 020301309.1 | mitogen-activated protein kinase kinase kinase 1-like(*Aegilops tauschii*) | 98% |
| Traes_4AL_4538C7F1E.1 | KT168389.1 | voucher DEK:M.R. Duvall:s.n. plastid(*Sporobolus heterolepis*) | 96% |
| Traes_4AL_4576D39A6.1 | XM_020327523.1 | protein YLS3-like(*Aegilops tauschii*) | 92% |
| Traes_4AL_4628E2B0C.6 | XM_020320074.1 | probable aquaporin PIP2-7 (*Aegilops tauschii*) | 94% |
| Traes_4AL_5147AF747.1 | XM_020331265.1 | UDP-glucose 6-dehydrogenase 4-like(*Aegilops tauschii*) | 99% |
| Traes_4AL_5296B44A2.1 | XM 020301006.1 | zinc finger protein CONSTANS-LIKE 16-like(*Aegilops tauschii*) | 98% |
| Traes_4AL_5319DD7C1.2 | XM_020337661.1 | hydroxypyruvate reductase-like(*Aegilops tauschii*) | 98% |
| Traes_4AL_6004CB00C.1 | XM 020329381.1 | N/A | 79% |
| Traes_4AL_6240D6D66.1 | XM 020332052.1 | folate transporter 1, chloroplastic(*Aegilops tauschii*) | 97% |
| Traes_4AL_6252B0BA0.2 | XM_020292179.1 | cold-regulated 413 inner membrane protein 1, chloroplastic-like(*Aegilops tauschii*) | 95% |
| Traes_4AL_6624EBA5F.2 | XM 020301008.1 | phosphatidylinositol 4-kinase alpha 1 (*Aegilops tauschii*) | 99% |
| Traes_4AL_6770A8F27.1 | XM 020306402.1 | FIP1[V]-like protein(*Aegilops tauschii*) | 99% |
| Traes_4AL_7264C5DB2.2 | \|XR_001544742.1\| | serine carboxypeptidase-like 2 (*Oryza sativa*) | 87% |
| Traes_4AL_7468B642E.1 | XM_020316576.1 | ABC transporter C family member 10-like(*Aegilops tauschii*) | 98% |
| Traes_4AL_7826B78AE.1 | XM 020302180.1 | MLO-like protein 4(*Aegilops tauschii*) | 96% |
| Traes_4AL_8224C29A9.2 | XM 020302174.1 | SWI/SNF complex subunit SWI3C-like(*Aegilops tauschii*) | 98% |
| Traes_4AL_8256E3FCE.1 | XM_020294027.1 | ankyrin repeat domain-containing protein 17-like(*Aegilops tauschii*) | 95% |
| Traes_4AL_8845F411B.1 | XM 020304359.1 | UDP-glucose 6-dehydrogenase 4(*Aegilops tauschii*) | 98% |
| Traes_4AL_9345F41571.1 | XM 020339823.1 | F-box protein At1g55000(*Aegilops tauschii*) | 95% |
| Traes_4AL_9623B2FCD.1 | HG670306.1 | chromosome 3B, genomic scaffold(*Triticum aestivum*) | 90% |
| Traes_4AL_10852CECB.1 | HG670306.1 | chromosome 3B, genomic scaffold(*Triticum aestivum*) | 90% |
| Traes_4AL_18696DF2E.2 | XM 020317408.1 | protein transport protein SEC23-like(*Aegilops tauschii*) | 98% |
| Traes_4AL_20224E98A.2 | XM 020314073.1 | pentatricopeptide repeat-containing protein At5g55840(*Aegilops tauschii*) | 97% |
| Traes_4AL_23624E9EA.2 | XM 020299502.1 | cell number regulator 13-like(*Aegilops tauschii*) | 97% |
| Traes_4AL_29542DF45.1 | XM 020316612.1 | caffeoylshikimate esterase-like(*Aegilops tauschii*) | 97% |
| Traes_4AL_40046CAEE.1 | XM 020324392.1 | N/A | 99% |
| Traes_4AL_45413B08D.1 | XM 020320897.1 | translation initiation factor IF-2-like(*Aegilops tauschii*) | 89% |
| Traes_4AL_53360CB14.2 | XM 020292678.1 | cysteine-rich receptor-like protein kinase 40(*Aegilops tauschii*) | 88% |
| Traes_4AL_080706A7E.1 | XM_020295848.1 | protein BZR1 homolog 1(*Aegilops tauschii*) | 91% |
| Traes_4AL_96068A41E.1 | XM_020337725.1 | nuclear pore complex protein NUP98A-like(*Aegilops tauschii*) | 98% |
| Traes_4AL_96105BEBB.1 | XM 020327522.1 | protein MARD1(*Aegilops tauschii*) | 94% |
| Traes_4AL_0174250EB.1 | XM 020327521.1 | N/A | 96% |
| Traes_4AL_288923C66.1 | XM 020339323.1 | N/A | 88% |
| Traes_4AL_289715EA4.1 | XM 020336240.1 | COMPASS-like H3K4 histone methylase component WDR5A(*Aegilops tauschii*) | 99% |
| Traes_4AL_291072FA8.1 | XM 020291992.1 | EPIDERMAL PATTERNING FACTOR-like protein 2(*Aegilops tauschii*) | 93% |
| Traes_4AL_388619BDE.1 | XM 020339823.1 | F-box protein At1g55000(*Aegilops tauschii*) | 99% |
| Traes_4AL_429683D72.1 | XM 020338605.1 | mitogen-activated protein kinase kinase 9-like(*Aegilops tauschii subsp*) | 96% |
| Traes_4AL_1436629AB.1 | XM 020325547.1 | ankyrin repeat domain-containing protein 65-like(*Aegilops tauschii*) | 99% |
| Traes_4AL_1794294B0.3 | XM_020301689.1 | lysine--tRNA ligase-like(*Aegilops tauschii*) | 98% |
| Traes_4AL_2416286BE.1 | XM 020339827.1 | adenylosuccinate synthetase 2, chloroplastic(*Aegilops tauschii*) | 94% |
| Traes_4AL_5275446F5.1 | XM 010233569.2 | methionine S-methyltransferase(*Brachypodium distachyon*) | 87% |
| Traes_4AL_5659286D11.1 | XM_020312885.1 | zinc-finger homeodomain protein 11-like(*Aegilops tauschii*) | 97% |
| Traes_4AL_7241716B6.1 | XM 020321806.1 | LOB domain-containing protein 39-like(*Aegilops tauschii*) | 97% |
| Traes_4AL_7312081FA.1 | XM 020307920.1 | 26S proteasome non-ATPase regulatory subunit 8 homolog A-like(*Aegilops tauschii*) | 100% |
| Traes_4AL_8907101BD.1 | XR 002229761.1 | N/A | 87% |
| Traes_4AL_41666607C.1 | XM 020301000.1 | N/A | 96% |
| Traes_4AL_50918719B.1 | XM 020339204.1 | probable serine/threonine-protein kinase PBL8(*Aegilops tauschii* ) | 98% |
| Traes_4AL_179419852.1 | KY636120.1 | voucher PI 236672 chloroplast(*Elymus spicatus*) | 97% |
| Traes_4AL_217375482.2 | XM 020320382.1 | E3 ubiquitin-protein ligase HOS1(*Aegilops tauschii*) | 98% |
| Traes_4AL_257914351.2 | XM 020291483.1 | hemolysin A(*Aegilops tauschii*) | 89% |
| Traes_4AL_949964316.2 | XR_002227959.1 | N/A | 96% |
| Traes_4AL_966704382.2 | XM 020294344.1 | N/A | 96% |
| Traes_4AL_A0A015B35.1 | XM 020339817.1 | multicopper oxidase LPR1-like(*Aegilops tauschii*) | 92% |
| Traes_4AL_A1D089370.1 | XM 020293991.1 | NADH dehydrogenase [ubiquinone] iron-sulfur protein 1, mitochondrial(*Aegilops tauschii*) | 96% |
| Traes_4AL_A2B0A2263.1 | XM 020321807.1 | acylpyruvase FAHD1, mitochondrial-like(*Aegilops tauschii*) | 100% |
| Traes_4AL_A3E8FBDF1.1 | XM 020315352.1 | N/A | 88% |
| Traes_4AL_A5AC2BDF2.2 | XM_020339825.1 | probable linoleate 9S-lipoxygenase 4(*Aegilops tauschii*) | 99% |
| Traes_4AL_A5B9F7B36.1 | XM 020301217.1 | 1-aminocyclopropane-1-carboxylate synthase 1-like(*Aegilops tauschii*) | 98% |
| Traes_4AL_A5BC0BDBA.2 | XM 020294023.1 | BEL1-like homeodomain protein 3(*Aegilops tauschii*) | 94% |
| Traes_4AL_A7C5368A9.2 | XM_020323344.1 | importin-5-like(*Aegilops tauschii*) | 99% |
| Traes_4AL_A7DEE5A47.2 | XM 020303837.1 | ER membrane protein complex subunit 10(*Aegilops tauschii*) | 96% |
| Traes_4AL_A7E5F01FE.1 | XM_020307085.1 | probable histidine kinase 4(*Aegilops tauschii*) | 97% |
| Traes_4AL_A8EB51E0B.1 | XM 020323342.1 | N/A | 100% |
| Traes_4AL_A11C2A4B1.2 | XM 020294336.1 | cytochrome P450 714C3-like(*Aegilops tauschii*) | 98% |
| Traes_4AL_A16A981D6.1 | XM 020293993.1 | N/A | 95% |
| Traes_4AL_A22E3977B1.1 | XM_020329452.1 | UDP-glucose 6-dehydrogenase 2(*Aegilops tauschii*) | 98% |
| Traes_4AL_A66FC5FEA.1 | XM 020321560.1 | pinin(*Aegilops tauschii*) | 95% |
| Traes_4AL_A236F632E.2 | XM 020333321.1 | non-lysosomal glucosylceramidase(*Aegilops tauschii*) | 99% |
| Traes_4AL_A345D7FAD.1 | XM 020334328.1 | N/A | 97% |
| Traes_4AL_A437BAD23.1 | XM 020319852.1 | homeobox protein knotted-1-like 4(*Aegilops tauschii*) | 98% |
| Traes_4AL_A53434A72.1 | AC133334.8 | chromosome 3 BAC OSJNBa0004L11 genomic sequence(*Oryza sativa*) | 96% |
| Traes_4AL_A63352E3A.1 | XM 020304289.1 | protein transport protein SEC31-like(*Aegilops tauschii*) | 93% |
| Traes_4AL_A66747A8F1.1 | KY636112.1 | voucher PI 598613 chloroplast(*Psathyrostachys juncea*) | 100% |
| Traes_4AL_A92657C6E.2 | XM 020323171.1 | phosphoglucomutase, cytoplasmic(*Aegilops tauschii*) | 99% |
| Traes_4AL_A93675BD6.2 | KJ001485.1 | genotype HW4022 ethylene receptor 1(*Triticum aestivum*) | 100% |
| Traes_4AL_A8188635C.2 | XM 020311902.1 | BTB/POZ domain-containing protein NPY4-like (*Aegilops tauschii*) | 98% |
| Traes_4AL_A38047286.1 | XM 020302662.1 | exocyst complex component SEC3A-like(*Aegilops tauschii*) | 99% |
| Traes_4AL_A61397455.2 | XR 002230492.1 | N/A | 90% |
| Traes_4AL_AA06C6037.2 | XM 020325880.1 | disease resistance protein RPM1-like(*Aegilops tauschii*) | 98% |
| Traes_4AL_AA326BB25.1 | XM 020307463.1 | putative pentatricopeptide repeat-containing protein At3g28640(*Aegilops tauschii*) | 95% |
| Traes_4AL_AAB70FF2D.1 | XM_020339824.1 | linoleate 9S-lipoxygenase 1( *Aegilops tauschii*) | 95% |
| Traes_4AL_AAF133B4E.1 | XM 020300800.1 | N/A | 99% |
| Traes_4AL_AB3C62E5A.1 | XM_020298072.1 | protein STICHEL-like(*Aegilops tauschii* ) | 99% |
| Traes_4AL_AB45022BD.1 | XM 020300331.1 | serine carboxypeptidase-like 19(*Aegilops tauschii* ) | 93% |
| Traes_4AL_AC322634F.1 | XM 020294043.1 | serine/arginine-rich SC35-like splicing factor SCL30(*Aegilops tauschii*) | 97% |
| Traes_4AL_AC47243193.3 | XM 020320424.1 | At2g13600 | 97% |
| Traes_4AL_AD17CDDAD.1 | XM 020291481.1 | ankyrin repeat protein SKIP35-like(*Aegilops tauschii*) | 99% |
| Traes_4AL_AEEF11733.2 | XR 002225922.1 | N/A | 83% |
| Traes_4AL_AEF07FE57.1 | XM 020333324.1 | probable protein phosphatase 2C 74(*Aegilops tauschii*) | 96% |
| Traes_4AL_AF23ECF39.2 | XM 020329447.1 | N/A | 97% |
| Traes_4AL_AFCE21C65.2 | XM_020292008.1 | putative lipase ROG1(*Aegilops tauschii*) | 99% |
| Traes_4AL_B0B1A06D3.1 | XM 020314087.1 | 4-coumarate--CoA ligase-like 7(*Aegilops tauschii*) | 96% |
| Traes_4AL_B0C867A5B.1 | XM 020292825.1 | GRF1-interacting factor 1(*Aegilops tauschii*) | 97% |
| Traes_4AL_B3EE1FFDC.1 | XM 020316613.1 | THO complex subunit 4B-like (*Aegilops tauschii*) | 97% |
| Traes_4AL_B4D6FEA07.2 | XM_020305534.1 | protein OSB1, mitochondrial-like(*Aegilops tauschii*) | 98% |
| Traes_4AL_B4D64A274.1 | XM 020301995.1 | tetrapyrrole-binding protein, chloroplastic(*Aegilops tauschii*) | 93% |
| Traes_4AL_B5DA1307A.1 | XM 020300997.1 | protein CASP(*Aegilops tauschii*) | 100% |
| Traes_4AL_B7C432896.2 | XM_020338602.1 | mitogen-activated protein kinase kinase 9-like (*Aegilops tauschii*) | 95% |
| Traes_4AL_B9AC4BB96.1 | AK331493.1 | cDNA, clone: WT007_K04, cultivar(*Triticum aestivum*) | 100% |
| Traes_4AL_B17B1C204.1 | XM_020319606.1 | N/A | 98% |
| Traes_4AL_B44DF4ACC.1 | XM 020326146.1 | protein CUP-SHAPED COTYLEDON 2-like(*Aegilops tauschii*） | 96% |
| Traes_4AL_B68B1EDE7.1 | XM_020332326.1 | ELMO domain-containing protein A-like(*Aegilops tauschii*) | 98% |
| Traes_4AL_B68D646B2.1 | XM 020305164.1 | probable protein S-acyltransferase 6( *Aegilops tauschii*) | 97% |
| Traes_4AL_B79EFF920.2 | XM 020301724.1 | N/A | 98% |
| Traes_4AL_B90EFBE3E.2 | XM 020291364.1 | F-box/FBD/LRR-repeat protein At5g22660-like(*Aegilops tauschii*) | 97% |
| Traes_4AL_B0395ACE3.1 | XM 020292002.1 | N/A | 94% |
| Traes_4AL_B587AADD1.1 | XM 020325547.1 | ankyrin repeat domain-containing protein 65-like (*Aegilops tauschii*) | 95% |
| Traes_4AL_B616E1A74.1 | XM 020304969.1 | gamma-glutamyl peptidase 5-like(*Aegilops tauschii*) | 96% |
| Traes_4AL_B674B528F.1 | XM 020316175.1 | N/A | 98% |
| Traes_4AL_B730CAA47.1 | XM_020310778.1 | calcium-transporting ATPase 3(*Aegilops tauschii*) | 100% |
| Traes_4AL_B808CA890.2 | XM 020297097.1 | zinc finger protein ZAT11-like(*Aegilops tauschii*) | 98% |
| Traes_4AL_B6992AAA6.1 | XM 020332051.1 | GATA transcription factor 19-like(*Aegilops tauschii*) | 96% |
| Traes_4AL_B21763AAA.1 | XM 020339818.1 | N-alpha-acetyltransferase 20 (*Aegilops tauschii*) | 98% |
| Traes_4AL_B88891C7A.1 | XM 020301728.1 | protein yippee-like(*Aegilops tauschii*) | 98% |
| Traes_4AL_B970181CE.1 | XM 020307464.1 | E3 ubiquitin-protein ligase MIEL1-like(*Aegilops tauschii*) | 97% |
| Traes_4AL_B32330729.1 | XM 020339783.1 | syntaxin-related protein KNOLLE(*Aegilops tauschii*) | 98% |
| Traes_4AL_BA88ED79E.1 | FJ952565.1 | clone pStC1 centromere-associated sequence(*Pseudoroegneria spicata*) | 76% |
| Traes_4AL_BC6B73F60.1 | XM 020315742.1 | phosphatidylinositol 4-phosphate 5-kinase 1-like(*Aegilops tauschii*) | 96% |
| Traes_4AL_BC54910D5.2 | XM 020324264.1 | zinc-finger homeodomain protein 4-like(*Aegilops tauschii*) | 93% |
| Traes_4AL_BCBC1AFFB.1 | KF282629.1 | cultivar Chinese Spring clone BAC 351D1 chromosome 4A DEL(*Triticum aestivum*) | 99% |
| Traes_4AL_BDDA9E30C.1 | XM 020301250.1 | mitochondrial import receptor subunit TOM6 homolog(*Aegilops tauschii*) | 99% |
| Traes_4AL_BE1AD302B.1 | XM 020302262.1 | 30S ribosomal protein S5-like(*Aegilops tauschii*) | 99% |
| Traes_4AL_BE80FCB64.1 | XM 020337414.1 | homologous-pairing protein 2 homolog(*Aegilops tauschii*) | 99% |
| Traes_4AL_BE405B578.1 | XM 020305144.1 | two pore potassium channel b-like(*Aegilops tauschii*) | 95% |
| Traes_4AL_BE2529B7A.1 | XM 020327650.1 | N/A | 94% |
| Traes_4AL_BF0D3FF35.1 | XM 020302010.1 | dehydrogenase/reductase SDR family member 7(*Aegilops tauschii*) | 93% |
| Traes_4AL_BF788C5DA.1 | XM 020301010.1 | cell division cycle-associated protein 7-like(*Aegilops tauschii*) | 97% |
| Traes_4AL_BFAB568BF.1 | KP844889.1 | Chinese Spring PM19-A1 gene(*Triticum aestivum* ) | 99% |
| Traes_4AL_C2A08A56A.2 | XM 020335087.1 | GEM-like protein 1(*Aegilops tauschii*) | 98% |
| Traes_4AL_C2DAF8C4F.2 | XM 020331697.1 | ACT domain-containing protein ACR3-like(*Aegilops tauschii*) | 97% |
| Traes_4AL_C3FEE3FC8.1 | XM 020298070.1 | 21.9 kDa heat shock protein-like(*Aegilops tauschii*) | 94% |
| Traes_4AL_C05CDAE1A.1 | XM 020301315.1 | zinc finger protein 593(*Aegilops tauschii*) | 98% |
| Traes_4AL_C5D6FF030.1 | XM_020337728.1 | G8 domain-containing protein DDB_G0286311-like(*Aegilops tauschii*) | 98% |
| Traes_4AL_C6ADF44E1.1 | XM 020312990.1 | monodehydroascorbate reductase-like(*Aegilops tauschii*) | 98% |
| Traes_4AL_C7E73E827.1 | XM_020302180.1 | MLO-like protein 4 (*Aegilops tauschii*) | 96% |
| Traes_4AL_C8C085E081.1 | XM_020301298.1 | NDR1/HIN1-like protein 13 (*Aegilops tauschii*) | 96% |
| Traes_4AL_C8FEA97C.1 | XM_020340977.1 | probable U3 small nucleolar RNA-associated protein 7(*Aegilops tauschii*) | 98% |
| Traes_4AL_C9FA4E734.1 | AC079736.13 | Nipponbare chromosome 3 clone OSJNBa0003G23(*Oryza sativa*) | 80% |
| Traes_4AL_C35DFAE33.1 | XM_020297358.1 | shewanella-like protein phosphatase 2(*Aegilops tauschii*) | 98% |
| Traes_4AL_C36A3933F.1 | XM 020337422.1 | probable serine/threonine protein kinase IREH1(*Aegilops tauschii*) | 99% |
| Traes_4AL_C060BF9F6.3 | XM 020305539.1 | LOB domain-containing protein 6-like(*Aegilops tauschii*) | 97% |
| Traes_4AL_C73F0C2A5.1 | XM 020316714.1 | probable pre-mRNA-splicing factor ATP-dependent RNA helicase DEAH4(*Aegilops tauschii*) | 98% |
| Traes_4AL_C93A36B92.1 | NULL | N/A |  |
| Traes_4AL_C362DF8E1.1 | AC135228.5 | chromosome 3 BAC OSJNBb0122C16 genomic sequence(*Oryza sativa*) | 86% |
| Traes_4AL_C428C4DF6.1 | XM 020310773.1 | serine carboxypeptidase-like 13(*Aegilops tauschii*) | 88% |
| Traes_4AL_C1663E48F.1 | XM 020301000.1 | N/A | 99% |
| Traes_4AL_C7393B692.1 | XM_015773948.1 | N/A | 78% |
| Traes_4AL_C39245F02.2 | XM 020316749.1 | exosome complex component RRP43(*Aegilops tauschii*) | 96% |
| Traes_4AL_C54588D09.1 | XM 020317850.1 | protein ZINC INDUCED FACILITATOR-LIKE 1-like(*Aegilops tauschii*) | 98% |
| Traes_4AL_C0862001E.2 | XM 020302660.1 | ATP-dependent (S)-NAD(P)H-hydrate dehydratase(*Aegilops tauschii*) | 95% |
| Traes_4AL_C902907E9.1 | XM_020331911.1 | serine/threonine-protein kinase STE20-like(*Aegilops tauschii*) | 98% |
| Traes_4AL_C30406196.2 | AC087797.5 | chromosome 3 BAC OSJNBb0022E02 genomic sequence(*Oryza sativa*) | 80% |
| Traes_4AL_CA5DFDE92.1 | XM 020339334.1 | probable transcription factor At3g04930(*Aegilops tauschii*) | 97% |
| Traes_4AL_CB49560F4.1 | XM 020301727.1 | putative ABC1 protein At2g40090(*Aegilops tauschii*) | 94% |
| Traes_4AL_CB2996256.1 | XM 020326080.1 | type IV inositol polyphosphate 5-phosphatase 11(*Aegilops tauschii*) | 97% |
| Traes_4AL_CD55FD398.2 | XM_020333030.1 | GDSL esterase/lipase At5g03610-like(*Aegilops tauschii*) | 95% |
| Traes_4AL_CE6489E9E.1 | XM 020343267.1 | N/A | 96% |
| Traes_4AL_CED469CC5.2 | XM 020298631.1 | N/A | 97% |
| Traes_4AL_CF9DF337F.1 | XM 020314136.1 | elongation of fatty acids protein 3-like(*Aegilops tauschii*) | 96% |
| Traes_4AL_CF6130092.1 | XM 020298575.1 | leucine-rich repeat receptor protein kinase MSL1-like(*Aegilops tauschii*) | 98% |
| Traes_4AL_CFEC9B74A.1 | XM 020341220.1 | protein FAF-like, chloroplastic(*Aegilops tauschii*) | 92% |
| Traes_4AL_D0A843665.1 | XM 020317849.1 | glycerol-3-phosphate 2-O-acyltransferase 6-like(*Aegilops tauschii*) | 97% |
| Traes_4AL_D0DECE300.2 | XM 020333162.1 | probable 2-oxoglutarate-dependent dioxygenase At5g05600(*Aegilops tauschii*) | 98% |
| Traes_4AL_D1B3210E0.2 | XM 020303197.1 | N/A | 95% |
| Traes_4AL_D2D7703821.2 | XM 020338977.1 | ethylene-responsive transcription factor ERF022-like(*Aegilops tauschii*) | 99% |
| Traes_4AL_D3AD24DF3.1 | XM 020304756.1 | protein SHORTAGE IN CHIASMATA 1-like(*Aegilops tauschii*) | 98% |
| Traes_4AL_D4BDE3DE7.1 | XM 020307217.1 | NRT1/ PTR FAMILY 7.3-like(*Aegilops tauschii*) | 99% |
| Traes_4AL_D6A7C5CE7.1 | XM 020306402.1 | FIP1[V]-like protein(*Aegilops tauschi*i) | 98% |
| Traes_4AL_D9B9FDD2C.2 | HG670306.1 | chromosome 3B, genomic scaffold, cultivar Chinese Spring(*Triticum aestivum* ) | 92% |
| Traes_4AL_D41A9CCB8.2 | XM 020345035.1 | N/A | 98% |
| Traes_4AL_D68C3FE3B.1 | XM 020321037.1 | protein NUCLEAR FUSION DEFECTIVE 4-like (*Aegilops tauschii*) | 95% |
| Traes_4AL_D70AE8BE9.1 | AB753271.1 | TRIae;Pht1;12 mRNA for phosphate transporter(*Triticum aestivum*) | 99% |
| Traes_4AL_D92A1E1A7.1 | XM 020333030.1 | GDSL esterase/lipase At5g03610-like(*Aegilops tauschii*) | 93% |
| Traes_4AL_D294B0B60.2 | XM 020292571.1 | disease resistance protein RPM1-like(*Aegilops tauschii*) | 97% |
| Traes_4AL_D435B7DFB.1 | XM 003577336.3 | HVA22-like protein(*Brachypodium distachyon*) | 91% |
| Traes_4AL_D971AF253.2 | XM 020307435.1 | N/A | 96% |
| Traes_4AL_D026163B1.2 | XM 020302186.1 | protein CELLULOSE SYNTHASE INTERACTIVE 3(*Aegilops tauschii*) | 99% |
| Traes_4AL_D654726DA.1 | XM 020291477.1 | putative protease Do-like 14(*Aegilops tauschii*) | 99% |
| Traes_4AL_D25269085.2 | XM 020339335.1 | zinc finger protein VAR3, chloroplastic-like(*Aegilops tauschii*) | 97% |
| Traes_4AL_DA2BA70B1.3 | XM 020308080.1 | type IV inositol polyphosphate 5-phosphatase 7-like(*Aegilops tauschii*) | 96% |
| Traes_4AL_DA9C64489.1 | XM_020298993.1 | monoacylglycerol lipase-like(*Aegilops tauschii*) | 97% |
| Traes_4AL_DA093AE07.1 | XM 020310775.1 | lecithin-cholesterol acyltransferase-like 1(*Aegilops tauschii*) | 95% |
| Traes_4AL_DAB6ACB13.1 | XM 020316576.1 | ABC transporter C family member 10-like(*Aegilops tauschii*) | 98% |
| Traes_4AL_DB796F1F1.2 | AB182943.1 | Wknox1a gene for KN1 homeobox protein(*Triticum aestivum*) | 99% |
| Traes_4AL_DB38630BD.1 | XM 020314088.1 | mitochondrial import receptor subunit TOM5 homolog(*Aegilops tauschii*) | 98% |
| Traes_4AL_DCE84CDD3.1 | XM 020303430.1 | exocyst complex component SEC3A-like(*Aegilops tauschii*） | 98% |
| Traes_4AL_DDA45F0401.2 | XM 020341243.1 | N/A | 90% |
| Traes_4AL_DE6A7FB56.1 | KU570057.1 | G3116 GRIK1-like protein gene(*Triticum monococcum*) | 85% |
| Traes_4AL_DE6DA7E39.1 | XM 020321656.1 | probable sucrose-phosphate synthase 5(*Aegilops tauschii*） | 97% |
| Traes_4AL_DEF671AF7.1 | XM_020301158.1 | beta-adaptin-like protein C(*Aegilops tauschii* ) | 98% |
| Traes_4AL_DF6BEB17A.3 | XM 020335331.1 | CTD kinase subunit alpha-like(*Aegilops tauschii*) | 83% |
| Traes_4AL_DF50540B4.2 | XM 020338113.1 | CCR4-NOT transcription complex subunit 3(*Aegilops tauschii*) | 97% |
| Traes_4AL_DFFEABB2B.1 | XM 020304752.1 | N/A | 96% |
| Traes_4AL_E1A45DF73.2 | \|XM_020318086.1\| | microsomal glutathione S-transferase 3-like(*Aegilops tauschii*) | 98% |
| Traes_4AL_E1E33D866.2 | XM 020317410.1 | protein FAM136A-like (*Aegilops tauschii*) | 98% |
| Traes_4AL_E2BACF604.1 | XM 020310487.1 | histone H2AX(*Aegilops tauschii*) | 96% |
| Traes_4AL_E6A7626AD.1 | XM 020339329.1 | N/A | 97% |
| Traes_4AL_E9A559951.2 | XM 020302006.1 | N/A | 96% |
| Traes_4AL_E9F963D47.1 | XM 020310928.1 | protein BPS1, chloroplastic-like(*Aegilops tauschii*) | 96% |
| Traes_4AL_E9FD810DB.2 | XM 020301994.1 | endonuclease III homolog 1, chloroplastic-like(*Aegilops tauschii*) | 99% |
| Traes_4AL_E35BCBB0D.2 | XM 020301713.1 | N/A | 96% |
| Traes_4AL_E62F45D521.1 | XM 020307087.1 | pentatricopeptide repeat-containing protein At2g37230-like(*Aegilops tauschii*) | 96% |
| Traes_4AL_E72E43FED.1 | XM 020332509.1 | N/A | 97% |
| Traes_4AL_E80FAEC2B.2 | XM 020337420.1 | coatomer subunit alpha-3(*Aegilops tauschii*) | 98% |
| Traes_4AL_E95E68A9C.1 | XM 020331939.1 | probable protein kinase At2g41970(*Aegilops tauschii*) | 95% |
| Traes_4AL_E5490C0D0.1 | XM 020302665.1 | 40S ribosomal protein S27(*Aegilops tauschii*) | 96% |
| Traes_4AL_E7396A416.2 | XM 020298867.1 | protein DETOXIFICATION 29-like(*Aegilops tauschii* ) | 97% |
| Traes_4AL_E57587E16.1 | XM 020338458.1 | transcription factor PCF3-like(*Aegilops tauschii*) | 99% |
| Traes_4AL_E82666A4B.2 | XM 020335082.1 | histone-lysine N-methyltransferase ASHR1(*Aegilops tauschii*) | 98% |
| Traes_4AL_E487438FD.1 | XM 020336827.1 | peptide chain release factor APG3, chloroplastic(*Aegilops tauschii*) | 99% |
| Traes_4AL_E38110453.1 | XM 020335088.1 | ATP synthase delta chain, chloroplastic-like(*Aegilops tauschii*) | 96% |
| Traes_4AL_E93614021.1 | XM 020306407.1 | 60S ribosomal protein L6, mitochondrial-like(*Aegilops tauschii*) | 96% |
| Traes_4AL_EAAA8D2DB.1 | XM 020301719.1 | N/A | 98% |
| Traes_4AL_EB7B7633A.1 | XM 020302186.1 | protein CELLULOSE SYNTHASE INTERACTIVE 3(*Aegilops tauschii*) | 98% |
| Traes_4AL_EBC32CD49.1 | XM 020341254.1 | 60S ribosomal protein L10-1(*Aegilops tauschii* ) | 99% |
| Traes_4AL_ECCF59611.1 | XM 020325969.1 | cation/calcium exchanger 1-like(*Aegilops tauschii* ) | 94% |
| Traes_4AL_EDFBD3F2B.3 | XM 020311548.1 | F-box/LRR-repeat protein 4(*Aegilops tauschii*) | 97% |
| Traes_4AL_EE0B0770C.1 | XM 020338610.1 | binding partner of ACD11 1(*Aegilops tauschii*) | 98% |
| Traes_4AL_EE6B9F885.1 | XM 020318183.1 | glucan endo-1,3-beta-glucosidase 8-like(*Aegilops tauschii*) | 97% |
| Traes_4AL_EF34E6286.1 | XM 020293741.1 | N/A | 99% |
| Traes_4AL_F1AF81647.2 | XM 020305137.1 | protein FAR1-RELATED SEQUENCE 5-like(*Aegilops tauschii*） | 97% |
| Traes_4AL_F1D8BC8D11.3 | XM 020322704.1 | CBL-interacting protein kinase 6-like(*Aegilops tauschii* ) | 98% |
| Traes_4AL_F2C96B4E2.1 | NULL | N/A |  |
| Traes_4AL_F3BA3F9FB.1 | XM 020337426.1 | ACD11 homolog protein(*Aegilops tauschii*) | 86% |
| Traes_4AL_F4A0C266E.1 | NULL | N/A |  |
| Traes_4AL_F4F41969B.1 | XM_020293992.1 | dynamin-related protein 1C(*Aegilops tauschii)* | 98% |
| Traes_4AL_F5C91EC4A.1 | XM 003576049.3 | protein NLP2-like(*Aegilops tauschii*) | 93% |
| Traes_4AL_F7B454B53.1 | XM 020298748.1 | cathepsin B-like(*Aegilops tauschii*) | 99% |
| Traes_4AL_F7D193897.1 | XR 002238294.1 | rho GTPase-activating protein 7-like (*Aegilops tauschii*) | 98% |
| Traes_4AL_F8F57B293.1 | XM 020316626.1 | GDSL esterase/lipase CPRD49(*Aegilops tauschii*) | 98% |
| Traes_4AL_F42D25A94.1 | XM 020327651.1 | F-box protein SKIP16(*Aegilops tauschii*) | 98% |
| Traes_4AL_F52F9F1BE.1 | XM 020306403.1 | lariat debranching enzyme (*Aegilops tauschii* ) | 97% |
| Traes_4AL_F75EDC0121.1 | XM 020333161.1 | transcription termination factor MTERF15, mitochondrial-like(*Aegilops*) | 96% |
| Traes_4AL_F93A8A1B0.1 | XM 020343961.1 | subtilisin-like protease SBT1.7(*Aegilops tauschii*) | 96% |
| Traes_4AL_F814B02F1.1 | XM_020312838.1 | caffeoylshikimate esterase(*Aegilops tauschii*) | 96% |
| Traes_4AL_F2150FCDC.1 | KT152849.1 | durum secretory phospholipase A2(*Triticum turgidum*) | 97% |
| Traes_4AL_F3338D25C.1 | XM 020331695.1 | N/A | 97% |
| Traes_4AL_F3571097B.1 | XM 020296806.1 | transcription factor bHLH18-like(*Aegilops tauschii*) | 97% |
| Traes_4AL_F46203719.1 | GU994117.1 | Chinese Spring phytochrome A type 2(*Triticum aestivum*) | 97% |
| Traes_4AL_FB1AA1388.1 | XM 020326082.1 | N/A | 94% |
| Traes_4AL_FB1F95CD6.2 | XM 020329660.1 | calcium-dependent protein kinase 9-like(*Aegilops tauschii*) | 97% |
| Traes_4AL_FB991D217.1 | XM 020300328.1 | CCR4-NOT transcription complex subunit 11-like(*Aegilops tauschii*) | 99% |
| Traes_4AL_FCF129CB0.1 | XM 020300864.1 | HIPL1 protein-like(*Aegilops tauschii*) | 94% |
| Traes_4AL_FD92BB31F.1 | XM_020293102.1 | 3-ketoacyl-CoA synthase 5-like(*Aegilops tauschii*) | 88% |
| Traes_4AL_FE0AC7213.2 | XM 020303442.1 | DNA-directed RNA polymerase III subunit RPC4-like(*Aegilops tauschii*) | 96% |
| Traes_4AL_FF24F9B94.1 | XM 020298746.1 | eukaryotic translation initiation factor 3 subunit G-like(*Aegilops tauschii*) | 97% |
| Traes_4AL_FF224CCF6.3 | XM 020298871.1 | protein DA1-related 1-like(*Aegilops tauschii*) | 97% |
| Traes_4AL_FF377A215.2 | XM 020308075.1 | tubulin beta-3 chain(*Aegilops tauschii*) | 95% |
| Traes_4AS_00A947384.1 | XM 020323338.1 | L10-interacting MYB domain-containing protein-like(*Aegilops tauschii*) | 97% |
| Traes_4AL_0A4AE3455.1 | XM 020312620.1 | DEAD-box ATP-dependent RNA helicase 52C-like(*Aegilops tauschii*) | 97% |
| Traes_4AS_0A061AB621.1 | XM 020336932.1 | pentatricopeptide repeat-containing protein At1g05750, chloroplastic-like(*Aegilops tauschii* ) | 99% |
| Traes_4AS_0AFA864E8.1 | XM 020299268.1 | protein EARLY-RESPONSIVE TO DEHYDRATION 7, chloroplastic-like(*Aegilops tauschii*) | 94% |
| Traes_4AS_0BCC125B5.3 | XM 020327710.1 | serine/threonine protein kinase OSK4(*Aegilops tauschii*) | 98% |
| Traes_4AS_0C71E39B4.1 | XM 0203377301.1 | probable 2-oxoglutarate-dependent dioxygenase At5g05600(*Aegilops tauschii*) | 99% |
| Traes_4AS_0DFC485F7.1 | XM 020310011.1 | polyubiquitin-like(*Aegilops tauschii*) | 97% |
| Traes_4AS_0FE9254551.3 | XM 020292688.1 | pentatricopeptide repeat-containing protein At1g62350(*Aegilops tauschii* ) | 98% |
| Traes_4AS_01F25BFF9.1 | XM 020344273.1 | rab GTPase-activating protein 1-lik(*Aegilops tauschii* ) | 94% |
| Traes_4AS_1D40BA8D2.1 | XM 020316855.1 | alpha-dioxygenase 1(*Aegilops tauschii*) | 99% |
| Traes_4AS_1DD18063D.1 | NULL | N/A |  |
| Traes_4AS_02D5EDA76.1 | XM 020342218.1 | probable 4-hydroxy-tetrahydrodipicolinate reductase 2, chloroplastic(*Aegilops tauschii*) | 95% |
| Traes_4AS_2A7B7986D.2 | XM 020338844.1 | protein SIEL(*Aegilops tauschii*) | 98% |
| Traes_4AS_2B3985A65.1 | XM 020344284.1 | glucose-6-phosphate 1-dehydrogenase 4, chloroplastic-like(*Aegilops tauschii*) | 96% |
| Traes_4AS_2BCB74FFD.1 | XM 020336744.1 | chaperone protein DnaJ-like(*Aegilops tauschii*) | 96% |
| Traes_4AS_2DCA42965.1 | AC134885.1 | Nipponbare strain, clone OJ1125B03, from chromosome 3(*Oryza sativa*) | 82% |
| Traes_4AS_2F8511C12.1 | KY636073.1 | muticum voucher 01C2100106 chloroplast(*Amblyopyrum*) | 98% |
| Traes_4AS_003E4E7C8.1 | XM_020340954.1 | N/A | 98% |
| Traes_4AS_3BF02DA54.1 | BN000704.1 | lar gene for leucanthocyanidin reductase(*Oryza sativa*) | 79% |
| Traes_4AS_3C32B4B06.1 | XM 020299300.1 | NHP2-like protein 1(*Aegilops tauschii*） | 98% |
| Traes_4AS_3CBD43E82.1 | XM 020322286.1 | mitochondrial phosphate carrier protein 3, mitochondrial-like(*Aegilops tauschii*) | 93% |
| Traes_4AS_3EBE9CF13.3 | XR 002236146.1 | UDP-glycosyltransferase 83A1-like(*Aegilops tauschii*) | 97% |
| Traes_4AS_3EFF6A752.2 | XM 020313017.1 | N/A | 98% |
| Traes_4AS_3FEDC3E8F.2 | XM 020343698.1 | ABC transporter F family member 4-like(*Aegilops tauschii*) | 94% |
| Traes_4AS_4A418E6B5.4 | XM 020344348.1 | protein FAR1-RELATED SEQUENCE 6-like(*Aegilops tauschii*) | 98% |
| Traes_4AS_4B1A64B8F.1 | XR 002231684.1 | arogenate dehydratase/prephenate dehydratase 2, chloroplastic-like(*Aegilops tauschii*) | 98% |
| Traes_4AS_4B20E1B7E.1 | XM 020341732.1 | filament-like plant protein 4(*Aegilops tauschii*) | 99% |
| Traes_4AS_4B236E907.2 | XM 020292463.1 | 40S ribosomal protein S21(*Aegilops tauschii*) | 99% |
| Traes_4AS_4BEF2DFAD.1 | XM 020300051.1 | sex determination protein tasselseed-2-like(*Aegilops tauschii*) | 98% |
| Traes_4AS_4C193E9D9.1 | XM 020337281.1 | protein PMR5-like (*Aegilops tauschii*) | 98% |
| Traes_4AS_4C529A3CF.1 | XM 020300799.1 | probable mitochondrial import inner membrane translocase subunit TIM21 (*Aegilops tauschii*) | 96% |
| Traes_4AS_4D52B3F9D.2 | XM 020291412.1 | nucleolin 2-like( *Aegilops tauschii* ) | 95% |
| Traes_4AS_4D943CB55.1 | XM 020316215.1 | guanine nucleotide exchange factor SPIKE 1(*Aegilops tauschii*) | 99% |
| Traes_4AS_5A87E55B4.1 | XM 020328440.1 | mitochondrial substrate carrier family protein V-like (*Aegilops tauschii*) | 99% |
| Traes_4AS_5A0162DFE.1 | XM 020317293.1 | N/A | 97% |
| Traes_4AS_5C64449F8.1 | XM 020317275.1 | bidirectional sugar transporter SWEET12-like(*Aegilops tauschii*) | 97% |
| Traes_4AS_5CAA94795.2 | XM 020292186.1 | DNA polymerase I(*Aegilops tauschii*) | 95% |
| Traes_4AS_5D1B30345.1 | XM 020291076.1 | N/A | 100% |
| Traes_4AS_5D090B0C6.2 | XM 020337244.1 | protein FAR-RED ELONGATED HYPOCOTYL 1-like(*Aegilops tauschii*) | 97% |
| Traes_4AS_5ED019F20.1 | XM 020300804.1 | N/A | 97% |
| Traes_4AS_6C236D93D.1 | XM 020338818.1 | N/A | 91% |
| Traes_4AS_6DA7ECA49.1 | XM 020292222.1 | serine/threonine-protein kinase STY8-like (*Aegilops tauschii*） | 96% |
| Traes_4AS_6DEC20069.1 | XM 020332747.1 | peroxidase A2-like(*Aegilops tauschii*) | 93% |
| Traes_4AS_6E7C4CDB4.1 | XM 020337876.1 | josephin-like protein(*Aegilops tauschii*) | 97% |
| Traes_4AS_6EDD5ACF7.2 | XM 020344286.1 | transcription factor TGA2.2(*Aegilops tauschii*) | 99% |
| Traes_4AS_7A955D191.2 | XR 002236973.1 | DNA (cytosine-5)-methyltransferase CMT2-like(*Aegilops tauschii*) | 98% |
| Traes_4AS_7F1606FAE.2 | XM 020343976.1 | autophagy-related protein 9 (*Aegilops tauschii*) | 96% |
| Traes_4AS_7FB4CF5D7.1 | XM 020322153.1 | mitochondrial succinate-fumarate transporter 1-like (*Aegilops tauschii*) | 96% |
| Traes_4AS_8A5A525A5.1 | XM 020325344.1 | patatin-like protein 6(*Aegilops tauschii*） | 99% |
| Traes_4AS_BCA501A4C.1 | XM 020315966.1 | transcription initiation factor IIF subunit alpha-like（*Aegilops tauschii*） | 99% |
| Traes_4AS_8CDDDA03A.2 | XM 020311729.1 | nuclear speckle splicing regulatory protein 1-like (*Aegilops tauschii*) | 94% |
| Traes_4AS_8CDF87CBD.2 | XM 020345362.1 | serine/arginine repetitive matrix protein 1( *Aegilops tauschii*) | 96% |
| Traes_4AS_8D6311711.1 | XM 020340057.1 | probable RNA-dependent RNA polymerase 2(*Aegilops tauschii*) | 97% |
| Traes_4AS_8E849E625.1 | XM 020317506.1 | N/A | 95% |
| Traes_4AS_9A0DF3368.1 | XM 020340960.1 | probable LL-diaminopimelate aminotransferase, chloroplastic(*Aegilops tauschii*) | 99% |
| Trase_4AS_9C1CB8D5A.2 | XM 020305584.1 | brefeldin A-inhibited guanine nucleotide-exchange protein 2-like(*Aegilops tauschii*） | 98% |
| Traes_4AS_9CDA229D8.1 | XR 002229864.1 | serine/arginine-rich-splicing factor SR34-like(*Aegilops tauschii*) | 99% |
| Traes_4AS_9D6002759.2 | XM 020339564.1 | phosphate metabolism protein 8-like(*Aegilops tauschii*) | 99% |
| Traes_4AS_9DCDB9778.1 | KF636828.1 | L318 chloroplast indole-3-glycerol phosphate lyase (Bx1) gene(*Secale cereale*) | 95% |
| Traes_4AS_9E5A45F1C.1 | XM 020336936.1 | probable pre-mRNA-splicing factor ATP-dependent RNA helicase DEAH3 (*Aegilops tauschii*） | 99% |
| Traes_4AS_9F4E1225F.1 | XM 020338851.1 | N/A | 98% |
| Traes_4AS_9F46EA4DC.1 | XM 020302847.1 | protein TOC75, chloroplastic(*Aegilops tauschii*) | 97% |
| Traes_4AS_12D136E9D.1 | XM_020292168.1 | glucan endo-1,3-beta-glucosidase 7-like(*Aegilops tauschii*） | 97% |
| Traes_4AS_14B87C3AE.1 | XM_020296836.1 | argininosuccinate lyase, chloroplastic(*Aegilops tauschii*) | 98% |
| Traes_4AS_19CDE03B6.1 | XM 020299291.1 | serine/threonine-protein kinase RIPK-like(*Aegilops tauschii*) | 97% |
| Traes_4AS_23A883CB4.1 | XM 020317500.1 | probable inactive poly [ADP-ribose] polymerase SRO3(*Aegilops tauschii*) | 98% |
| Traes_4AS_30E426CCC.1 | XM_020299414.1 | synaptotagmin-5-like(*Aegilops tauschii*） | 98% |
| Traes_4AS_35A112009.2 | XM_020296870.1 | universal stress protein PHOS32(*Aegilops tauschii)* | 95% |
| Traes_4AS_36F749D7B.2 | XM 020343973.1 | calmodulin-binding protein 60 D-like(*Aegilops tauschii*) | 98% |
| Traes_4AS_37DF23EFD.1 | XM_020296961.1 | N/A | 93% |
| Traes_4AS_37F805FF.1 | XM 020342947.1 | mitochondrial import receptor subunit TOM40-1-like(*Aegilops tauschii*) | 99% |
| Traes_4AS_40BDA4CD0.1 | XM 020325962.1 | acetate/butyrate--CoA ligase AAE7, peroxisomal-like(*Aegilops tauschii*) | 99% |
| Traes_4AS_46CFB0ADC.1 | XM 020338819.1 | protein MODIFIER OF SNC1 11(*Aegilops tauschii*) | 94% |
| Traes_4AS_46F94A43D.2 | XM 020297887.1 | chromosome transmission fidelity protein 18 homolog(*Aegilops tauschii*) | 99% |
| Traes_4AS_48CB85C72.1 | XM 020296889.1 | acetate/butyrate--CoA ligase AAE7, peroxisomal-like(*Aegilops tauschii)* | 97% |
| Traes_4AS_49FBFB97A.2 | XM_014900086.1 | paramyosin-like(*Brachypodium distachyon*) | 85% |
| Traes_4AS_050F32D60.1 | XM 020324373.1 | transcription factor bHLH148-like(*Aegilops tauschii*) | 97% |
| Traes_4AS_50F2C4B5D.1 | XM 020336937.1 | adenylosuccinate lyase-like(*Aegilops tauschii*) | 99% |
| Traes_4AS_051F2D921.2 | XM 004984686.1 | heat shock cognate 70 kDa protein-like(*Setaria italica*) | 77% |
| Traes_4AS_52EQDA341.2 | XM 020299711.1 | Fanconi anemia group I protein(*Aegilops tauschii*) | 97% |
| Traes_4AS_53D5BBB68.2 | XM 020332492.1 | NHL repeat-containing protein 2 (*Aegilops tauschii*) | 99% |
| Traes_4AS_54DE4BD86.1 | XM 020291077.1 | probable enoyl-CoA hydratase 1, peroxisomal(*Aegilops tauschii*) | 99% |
| Traes_4AS_55E5DA69A.2 | XR 002236973.1 | DNA (cytosine-5)-methyltransferase CMT2-like(*Aegilops tauschii*) | 97% |
| Traes_4AL_56D09C48C.1 | NULL | N/A |  |
| Traes_4AS_57A0BC13C.2 | XM 020318265.1 | inositol-tetrakisphosphate 1-kinase 2-like(*Aegilops tauschii*) | 100% |
| Traes_4AS_59EF3B3A2.1 | XM 020333909.1 | mitogen-activated protein kinase kinase kinase 1-like(*Aegilops tauschii*) | 94% |
| Traes_4AS_063A9B72A.2 | XM 020324307.1 | protein RIK (*Aegilops tauschii*) | 97% |
| Traes_4AS_71AA07545.1 | XM 020302196.1 | N/A | 95% |
| Traes_4AS_73EA366EE.1 | XM 020320298.1 | probable leucine-rich repeat receptor-like protein kinase At5g49770(*Aegilops tauschii*) | 96% |
| Traes_4AS_74EE1FD0E.1 | XM_020299417.1 | V-type proton ATPase subunit a3-like(*Aegilops tauschii*) | 99% |
| Traes_4AS_78CFEFB64.1 | XM 020299705.1 | mucin-5AC(*Aegilops tauschii*) | 99% |
| Traes_4AS_80A9FCCF4.1 | XM 020320299.1 | probable dolichyl pyrophosphate Man9GlcNAc2 alpha-1,3-glucosyltransferase(*Aegilops tauschii* ) | 99% |
| Traes_4AS_85A2DD5D9.1 | XM_020328454.1 | N/A | 91% |
| Traes_4AS_85EA00464.1 | XM_020322002.1 | J domain-containing protein spf31(*Aegilops tauschii*) | 97% |
| Traes_4AS_85F059C27.1 | XM 020317863.1 | probable galacturonosyltransferase 7(*Aegilops tauschii*) | 99% |
| Traes_4AS_85F95BF68.2 | XR_002237300.1 | N/A | 95% |
| Traes_4AS_88F47105E.1 | XM_020344051.1 | N/A | 98% |
| Traes_4AS_90CC29CAA.2 | XM_020320280.1 | uroporphyrinogen decarboxylase (*Aegilops tauschii*) | 98% |
| Traes_4AS_94D7A563D.2 | XM 020332734.1 | vegetative cell wall protein gp1-like(*Aegilops tauschii*) | 91% |
| Traes_4AS_95C493717.2 | XM 020315425.1 | E3 ubiquitin-protein ligase XB3-like(*Aegilops tauschii*) | 97% |
| Traes_4AS_96A1B58D9.1 | XM 020336846.1 | sister-chromatid cohesion protein 3(*Aegilops tauschii*) | 99% |
| Traes_4AS_96F05E5DA.1 | XM 020336927.1 | N/A | 96% |
| Traes_4AS_97A5D8F2B.2 | XM_020338850.1 | N/A | 99% |
| Traes_4AS_97BB42724.1 | HG670306.1 | chromosome 3B, genomic scaffold, cultivar Chinese Spring(*Triticum aestivum*) | 90% |
| Traes_4AS_97D5BBDE7.2 | XM 020332494.1 | NHL repeat-containing protein 2(*Aegilops tauschii*) | 95% |
| Traes_4AS_151E507DC.1 | XM 020344440.1 | N/A | 83% |
| Traes_4AS_167FECEB8.3 | XM 020296612.1 | N/A | 98% |
| Traes_4AS_174B77945.2 | XM 020337297.1 | phosphoinositide phosphatase SAC8(*Aegilops tauschii*) | 99% |
| Traes_4AS_177B17C02.2 | XM 020330517.1 | probable pre-mRNA-splicing factor ATP-dependent RNA helicase DEAH5(*Aegilops tauschii*) | 98% |
| Traes_4AS_184AF3824.1 | XM 020301924.1 | N/A | 99% |
| Traes_4AS_189C692A4.1 | XM 020298183.1 | N/A | 99% |
| Traes_4AS_254B6D654.4 | FM211849.1 | partial pck gene for phosphoenolpyruvate carboxykinase(*Holcus lanatus*) | 83% |
| Traes_4AS_265BF3644.1 | XM 020337255.1 | N/A | 97% |
| Traes_4AS_352D6953E.1 | XM 020321373.1 | N/A | 96% |
| Traes_4AS_361ADDD17.1 | XM_020292686.1 | probable leucine-rich repeat receptor-like protein kinase IMK3(*Aegilops tauschii*) | 97% |
| Traes_4AS_0402C0FA6.1 | XM_020325343.1 | tauschii protein TPR3(*Aegilops tauschii*) | 98% |
| Traes_4AS_424A4DD16.1 | XM_020342225.1 | 3-ketoacyl-CoA synthase 1-like(*Aegilops tauschii*) | 97% |
| Traes_4AS_477F821FF.1 | XM 020317273.1 | N/A | 97% |
| Traes_4AS_512DDDECB.2 | JQ791249.1 | vulgare cultivar OWB-R P (P) gene(*Hordeum vulgare*) | 93% |
| Traes_4AS_513DFF78C.1 | KX533924.1 | eukaryotic elongation factor eEF1A mRNA(*Triticum aestivum*) | 100% |
| Traes_4AS_538F2E3BD.2 | AC119748.1 | Nipponbare strain, clone OSJNBa0042L15, from chromosome 3(*Oryza sativa*) | 77% |
| Traes_4AS_547AD04C7.2 | XM_020344049.1 | N/A | 94% |
| Traes_4AS_553C18F16.1 | KX5331804.1 | GTP-binding protein mRNA(*Triticum aestivum*) | 100% |
| Traes_4AS_636DF8B4C.2 | XM 020320499.1 | N/A | 98% |
| Traes_4AS_702A3C7D6.1 | XM 020337875.1 | sulfoquinovosyl transferase SQD2-like(*Aegilops tauschii*) | 97% |
| Traes_4AS_707D51357.1 | XR 002226320.1 | probable linoleate 9S-lipoxygenase 4(*Aegilops tauschii*) | 98% |
| Traes_4AS_759D8DDBB.1 | XM 020299703.1 | transcription factor MYB44-like(*Aegilops tauschii*) | 98% |
| Traes_4AS_790C94D16.2 | HG670306.1 | chromosome 3B, genomic scaffold, cultivar Chinese Spring(*Triticum aestivum*) | 92% |
| Traes_4AS_830B0A199.1 | XM_020320279.1 | transcription repressor OFP1-like(*Aegilops tauschii*) | 94% |
| Traes_4AS_906AA4054.1 | KT152848.1 | durum secretory phospholipase A2(*Triticum turgidum*) | 99% |
| Traes_4AS_910FE216D1.2 | XM 020299901.1 | protein LURP-one-related 11-like(*Aegilops tauschii*) | 93% |
| Traes_4AS_927B66A08.1 | AC213123.1 | clone OG_BBa0005O15(*Oryza glaberrima* ) | 80% |
| Traes_4AS_934C6302E.1 | XM 020328433.1 | digalactosyldiacylglycerol synthase 2, chloroplastic-like(*Aegilops tauschii*) | 98% |
| Traes_4AS_2035C95B5.1 | XR 002233814.1 | pentatricopeptide repeat-containing protein At5g65560(*Aegilops tauschii*) | 94% |
| Traes_4AS_2272D0413.1 | LT220907.1 | durum Hsp26-A2Ch gene for heat shock protein(*Triticum turgidum*) | 99% |
| Traes_4AS_2558D388E.1 | XM 020343974.1 | ACT domain-containing protein ACR8-like(*Aegilops tauschii*) | 96% |
| Traes_4AS_3091DDF0C.3 | XR 002228824.1 | N/A | 92% |
| Traes_4AS_3255E67D0.1 | XM 020340963.1 | LYR motif-containing protein 4(*Aegilops tauschii*) | 99% |
| Traes_4AS_3330C80FB.1 | KY636171.1 | voucher PI 428093 chloroplast(*Triticum turgidum* ) | 99% |
| Traes_4AS_3517E5EA1.3 | XM 020333848.1 | N/A | 98% |
| Traes_4AS_3883DC244.1 | KU674901.1 | RAC875 MYB transcription factor 78(*Triticum aestivum* ) | 100% |
| Traes_4AS_4190CCF4C.1 | XM 020335551.1 | N/A | 98% |
| Traes_4AS_4693A1150.2 | XM 020296610.1 | eukaryotic translation initiation factor 2 subunit alpha homolog(*Aegilops tauschii*) | 98% |
| Traes_4AS_50515DF7A2.1 | AF079318.1 | MAP kinase homolog (WCK-1) mRNA(*Triticum aestivum*) | 99% |
| Traes_4AS_5948AF2B5.1 | XM 020290594.1 | serine/threonine-protein phosphatase 7 long form homolog(*Aegilops tauschii*) | 91% |
| Traes_4AS_5971FF832.1 | XM 020304831.1 | LOB domain-containing protein 30(*Aegilops tauschii*) | 98% |
| Traes_4AS_6262F990C.1 | XM 020342222.1 | zinc finger BED domain-containing protein RICESLEEPER 2-like(*Aegilops tauschii*) | 99% |
| Traes_4AS_6285AE7F6.1 | XM 020300037.1 | 26S protease regulatory subunit 4 homolog(*Aegilops tauschii*) | 99% |
| Traes_4AS_8398CBD3E.2 | XM 020299299.1 | vegetative cell wall protein gp1-like(*Aegilops tauschii*) | 97% |
| Traes_4AS_8527E5F3D.1 | XM 020338848.1 | cytochrome P450 72A15-like(*Aegilops tauschii*) | 98% |
| Traes_4AS_9414F63D7.2 | XM 020301932.1 | histone-lysine N-methyltransferase EZ3-like (*Aegilops tauschii*) | 99% |
| Traes_4AS_031757A2A.2 | XM 020291909.1 | probable inactive receptor kinase At5g58300(*Aegilops tauschii*) | 98% |
| Traes_4AS_42748DD6E.1 | XM 020328433.1 | digalactosyldiacylglycerol synthase 2, chloroplastic-like(*Aegilops tauschii*) | 97% |
| Traes_4AS_53905F273.2 | XM 020296506.1 | ankyrin repeat-containing protein At5g02620(*Aegilops tauschii*) | 98% |
| Trase_4AS_73658DB91.1 | GU902795.1 | nuclear transcription factor Y subunit C11 mRNA(*Triticum monococcum* ) | 98% |
| Traes_4AS_74660DF99.2 | XM 020322270.1 | splicing factor 3A subunit 2(*Aegilops tauschii*) | 98% |
| Traes_4AS_147020D73.1 | XM 020299709.1 | 60S ribosomal protein L18-3-like(*Aegilops tauschii*) | 96% |
| Traes_4AS_296775EE4.1 | XM 020301933.1 | protein trichome birefringence-like 6(*Aegilops tauschii*) | 88% |
| Traes_4AS_753213AF4.1 | XM 020302740.1 | aldehyde oxidase GLOX-like(*Aegilops tauschii*) | 94% |
| Traes_4AS_794263F92.1 | XM 020316217.1 | cytosolic enolase 3(*Aegilops tauschii*) | 99% |
| Traes_4AS_936532D35.2 | AC137267.2 | Nipponbare strain, clone OSJNBa0032G21(*Oryza sativa*) | 81% |
| Traes_4AS_7520725F7.1 | XM 020309365.1 | probable glycosyltransferase 5(*Aegilops tauschii*) | 99% |
| Traes_4AS_9662391A9.2 | XM 020340942.1 | rho GTPase-activating protein REN1-like(*Aegilops tauschii*) | 99% |
| Traes_4AS_32255850B.1 | XM 020320975.1 | N/A | 99% |
| Traes_4AS_60155447D.2 | AC137072.2 | Nipponbare strain, clone OSJNBa0045E22(*Oryza sativa*) | 82% |
| Traes_4AS_271282074.1 | XM 020340984.1 | N/A | 96% |
| Traes_4AS_326265729.1 | XM 020299823.1 | gamma-interferon-inducible lysosomal thiol reductase-like(*Aegilops tauschii*) | 91% |
| Traes_4AS_341045223.1 | XM 020291074.1 | UPF0481 protein At3g47200-like(*Aegilops tauschii*) | 97% |
| Traes_4AS_812896652.1 | XM 020342210.1 | proline-rich protein 4-like(*Aegilops tauschii*) | 96% |
| Traes_4AS_A0CE022D5.1 | XR 002231685.1 | arogenate dehydratase/prephenate dehydratase 2, chloroplastic-like(*Aegilops tauschii*) | 90% |
| Traes_4AS_A03AC635D.1 | AC134234.3 | chromosome 3 clone OSJNBa0019J12(*Oryza sativa*) | 79% |
| Traes_4AS_A14EE4E74.1 | XM 020304547.1 | serine/threonine-protein phosphatase 7 long form homolog(*Aegilops tauschii*) | 92% |
| Traes_4AS_A538B5587.1 | XM 020341740.1 | histone H4(*Aegilops tauschii*) | 98% |
| Traes_4AS_A6128FCF8.7 | NULL | N/A |  |
| Traes_4AS_A87472562.3 | XR 002232562.1 | N/A | 87% |
| Traes_4AS_A97064408.2 | XM 020343757.1 | pentatricopeptide repeat-containing protein At5g50990-like*(Aegilops tauschii )* | 97% |
| Traes_4AS_AAF6B6BDF.2 | xm 020292160.1 | coatomer subunit gamma-1 *(Aegilops tauschii)* | 99% |
| Traes_4AS_AAF8A8CE7.1 | XM 020324459.1 | N/A | 96% |
| Traes_4AS_AB1EE500C.1 | XM 020344275.1 | serine/threonine-protein kinase PBL27-like *(Aegilops tauschii)* | 96% |
| Traes_4AS_AB0047421.1 | XM 020343938.1 | N/A | 96% |
| Traes_4AS_ABE7CBE39.1 | XM 020322510.1 | putative serine/threonine-protein kinase*( Aegilops tauschii)* | 97% |
| Trase_4AS_AC4E8530D.1 | XM 020315119.1 | protein MALE DISCOVERER 2-like *(Aegilops tauschii)* | 98% |
| Traes_4AS_AC029DCD3.1 | XM 020326964.1 | N/A | 96% |
| Traes_4AS_AC5746DFA.1 | XR 002230757.1 | N/A | 84% |
| Traes_4AS_AEE55FE7F.1 | XM 020291987.1 | probable linoleate 9S-lipoxygenase 4*(Aegilops tauschii )* | 96% |
| Traes_4AS_B1DE7ABF8.2 | XM 020292462.1 | cell cycle checkpoint control protein RAD9B *(Aegilops tauschii)* | 98% |
| Traes_4AS_B2CDBFB54.1 | XM 020344274.1 | two-component response regulator-like PRR73*(Aegilops tauschii )* | 96% |
| Traes-4AS_B3AE0219F.1 | XR 002225488.1 | N/A | 96% |
| Traes_4AS_B4E9D81C4.1 | xm 020322448.1 | cyclin-A3-2-like*(Aegilops tauschii )* | 96% |
| Traes_4AS_B5D20D324.1 | XM 020332731.1 | metal tolerance protein 4*(Aegilops tauschii)* | 100% |
| Traes_4AS_B9CD186CE.2 | XM 020332737.1 | protein NRT1/ PTR FAMILY 8.3-like*(Aegilops tauschii )* | 98% |
| Traes_4AS_B9F2F6646.1 | XM 020299653.1 | hydroxyacylglutathione hydrolase cytoplasmic*(Aegilops tauschii )* | 98% |
| Traes_4AS_B059A2469.1 | AB255441.1 | aegilopoides TbBx2-1 mRNA for cytochrome P450*(Triticum monococcum)* | 99% |
| Traes_4AS_B62A2623C.1 | XM 020343974.1 | ACT domain-containing protein ACR8-like*( Aegilops tauschii )* | 98% |
| Traes_4AS_B95A1C3A1.1 | XM 020306960.1 | NAC domain-containing protein 92-like *(Aegilops tauschii )* | 97% |
| Traes_4AS_B1246A1B5.1 | XM 020322449.1 | probable 2-oxoglutarate-dependent dioxygenase AOP1.2 *(Aegilops tauschii)* | 95% |
| Traes_4AS_B7069E02A.1 | XM 020334402.1 | N/A | 98% |
| Traes_4AS_B98583F96.1 | NULL | N/A |  |
| Traes_4AS_B163648BA.5 | AK252022.1 | vulgare cDNA clone: FLbaf140g05*(Hordeum vulgare )* | 87% |
| Trase_4AS_B509831CB.1 | XM 020333483.1 | probable arabinosyltransferase ARAD1*(Aegilops tauschii )* | 98% |
| Traes_4AS_B984297FD.2 | XM 020318362.1 | IST1 homolog*(Aegilops tauschii)* | 98% |
| Traes_4AS_BA08E293.1 | XM 020334469.1 | receptor-like serine/threonine-protein kinase At2g45590 *(Aegilops tauschii )* | 98% |
| Traes_4AS_BB6F6B4E9.1 | AK335316.1 | cDNA, clone: WT012_J14Chinese Spring(*Triticum aestivum* ) | 100% |
| Traes_4AS_BCBF7C67C.1 | AK376779.1 | vulgare mRNA for predicted protein, complete cds, clone: NIASHv3135O03(Hordeum) | 95% |
| Traes_4AS_BE41B7C1F.1 | AP017300.1 | spontaneum mitochondrial DNA, complete sequence, strain: H602 *(Hordeum)* | 100% |
| Trase_4AS_C3F277DE5.1 | AC129008.1 | Nipponbare strain, clone OSJNBb0096L14, from chromosome 3*( Oryza sativa )* | 85% |
| Traes_4AS_C7DE35DC8.1 | XM 020332750.1 | pentatricopeptide repeat-containing protein At4g37170*(Aegilops tauschii)* | 95% |
| Traes_4AS_C23F9BFB9.1 | JF683316.1 | aestivum 5-methylcytosine DNA glycosylase *(Triticum aestivum)* | 100% |
| Traes_4AS_C34BBA0EB.2 | XM 020314944.1 | zinc finger MYM-type protein 1-like *(Aegilops tauschii)* | 92% |
| Traes_4AS_C680EF5AB.1 | XM 020336928.1 | nifU-like protein 1, chloroplastic*(Aegilops tauschii)* | 99% |
| Traes_4AS_C839DCF3A.1 | AC083942.8 | Nipponbare strain, clone OSJNBa0002D01, from chromosome 3*(Oryza sativa)* | 76% |
| Traes_4AS_C68517AC8.1 | XM 020332734.1 | vegetative cell wall protein gp1-like*(Aegilops tauschii)* | 98% |
| Traes_4AS_CB2B444C5.1 | XM 020341448.1 | F-box/LRR-repeat protein 4 *(Aegilops tauschii )* | 98% |
| Traes_4AS_CB3F89616.1 | XM 020333910.1 | partner of Y14 and mago*(Aegilops tauschii)* | 96% |
| Traes_4AS_CBBAA0282.2 | AY146587.2 | turgidum subsp. durum Pm3 locus*(Triticum turgidum )* | 90% |
| Traes_4AS_CDE15123E.1 | XM 020341146.1 | N/A | 99% |
| Traes_4AS_CECD2CB54.2 | XM 020318885.1 | peroxidase 70-like*(Aegilops tauschii)* | 97% |
| Traes_4AS_D5B85A7FB.1 | XM 020340942.1 | rho GTPase-activating protein REN1-like*(Aegilops tauschii )* | 98% |
| Traes_4AS_D5F8B2031.2 | XR 002227301.1 | N/A | 91% |
| Traes_4AS_D6E6B310D.1 | XM 020311741.1 | subtilisin-like protease SBT1.6*(Aegilops tauschii)* | 99% |
| Traes_4AS_D20DF472E.1 | XM 020333856.1 | zinc finger protein 7-like*( Aegilops tauschii )* | 95% |
| Traes_4AS_D35C2744B.2 | XM 020344282.1 | pentatricopeptide repeat-containing protein At1g56690, mitochondrial(*Aegilops tauschii)* | 99% |
| Traes_4AS_D45DE38D0.1 | XM 020294925.1 | DDB1- and CUL4-associated factor 8 *(Aegilops tauschii )* | 98% |
| Traes_4AS_D83CE355A.1 | XR 002232674.1 | N/A | 92% |
| Traes_4AS_D356C7AB3.2 | XM 020337224.1 | protein DETOXIFICATION 48-like *(Aegilops tauschii)* | 96% |
| Traes_4AS_D533B306B.1 | XM 020292465.1 | probable E3 ubiquitin-protein ligase ARI2*(Aegilops tauschii )* | 98% |
| Traes_4AS_D4763E468.1 | XM 004984705.2 | zinc finger protein ZAT11*(Setaria italica)* | 81% |
| Traes_4AS_D7511DC6D.1 | XM 020315997.1 | auxin transporter-like protein 2 *(Aegilops tauschii)* | 99% |
| Traes_4AS_D81176DF4.1 | XM 020320844.1 | ABC transporter G family member 5-like*(Aegilops tauschii )* | 98% |
| Traes_4AS_D93610E94.1 | XM 020302851.1 | N/A | 97% |
| Traes_4AS_D34648138.1 | XM_020331760.1 | glucan endo-1,3-beta-glucosidase 10-like *(Aegilops tauschii)* | 97% |
| Traes_4AS_DA5C35199.1 | XM_020315966.1 | transcription initiation factor IIF subunit alpha-like*(Aegilops tauschii)* | 93% |
| Traes_4AS_DA6AF313C.1 | XM_020291073.1 | GDSL esterase/lipase At4g16230-like *(Aegilops tauschii )* | 99% |
| Traes_4AS_DAC5B30A3.1 | KP844884.1 | Chara PM19-A1*(Triticum aestivum )* | 89% |
| Traes_4AS_DAD8FB0D2.1 | SNF-1 | SNF1-related protein kinase regulatory subunit beta-1-like*(Aegilops tauschii )* | 97% |
| Traes_4AS_DC872C10F.1 | XM 02030658.1 | laccase-10-like*(Aegilops tauschii )* | 97% |
| Traes_4AS_DE644E0CF.1 | XM 020302848.1 | RNA polymerase sigma factor sigB-like*(Aegilops tauschii)* | 97% |
| Traes_4AS_DF85CBD39.2 | XM 020328432.1 | serine/threonine-protein kinase ATG1c-like*(Aegilops tauschii )* | 100% |
| Traes_4AS_E01B18DB6.4 | XR 002231856.1 | N/A | 97% |
| Traes_4AS_E2D1D9E5D.1 | XM 020321372.1 | calmodulin-like*(Aegilops tauschii)* | 97% |
| Traes_4AS_E4CD9DE35.1 | XM 020299416.1 | IRK-interacting protein(*Aegilops tauschii)* | 97% |
| Traes_4AS_E7F252662.1 | XM 020292688.1 | pentatricopeptide repeat-containing protein At1g62350*(Aegilops tauschii )* | 99% |
| Traes_4AS_E28B34320.1 | XM_020343661.1 | serine/arginine repetitive matrix protein 2-like*(Aegilops tauschii)* | 97% |
| Traes_4AS_E37A0792D.1 | XM_020299415.1 | chloride conductance regulatory protein Icln*(Aegilops tauschii)* | 98% |
| Traes_4AS_E41A94443.1 | XM 020328439.1 | protein DA1-related 2*(Aegilops tauschii)* | 99% |
| Traes_4AS_E78B72F20.1 | XM 020302429.1 | putative pentatricopeptide repeat-containing protein At1g02420 *(Aegilops tauschii)* | 97% |
| Traes_4AS_E3238CBB7.2 | XM 020302850.1 | pentatricopeptide repeat-containing protein At5g59600*(Aegilops tauschii )* | 96% |
| Traes_4AS_E960B83C6.1 | XM 020345360.1 | AT-hook motif nuclear-localized protein 29-like*(Aegilops tauschii )* | 98% |
| Traes_4AS_E3238CBB7.2 | KF427372.1 | voucher MPF10163*(Phyllostachys edulis)* | 76% |
| Traes_4AS_E4294BADC.1 | XM 020342599.1 | transcription factor MYB86-like*(Aegilops tauschii )* | 98% |
| Traes_4AS_E5556AFAD.1 | XM 020317264.1 | DEAD-box ATP-dependent RNA helicase 24 *(Aegilops tauschii )* | 98% |
| Traes_4AS_E459380C5.1 | XM 020315108.1 | ethylene-overproduction protein 1 *(Aegilops tauschii)* | 98% |
| Traes_4AS_EA15000ED.2 | XM 020322760.1 | very-long-chain (3R)-3-hydroxyacyl-CoA dehydratase PASTICCINO 2A-like*(Aegilops tauschii)* | 96% |
| Traes_4AS_EB18E9A3D.1 | XM 020299270.1 | proline-rich receptor-like protein kinase PERK1 *(Aegilops tauschii)* | 96% |
| Traes_4AS_EC6DAA3AE.2 | XM 020316218.1 | LIM domain-containing protein WLIM2a-like*(Aegilops tauschii)* | 98% |
| Traes_4AS_ED8D7EE94.1 | AC137696.2 | Nipponbare strain, clone OSJNBa0049C20, from chromosome 3*( Oryza sativa)* | 86% |
| Traes_4AS_ED397786C.1 | XM 020292223.1 | NADH dehydrogenase [ubiquinone] 1 alpha subcomplex subunit 8-B-like*(Aegilops tauschii)* | 98% |
| Traes_4AS_EEAF4CDB7.1 | XM 020334401.1 | N/A | 98% |
| Traes_4AS_F1F72C66C.1 | XM 020292521.1 | GTP-binding protein At2g22870*(Aegilops tauschii)* | 96% |
| Traes_4AS_F8DB6475B.2 | XM 020325341.1 | probable E3 ubiquitin-protein ligase LUL2*(Aegilops tauschii )* | 98% |
| Traes_4AS_F9C171219.1 | XM 020342494.1 | ABSCISIC ACID-INSENSITIVE 5-like protein 2*(Aegilops tauschii)* | 96% |
| Traes_4AS_F40BF689C.2 | XM_020342224.1 | N/A | 97% |
| Traes_4AS_F51C2F6FC.1 | XM_020337996.1 | serine/threonine-protein phosphatase 7 long form homolog(*Aegilops tauschii* ) | 79% |
| Traes_4AS_F71C13C9B.1 | XM 020322509.1 | anthranilate synthase alpha subunit 2, chloroplastic*(Aegilops tauschii)* | 98% |
| Traes_4AS_F238AFB04.1 | XM 020327192.1 | N/A | 83% |
| Traes_4AS_F516F49FA.4 | XM 020340959.1 | wound-induced protein 1*(Aegilops tauschii)* | 96% |
| Traes_4AS_F4613A030.1 | XM 010242133.2 | bromodomain and WD repeat-containing DDB_G0285837*( Brachypodium distachyon)* | 88% |
| Traes_4AS_F7794E944.2 | GQ412260.1 | aestivum phosphomannomutase A2*(Triticum)* | 99% |
| Traes_4AS_F28333B821.2 | XM 020298614.1 | N/A | 97% |
| Traes_4AS_F91240F31.1 | XM 020335490.1 | N/A | 74% |
| Traes_4AS_FA3D49516.1 | XM 020332735.1 | peroxidase N-like*（ Aegilops tauschii）* | 98% |
| Traes_4AS_FBBB407B9.1 | XM 020344276.1 | elongator complex protein 6 *（ Aegilops tauschii）* | 98% |
| Traes_4AS_FBE6E9974.1 | HG670306.1 | chromosome 3B, genomic scaffold, cultivar Chinese Spring(*Triticum aestivum)* | 92% |
| Traes_4AS_FFAB3EACB.2 | XM 020320502.1 | N/A | 98% |
| Traes_4AS_FFBD288B6.1 | XM 020317504.1 | protein NRT1/ PTR FAMILY 8.3-like *（Aegilops tauschii）* | 96% |
